# Proteomic Screening for Cellular Targets of the Duck Enteritis Virus Protein VP26 Reveals That the Host Actin–Myosin II Network Regulates the Proliferation of the Virus

**DOI:** 10.3390/ijms26189108

**Published:** 2025-09-18

**Authors:** Liu Chen, Yin-Chu Zhu, Tao Yun, Wei-Cheng Ye, Zheng Ni, Jiong-Gang Hua, Cun Zhang

**Affiliations:** State Key Laboratory for Quality and Safety of Agro-Products, Institute of Animal Husbandry and Veterinary Sciences, Zhejiang Academy of Agricultural Sciences, Hangzhou 310021, China

**Keywords:** duck enteritis virus, VP26, interactome, cytoskeleton actin filament, *MYH9*

## Abstract

Duck enteritis virus (DEV) is responsible for duck viral enteritis, a contagious and lethal disease in waterfowls. The host proteins targeted by DEV are unknown. In this study, we developed a recombinant DEV rVP26-Flag and identified 17 host proteins that interact with VP26 in infected chicken embryo fibroblast cells using co-immunoprecipitation in conjunction with liquid chromatography–tandem mass spectrometry (Co-IP-MS/MS). The 17 potential targets of VP26 proteins include Xirp1, TMOD3, DCN, ATP5PD, AP3M1, MYO5A, MYH10, MYH9 (non-muscle myosin IIA heavy chain), and GSN. Most of these proteins are microfilament or cytoskeletal proteins with functions such as cytoskeletal protein binding, actin filament interaction, microfilament motor activity, and myosin II interaction. Using the Search Tool for the Retrieval of Interacting Genes analysis, we predicted a functional network of microfilament cytoskeletal proteins interacting with VP26. Interaction between DEV VP26 and the carboxyl-terminus domain of MYH9 (1651–1960 aa) was verified via co-localization and Co-IP assays. We also demonstrated that the inhibition of actin polymerization with cytochalasin D and latrunculin A reduced the DEV titer. Furthermore, siRNA-mediated knockdown of MYH9, which has intrinsic ATPase activity, also resulted in a reduced viral titer. A targeted inhibitor of myosin II ATPase, (-)-Blebbistatin, significantly suppressed DEV infection both in vitro and in vivo. These results suggest that the actin–myosin II network plays a crucial role in DEV proliferation, with MYH9 being an important host factor influencing DEV infection.

## 1. Introduction

Duck viral enteritis, also known as duck plague, is a highly contagious and lethal disease in Anseriformes (waterfowl) caused by duck enteritis virus (DEV), a member of the Alphaherpesvirinae subfamily [1]. The virus is distributed throughout various tissues and organs post-infection, leading to severe pathological changes. The virus consists of an envelope, capsid, tegument, and double-stranded DNA genome, which is estimated to encode 78 functional proteins, including 11 envelope glycoproteins (gB/UL27, gC/UL44, gD/US6, gE/US8, gG/US4, gH/UL22, gI/US7, gK/UL53, gL/UL1, gM/UL10, and gN/UL49.5) [2,3] and 7 capsid proteins (VP19C/UL38, VP23/UL18, VP26/UL35, UL21/UL21, UL25/UL25, VP5/UL19, and UL6/UL6) [2,4]. Among these, VP5 is a major capsid protein that forms pentons and hexons, while VP26, the smallest capsid protein (12 kDa), is associated with the VP5-formed hexons but not pentons.

VP26 plays an important role in the pathogenicity and replication of herpes simplex virus I (HSV-1), as UL35 deletion seriously impairs viral replication in the central nervous system of infected mice [5]. VP26 is also necessary for the successful replication and virulence of pseudorabies virus (PRV) in vivo and in cell culture [6]. Our recent research demonstrated that the titer of a UL35-deleted DEV decreased both in vitro and in vivo, and the lethality rate decreased by 93% in ducklings and 100% in adult ducks compared with that of the wild-type virus (unpublished data), indicating that VP26 is essential for the replication and virulence of DEV. The capsid of a VP26-deficient recombinant herpesvirus failed to enter the nucleus after being microinjected into Hep-2 cells [7], suggesting that VP26 is essential for the nuclear translocation of the herpesvirus capsids. Recent studies have revealed that HSV-1 VP26 is critical for the effective packaging of viral DNA, the correct localization of the nucleocapsid protein VP5, and nucleocapsid maturation [8,9]. However, some controversial results have been reported regarding the role of VP26 in transporting the viral capsid to the nucleus. A VP26-deficient HSV-1 mutant can be transported retrogradely to the sensory ganglia of the murine cornea following inoculation, indicating that VP26 is not required for capsid-based nuclear targeting [5]. Similarly, PRV’s VP26 is also not necessary for nuclear targeting [10].

The interaction between VP26 and the host cytoskeleton has been a subject of intense study. Cytoplasmic dynein, an essential biological motor that controls minus-end-directed cellular transport along microtubules, has been shown to interact with HSV-1 VP26, particularly through its light chains RP3 and Tctex1, thereby modulating the retrograde cellular transport [7]. Upon PRV infection, actin filaments form within the cell nucleus, followed by viral site assembly and the co-localization of VP26 and myosin in the nucleus, suggesting that viral capsid transport along the nuclear actin filaments is mediated by myosin [11].

In this study, we used co-immunoprecipitation in conjunction with liquid chromatography–tandem mass spectrometry (CoIP-MS/MS) to extract VP26-associated host proteins from rVP26-Flag-infected chicken embryo fibroblast cells. Notably, the proteomics approach offers unique advantages for revealing DEV’s virulence characteristics because it enables unbiased, global profiling of both host and viral protein dynamics, capturing coordinated molecular events that underpin DEV’s rapid systemic spread and pathogenicity. Next, employing STRING (Search Tool for the Retrieval of Interacting Genes) analysis, we found that most VP26-interacting proteins formed a functionally interconnected network of modulatory and actin-interacting proteins. We also demonstrated that the actin polymerization inhibitors cytochalasin D and latrunculin A independently diminished DEV titers. Similarly, the siRNA-based MYH9 (non-muscle myosin IIA heavy chain, a member of the myosin–kinesin ATPase superfamily) knockdowns also reduced viral titers. In addition, a targeted myosin II ATPase inhibitor, (-)-Blebbistatin (BLEB), suppressed DEV infection both in vitro and in vivo. Our data demonstrate that the actin–myosin II network plays a crucial role in DEV proliferation, with MYH9 being an important host factor influencing DEV infection.

## 2. Results

### 2.1. Validation of VP26 Expression in Recombinant Virus-Infected Cells Using Western Blot Analysis

**Figure 1:**
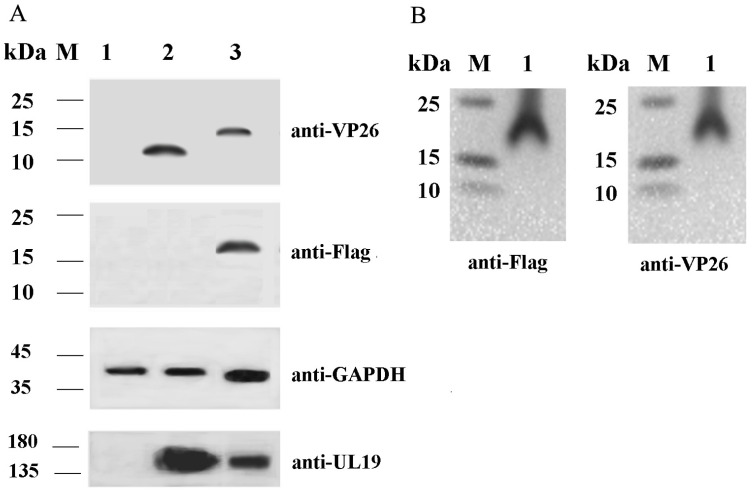
Western blot analysis of VP26 in virus-infected cells (**A**) and purified rVP26-Flag virions (**B**). (**A**) CEF cells were infected with rE1 or rVP26-Flag for 48 h, and the cells were collected and taken for Western blot analysis. M: prestained protein marker; 1: CEF cells; 2: rE1-infected CEF cells; 3: rVP26-Flag-infected CEF cells. (**B**) rVP26-Flag-infected CEF cells and supernatants were collected at 48 h, and rVP26-Flag was purified via sucrose density gradient centrifugation; then, purified virions were used for Western blot analysis. M: prestained protein marker; 1: purified rVP26-Flag.

We generated the recombinant DEV strain rVP26-Flag by tagging a Flag epitope at the C-terminus of the VP26 protein. These genetic modifications exerted a slight effect on the virus′s growth kinetics, including its replication efficiency in host cells and plaque-forming capacity (Supplementary Figures S1 and S2). To validate these recombinant viruses, we infected chicken embryo fibroblast (CEF) cells with rE1 or rVP26-Flag at a multiplicity of infection (MOI) of 1.0 and evaluated VP26 expression in the cells via Western blot analysis using a VP26-specific antibody. Our data showed that cells inoculated with rE1 exhibited a unique 12.9 kDa protein band, while a 16.1 kDa protein band was detected in cells inoculated with the rVP26-Flag (Figure 1A). When a Flag-specific antibody was used for Western blot analysis, a band corresponding to the 16.1 kDa VP26-Flag fusion protein was detected in rVP26-Flag-infected cells, but not in rE1-infected cells (Figure 1A). The control viral protein UL19 was detected in both infected cells, and the cellular control protein GAPDH was detected in all cell samples (Figure 1A). VP26-Flag fusion protein was also detected in purified rVP26-Flag virions (Figure 1B). These results confirm that the Flag tag was successfully fused to VP26 in rVP26-Flag.

### 2.2. Identification of the Interaction Between DEV VP26 and the Host Protein

We performed immunoprecipitation (IP) with rVP26-Flag-inoculated CEF cells using a Flag-specific antibody to capture the VP26-bound host proteins. Subsequent liquid chromatography–tandem mass spectrometry (LC-MS/MS) analysis of the immunoprecipitated proteins identified 7861 peptides, corresponding to 1364 proteins. We then compared the expression levels of these putative VP26-interacting host proteins between rVP26-Flag-infected and uninfected CEF cells. This analysis revealed 17 proteins with a >5-fold difference in expression (*p* < 0.05) between the two groups, which were designated as VP26-associated host proteins (Table 1).

### 2.3. Functional Characterization of VP26-Associated Host Proteins

**Figure 2:**
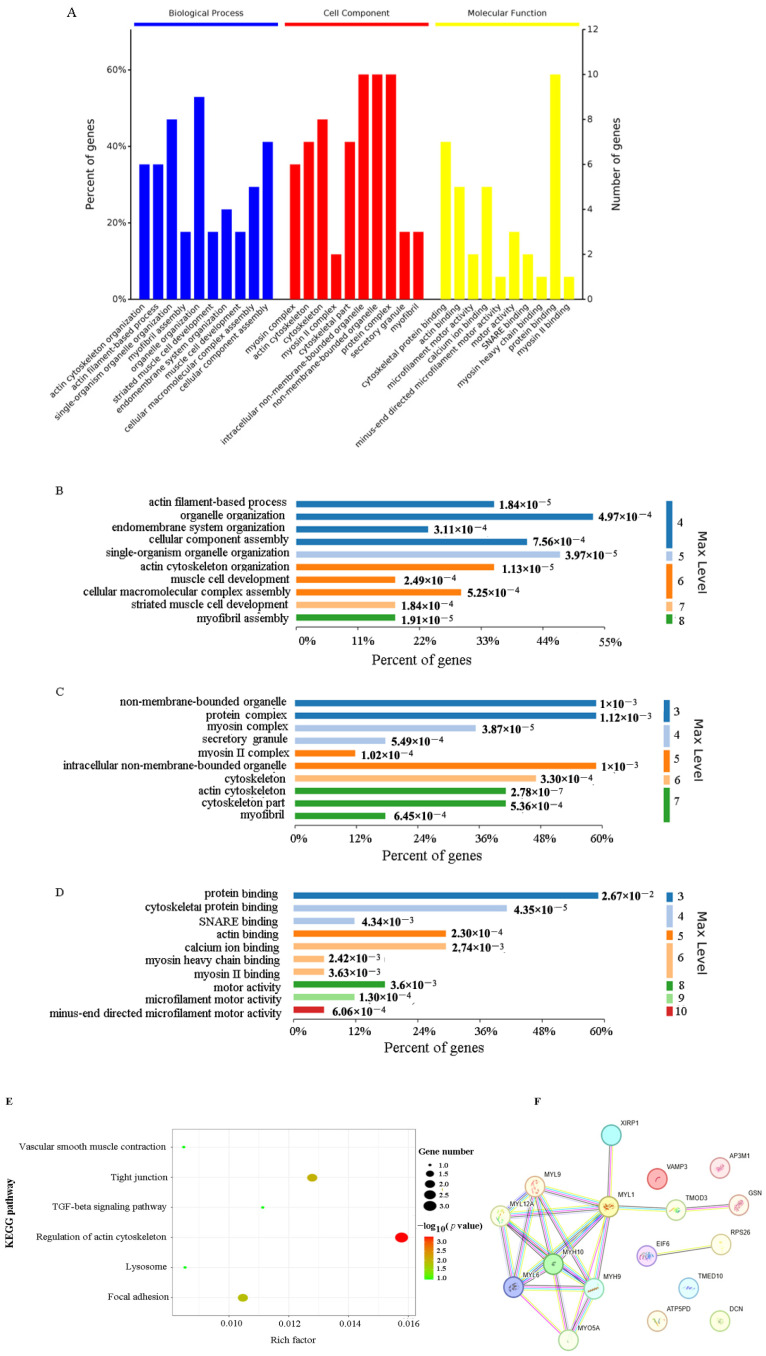
Functional enrichment analysis of VP26-associated host protein genes. (**A**) GO analysis of the identified VP26-associated proteins. A *p*-value < 0.05 is considered statistically significant. VP26-associated host proteins were stratified into 3 functional groups: biological process (BP), cellular component (CC), and molecular function (MF). Shown are the top 10 BPs, CCs, or MFs enriched with VP26-associated host proteins. (**B**–**D**) Top 10 BPs, CCs, and MFs. (**E**) KEGG network analysis of VP26-associated host protein genes. Spot size denotes the gene number, while spot color denotes the *p*-value. *p*-value < 0.01 was set as the significance threshold. (**F**) The identified VP26 interacting proteins were mapped into a protein–protein interaction (PPI) network using the STRING program. The relationship between protein interactions was obtained by choosing targets with a combined score > 0.4. Line colors denote categories based on interaction predictions: gene fusion (red), neighborhood (green), co-occurrence across genomes (blue), co-expression (black), experimental (purple), interaction between curated databases (light blue-lake green), or co-mentioned in PubMed abstracts/text mining (yellow).

Based on Gene Ontology (GO) analysis, VP26-associated host proteins were significantly enriched in biological processes (BPs) such as organelle organization, single-organism organelle organization, cellular component assembly, and actin cytoskeletal organization (Figure 2A,B). In cellular component (CC) categories, enrichment was observed in non-membrane-bound organelles, protein complexes, intracellular non-membrane-bound organelles, the cytoskeleton, actin cytoskeleton, and cytoskeletal parts (Figure 2A,C). Molecular function (MF) annotations included protein binding, cytoskeletal protein binding, actin binding, calcium ion binding, motor activity, SNARE binding, microfilament motor activity, myosin heavy chain binding, myosin II binding, and minus-end directed microfilament motor activity (Figure 2A,D). Kyoto Encyclopedia of Genes and Genomes (KEGG) analysis further revealed enrichment in actin cytoskeletal modulation pathways (Figure 2E).

STRING analysis showed that most VP26-interacting proteins formed a functionally interconnected network of modulatory and actin-interacting proteins, including the cytoskeletal proteins GSN, TMOD3, MYH9, MYH10, MYO5A, and XIRP1 (Figure 2F, Supplementary Material File S3).

### 2.4. Verification of the Interaction Between DEV VP26 and MYH9 in CEF Cells

**Figure 3:**
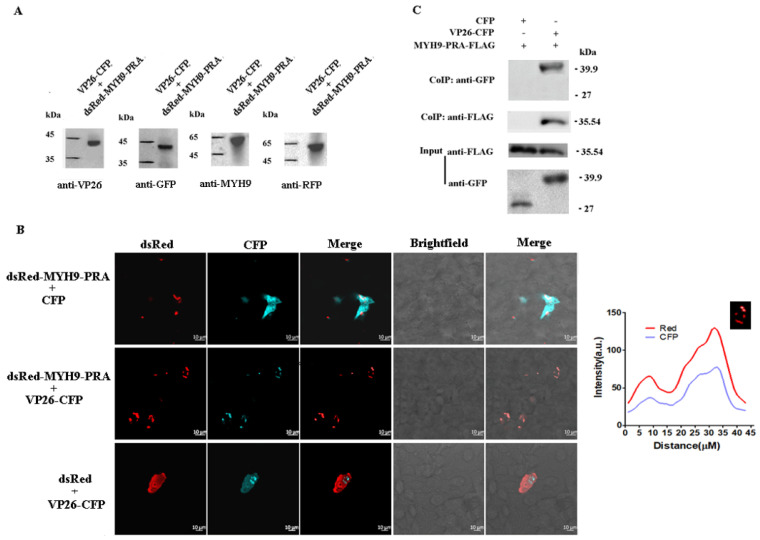
Interaction analysis between DEV VP26 and host MYH9 in LMH cells. The pCMV-UL35-CFP and pdsRed-MYH9-PRA plasmids were co-transfected into LMH cells. (**A**) Confirmation of dsRed-MYH9-PRA and VP26-CFP expression via Western blot analysis. (**B**) Co-localization of dsRed-MYH9-PRA and VP26-CFP via confocal microscopy. The quantitative co-localization analysis was performed with Image J1.42 software. (**C**) The protein–protein interaction between VP26 and MYH9-PRA determined via Co-IP analysis.

Due to MYH9 being a large protein with a molecular mass of up to 226.5 kDa, it is difficult for it to be expressed in full length in vitro. Here, a carboxyl-terminus domain of MYH9 (1651-1960 aa, MYH9-PRA) was chosen to confirm the interaction between VP26 and MYH9 according to the literature [12,13,14,15]. Then, we performed co-localization and Co-IP assays. CEF cells were co-transfected with the plasmids pCMV-UL35-CFP and pdsRed-MYH9-PRA, which express VP26-CFP and dsRed-MYH9-PRA, respectively. The expression of these fusion proteins was verified via Western blot analysis (Figure 3A). Confocal microscopy revealed that VP26-CFP co-localized with dsRed-MYH9-PRA in cells co-transfected with the plasmids (Figure 3B). This co-localization was absent in the control groups, in which the plasmids of CFP control and pdsRed-MYH9-PRA, or dsRed control and pCMV-UL35-CFP, were co-transfected (Figure 3B). Consistently, Co-IP assays confirmed the physical interaction between MYH9 and VP26 (Figure 3C).

### 2.5. Effects of Drugs on Microfilament Cytoskeleton

Compared to control cells, CEF cells treated with 62.5 nM cytochalasin D (Cyto D) exhibited more particulate structures, which are indicative of depolymerized myofilaments.

Treatment with 59.37 nM latrunculin A (Lat A) also induced particulate formation and disrupted the orderly alignment of actin filaments. In contrast, 5 μM (-)-blebbistatin (BLEB) had a minimal effect on myofilament organization. Higher concentrations of the above drugs caused much more severe cellular damage (Supplementary Figure S2).

### 2.6. Effect of Drugs on Viral Infection

**Figure 4:**
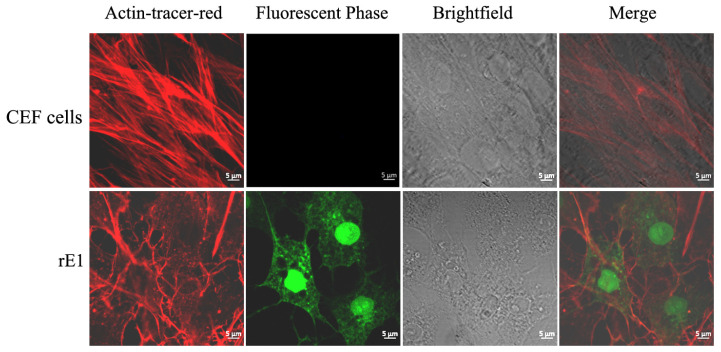
Microfilament rearrangement in rE1-infected cells. rE1-infected CEF cells and negative control CEF cells were stained with Actin–Tracker Red–Rhodamine and visualized under a confocal microscope. CEF cells: control cells; rE1: rE1-infected cells.

Tracking F-actin in rE1-infected cells revealed that DEV infection induces a rearrangement of the host’s cytoskeletal microfilaments. In uninfected cells, actin filaments were organized in parallel lines traversing from one end to the other of a cell. Upon viral inoculation at 36 h, the cells changed shape from an elongated (spindle-like) one to one that was spread out, and the actin filaments formed a network, rather than a spindle, as before (Figure 4).

**Figure 5:**
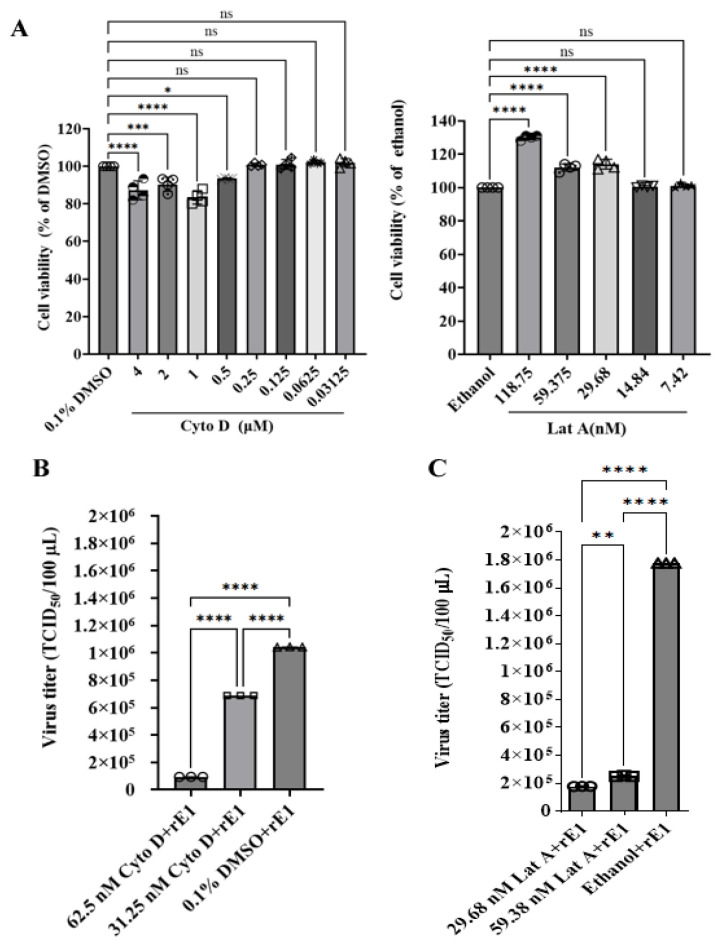
Effect of cytochalasin D (Cyto D) and latrunculin A (Lat A) on viral titers in CEF cells. (**A**) Cytotoxicity of Cyto D and Lat A on CEF cells. CEF cells were incubated with Cyto D or Lat A at the indicated concentrations for 2 h, and cell viability was assessed via a CCK8 assay. The control group was treated with 0.1% DMSO or ethanol. Cell viability was normalized to the control and presented as relative values (*n* = 4 per group). The results are presented as the mean ± S.D. Statistical significance was analyzed using a one-way ANOVA (nonparametric or mixed) (* *p* < 0.05; ** *p* < 0.01; *** *p* < 0.001; **** *p* < 0.0001; ns, not significant). (**B**,**C**) Cyto D and Lat A significantly reduced rE1 titers in CEF cells. Confluent CEF cells in 12-well plates were inoculated with rE1 at an MOI of 0.05. At 6 h post-infection, cells were treated with 62.5 nM/31.25 nM Cyto D, 59.38 nM/29.68 nM Lat A, or control (0.1% DMSO/ethanol) for 17 h. The medium was then replaced with DMEM containing 2% FBS. Viral titers were measured using a TCID_50_ assay at 48 or 72 h post-infection (*n* = 3 per group). Data is presented as the mean ± S.D. of triplicates, with statistical significance analyzed using a one-way ANOVA (nonparametric or mixed) (* *p* < 0.05; ** *p* < 0.01; *** *p* < 0.001; **** *p* < 0.0001; ns, not significant).

To investigate the impact of actin cytoskeleton disruption on viral growth, CEF cells were infected with rE1 for 6 h and subsequently treated with actin polymerization inhibitors (Cyto D or Lat A). Drug concentrations were determined based on cell viability assays (Figure 5A). Our data revealed that rE1 titers were significantly reduced in Cyto D-treated cells compared to 0.1% DMSO-treated cells. Specifically, treatment with 62.5 nM and 31.25 nM Cyto D reduced rE1 titers by 90.93% (*p* < 0.0001) and 34.00% (*p* < 0.0001), respectively (Figure 5B). Similarly, treatment with 59.38 nM and 29.68 nM Lat A decreased rE1 titers by 85.88% (*p* < 0.0001) and 90.00 % (*p* < 0.0001), respectively, compared to 0.1% ethanol controls (Figure 5C). Collectively, these results demonstrate that pharmacological disruption of the microfilament network significantly impairs DEV replication in cells.

### 2.7. The Deficiency of MYH9 Impairs Virus Replication in LMH Cells

**Figure 6:**
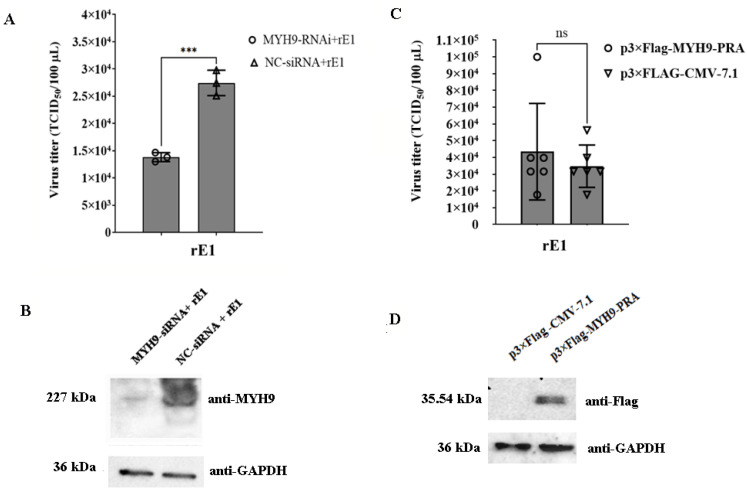
*MYH9* silencing reduces rE1 infection and *MYH9* overexpression enhances rE1 infection. (**A**) siRNA-mediated *MYH9* silencing inhibits rE1 replication. LMH cells (2 × 10^5^ cells/well) were plated in 12-well plates and transfected with control or gene-specific siRNAs (20 nM) for 24 h. Cells were then inoculated with rE1 (MOI = 0.25) for 48 h, and virus titers were determined via TCID_50_ assay (*n* = 3 per group). Data are presented as the mean ± S.D. Statistical significance was analyzed using an unpaired two-tailed Student’s t-test (*** *p* < 0.001; ns, not significant). (**B**) Western blot validation of *MYH9* silencing. Western blot analysis was performed to detect MYH9 protein levels in LMH cells transfected with *MYH9*-specific siRNA. (**C**) MYH9 overexpression promotes rE1 infection. LMH cells (2 × 10^5^ cells/well) were plated in 12-well plates and transfected with p3×Flag-MYH9-PRA and empty vector p3×Flag-CMV-7.1 for 24 h. Cells were inoculated with rE1 (MOI = 0.25) for 48 h, and viral titers were measured via TCID_50_ assay (*n* = 6 per group). Data are presented as the mean ± S.D. Statistical significance was analyzed using an unpaired two-tailed Student’s t-test (ns, not significant). (**D**) Western blot validation of MYH9 overexpression. Western blot analysis was performed to detect MYH9-PRA protein levels in LMH cells transfected with p3×Flag-MYH9-PRA.

To determine the role of MYH9 in viral replication, LMH cells were transfected with a MYH9-specific siRNA duplex; 24 h post-transfection, cells were inoculated with rE1 at an MOI of 0.25, and viral titers were measured 48 h post-inoculation. MYH9 knockdown significantly reduced rE1 titers by 49.66% (*p* < 0.001) compared to control cells (Figure 6A). Western blot analysis confirmed efficient MYH9 protein depletion following siRNA transfection (Figure 6B).

The titers of rE1 in LMH cells transfected with p3×Flag-MYH9-PRA were 24.89% higher than those in cells transfected with the control plasmid p3×Flag-CMV-7.1 (*p* > 0.05) (Figure 6C). These results demonstrate that overexpression of MYH9-PRA enhanced rE1 replication in LMH cells. Western blot analysis confirmed the expression of MYH9-PRA protein in LMH cells (Figure 6D).

### 2.8. (-)-Blebbistatin Inhibited DEV Replication In Vitro and In Vivo

**Figure 7:**
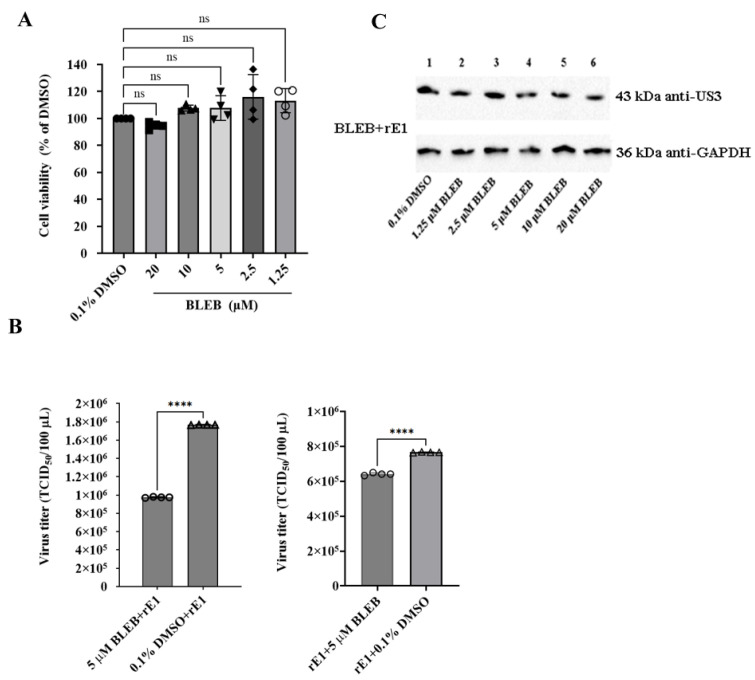
Effect of (-)-Blebbistatin (BLEB) on virus titers in CEF cells. (**A**) Cytotoxicity of BLEB on CEF cells. CEF cells were incubated with BLEB at the indicated concentrations for 2 h, and cell viability was determined using a CCK8 assay. The control group was treated with 0.1% DMSO (*n* = 4 per group). Cell viability was expressed as a percentage relative to the control. The results are presented as the mean ± S.D. Statistical significance was analyzed using a one-way ANOVA (nonparametric or mixed) (ns, not significant). (**B**) BLEB significantly reduces rE1 titers in CEF cells. Confluent CEF cells in 12-well plates were inoculated with rE1 at an MOI of 0.05. At 6 h post-inoculation, cells were treated with 5 μM BLEB or 0.1% DMSO (control) for 17 h. The medium was then replaced with DMEM without FBS, and samples (cells resuspended in culture medium) were harvested at 48 h post-infection. Alternatively, CEF cells were pre-treated with 5 μM BLEB or 0.1% DMSO for 6 h, before inoculation with rE1 (MOI = 0.05). Viral titers were measured using a TCID_50_ assay at 48 or 72 h post-infection (*n* = 4 per group). Data are presented as the mean ± S.D. Statistical significance was analyzed using an unpaired two-tailed Student’s t-test (**** *p* < 0.0001). (**C**) The effect of BLEB on viral protein US3 expression. Viral protein US3 was detected via Western blot analysis, with GAPDH used as an internal control.

(-)-Blebbistatin (BLEB), a specific suppressor of myosin II ATPase activity [16,17], was used to further investigate the role of MYH9 in DEV replication in host cells. The concentration of BLEB was first determined using a cell viability assay (Figure 7A). Two independent experiments were designed to investigate the effect of BLEB on DEV replication in CEF cells: (1) cells were first infected with the virus and then treated with 5 μM BLEB, and (2) cells were initially treated with 5 μM BLEB and then infected with the virus. As determined using a TCID_50_ assay, the titer of BLEB-treated rE1 in experiment (1) was 16.28% (*p* < 0.0001) lower than that of DMSO-treated rE1. The titers of rE1 from BLEB-treated cells in experiment (2) were 44.82% (*p* < 0.0001) lower than those of DMSO-treated cells (Figure 7B). These findings indicate that the BLEB treatment partially suppressed viral replication in CEF cells. However, BELB treatment had a minimal impact on DEV US3 expression in cells, demonstrating that BLEB has no effect on the expression of some virus proteins (Figure 7C).

**Figure 8:**
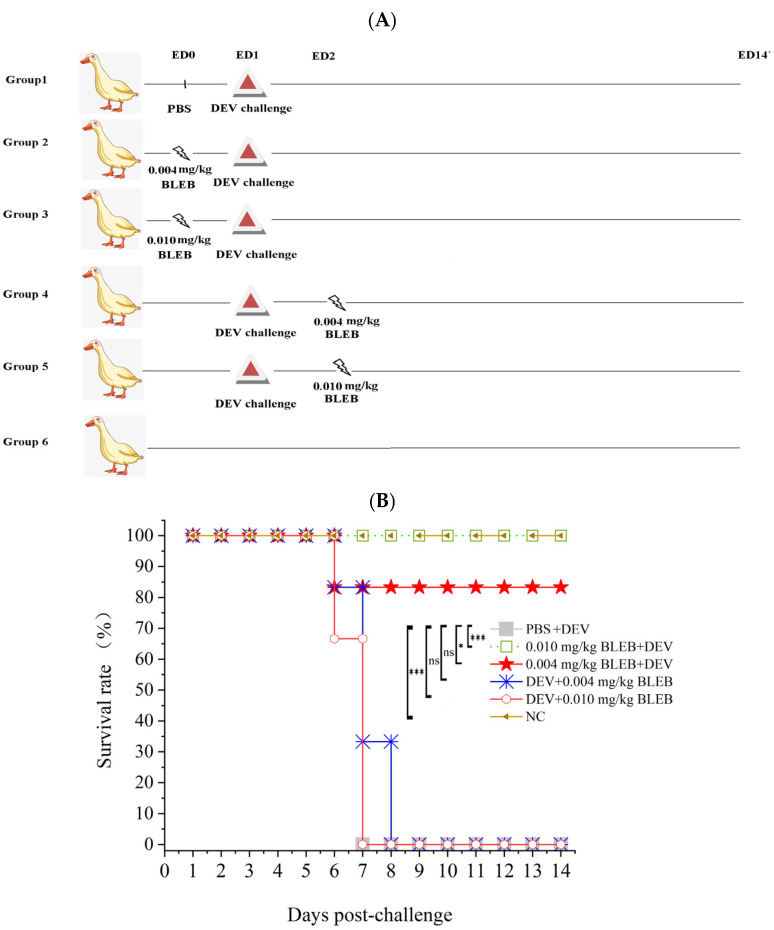
BLEB inhibits DEV replication in vivo. (**A**) Schematic of animal experiments. Ducks in Groups 1, 2, and 3 were intranasally (i.n.) administered PBS, 0.004 mg/kg BLEB, and 0.010 mg/kg BLEB on experimental day 0 (ED0). On ED1, Groups 1, 2, 3, 4, and 5 were intramuscularly (i.m.) administered a highly virulent DEV CHv strain at a dose of 100-fold the 50% duck lethal dose (DLD_50_). Additionally, on ED2, Groups 4 and 5 were provided intranasally (i.n.) with BLEB at a dose of 0.004 mg/kg and 0.010 mg/kg, respectively. Ducks in Group 6 were left untreated and served as the normal control group. Disease symptoms and mortality of each group were monitored for 2 weeks. (**B**) Survival curves of the ducks. Statistical significance was determined using a simple survival analysis (Kaplan–Meier) (* *p* < 0.05; *** *p* < 0.001; ns, not significant).

To investigate the role of MYH9 in DEV pathogenesis in vivo, we conducted a duck infection study following the experimental schema outlined in Figure 8A. Two-month-old female ducks were intranasally (i.n.) administered PBS or BLEB at 0.004 mg/kg or 0.010 mg/kg either pre-infection (24 h prior) or post-infection (6 h after) with the virulent CHv strain of DEV. Clinical morbidity and mortality were monitored over 14 days. The results are demonstrated in Figure 8B and Table 2. Collectively, these data indicate that the ATPase activity of myosin II is crucial for DEV replication both in vitro and in vivo.

## 3. Discussion

In addition to infection in ducks, it is noteworthy that cases of chickens infected with DEV have also been documented [18]. As documented in the literature [19,20,21,22] and our previous study [23], inoculation of CEF cells with the highly virulent DEV strain or the chicken embryo-attenuated vaccine strain elicits cytopathic effects and leads to chick mortality. The chicken embryo-attenuated DEV has been used as a classical live vaccine since its development by Chinese scientists in 1964 and has effectively controlled DEV outbreaks. However, this vaccine strain presents a safety hazard for chickens, potentially causing mortality upon inoculation. When chicken and duck flocks are co-raised, the DEV vaccine strain may induce infection in chicken flocks. Thus, this study provides insights for the further attenuation of the DEV vaccine strain, which is a good candidate for a live viral vector [23,24,25,26,27].

In this study, chick cells were used as the in vitro model due to two key constraints: the lack of accessible passaged duck cells and constraints on SPF duck-derived resources. Although the functional relevance of chicken host proteins interacting with VP26 may be limited when extrapolated to ducks, it is notable that the amino acid homology of all putative VP26-interacting proteins (GSN, TMOD3, MYH9, TMED10, MYH10, DCN, EIF6, VAMP3, ATP5PD, AP3M1, MYO5A, and RSP26) between chickens and ducks is remarkably high (Table 1). Among these proteins, MYH9, MYH10, MYO5A, EIF6, AP3M1, and PRS26 exceed 98% homology (see Supplementary Material File S4). Therefore, data obtained from cells originating from chicks have a significant reference value for duck-related studies.

A comprehensive understanding of host–pathogen interactions is essential for deciphering viral life cycles, unraveling the mechanisms of viral pathogenesis, and identifying potential antiviral therapeutic targets [28,29,30,31]. Protein–protein interactions represent the primary regulatory nodes in host–pathogen biology. We employed a Flag-tagged recombinant DEV to identify host proteins interacting with VP26 for IP and subjected the purified proteins to large-scale proteomics analysis. The subsequent molecular function annotation, STRING analysis, revealed that the enriched proteins formed a PPI network predominantly associated with the actin filament-based cytoskeleton. Actin filaments are essential for cellular dynamics, mediating functions such as cell integrity maintenance, muscle contraction, stress fiber formation, cellular protrusions (filopodia/lamellipodia), and perinuclear/cortical organization [32]. The actin–myosin network is highly concentrated beneath the plasma membrane, with cytoskeletal actin filaments and associated proteins playing critical roles in the viral life cycle [32,33,34,35,36,37,38,39,40,41,42,43,44]. The actin cytoskeleton and Rho GTPase signaling to actin assembly are prime targets of bacterial and viral pathogens [45]. Non-muscular myosins are crucial for viral transport along F-actin- or myosin-driven actin rearrangement for viral entry/egress [46,47,48,49,50,51,52]. Emerging evidence shows herpesviruses co-opt actin and myosin motors for entry, intranuclear capsid transport, and virion egress. For instance, HSV-1 gB and pUL49 (VP22) separately interact with myosin and mediate HSV-1 entry and egress [48,49]; Non-muscle Myosin Heavy Chain-IIB mediates HSV-1 entry into corneal nerves [50]; MYH9 is a new mediator of porcine reproductive and respiratory syndrome virus (PRRSV) entry [12]; MYH PRV VP26-GFP co-localizes with myosin V in infected cells [11]; Myosin Va is required for efficient Oropouche virus cell egress [46]; and MYH IIA promotes the internalization of influenza A virus and regulates viral polymerase activity [52]. This study initially characterized the DEV VP26-interacting avian proteome, with STRING analysis indicating that most of the identified proteins form a functionally interconnected network of actin-modulatory proteins.

Cyto D and Lat A disrupt actin filaments through distinct mechanisms: the former binds to the ends of developing actin filaments, preventing them from assembling and initiating depolymerization [53,54], while the latter promotes actin filament disintegration by preventing actin monomers from adhering to a developing actin filament. In addition, it could accelerate actin filament depolymerization [55,56]. In this study, both of these drugs significantly reduced viral titers in rE1-infected CEF cells, demonstrating that DEV replication relies on normal actin filament dynamics. We further validated the interaction between VP26 and MYH9 using co-localization and Co-IP assays and proved that the MYH9 carboxyl-terminus domain directly associates with DEV VP26. This finding aligns with reports on PRRSV, murine gammaherpesvirus 68 (MHV-68), and SARS-CoV-2, whose viral proteins (e.g., PRRSV GP5, MHV-68 gp150, SARS-CoV-2 S) also interact with the C-terminus of MYH9 [12,13,14,15]. In this study, the siRNA-mediated MYH9 knockdown reduced virus titers, and (-)-Blebbistatin, an inhibitor of non-muscle myosin (NM-II) ATPase activity, impaired viral proliferation both in vitro and in vivo. The in vitro experiment demonstrated that treatment with BLEB had a minimal impact on DEV US3 expression, demonstrating that BLEB does not affect viral proliferation by interfering with viral protein expression. Instead, it might be attributed to a disruption of later stages of the DEV life cycle, especially processes dependent on the actin–myosin II network, such as intracellular viral transport and viral egress from infected cells. In the duck infection model, prophylactic BLEB administration (0.010 mg/kg) conferred complete protection against lethal DEV challenges, whereas post-infection treatment was ineffective. These data show that MYH9 ATPase activity is essential for viral entry and early replication steps, or viral spread. As a highly pathogenic, systemic virus, DEV exhibits an extremely rapid rate of dissemination in hosts [57,58,59,60]. Consistent with this, BLEB administration before viral infection effectively inhibited DEV; in contrast, BLEB treatment initiated 1 day after infection failed to exert meaningful antiviral effects. This is likely because DEV had already established widespread systemic replication by that time, making the intervention too late to disrupt its propagation. Collectively, our findings identify MYH9 as a critical host factor for DEV, positioning it as a promising target for antiviral therapeutic development.

## 4. Materials and Methods

### 4.1. Virus Strain, Cells, and Plasmids

The bacterial pDEV-EF1/GS1783 harboring the entire infectious bacterial artificial chromosome (BAC) clone of DEV pE1 and the associated GFP-linked virus rE1 were constructed and maintained in our laboratory at Zhejiang Academy of Agricultural Sciences (ZAAS) [61,62]. The highly virulent CHv strain (CVCC AV1221) was acquired from the China Institute of Veterinary Drugs Control (Beijing, China). Specific-pathogen-free (SPF) chicken embryos were obtained from Zhejiang Jianliang Bioengineering Co., Ltd. (Hangzhou, China) CEF cells were isolated from 9- to 11-day-old SPF embryos following a previously described protocol [61]. CEF cells were grown in Dulbecco’s Modified Eagle Medium (DMEM; Gibco BRL, Grand Island, NY, USA) supplemented with 10% fetal bovine serum (FBS; Gibco BRL, Grand Island, NY, USA), 100 U/mL penicillin (Sangon, Shanghai, China), and 100 μg/mL streptomycin (Sangon, Shanghai, China). The LMH chicken hepatocellular carcinoma cell line was maintained in DMEM/F12 medium (Gibco BRL, Grand Island, NY, USA) with identical supplements. We obtained the pdsRed2-C1 vector from Biovector NTCC Inc. (Beijing, China), the pCMV-C-CFP vector from Beyotime (Shanghai, China), and the p3×Flag-CMV-7.1 vector from Wuhan GeneCreate Biological Engineering Co., Ltd. (Wuhan, China). The recombinant plasmid pCMV-UL35-CFP was constructed by cloning the VP26-encoding gene UL35 into the pCMV-C-CFP vector using the primers UL35-CFP(BamHI+) and UL35-CFP(EcoRI-). The recombinant plasmid p3×Flag-MYH9-PRA was constructed by inserting the synthesized MYH9-PRA fragment (a carboxyl-terminal truncated form of MYH9) into the p3×Flag-CMV-7.1 vector. Additionally, pdsRed-MYH9-PRA was generated by cloning the same MYH9-PRA gene into the pdsRed2-C1 vector (Clontech, Mountain View, CA, USA) using the primers dsRed-myh9-PRA(SalI+) and dsRed-myh9-PRA(BamHI-). All primers used in this study are summarized in Table 3.

### 4.2. Inhibitors

The following reagents were used to disrupt the microfilaments or inhibit myosin activity: cytochalasin D (Sigma-Aldrich, St. Louis, MO, USA), latrunculin A (APExBIO, Houston, TX, USA), and (-)-Blebbistatin (Beyotime, Shanghai, China).

### 4.3. Reverse Genetics System

**Figure 9:**
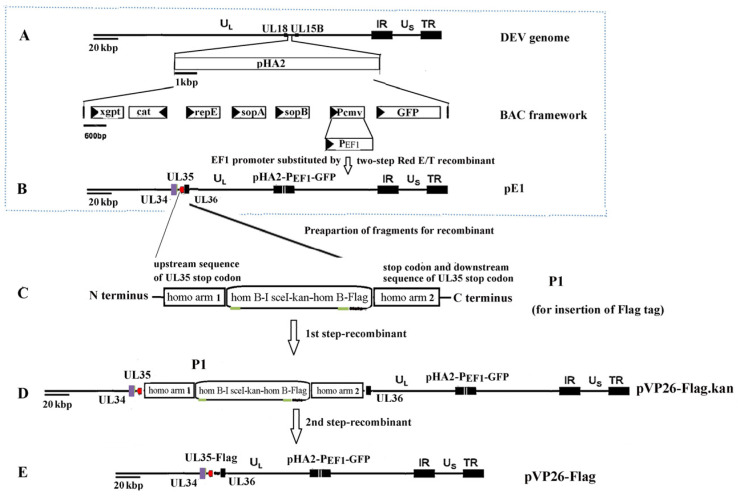
Construction of pVP26-Flag. (**A**) A mini-F vector (pHA2), as a BAC that allows the maintenance of large circular DNA in E. coli, was introduced into the intergenic region between UL15B and UL18 of a DEV vaccine strain via homologous recombination; (**B**) the pCMV promoter, which controls the expression of GFP in pDEV-vac, was substituted by the pEF1 gene via a two-step Red-mediated recombination (*en passant*); (**C**–**E**) insertion of Flag-tag to the carboxyl terminus of UL35 via a two-step Red-mediated recombination (*en passant*). The constructs shown in the dotted box (A and B) were prepared in our previous study [30].

The pVP26-Flag, with a Flag tag fused to the carboxyl-terminus of the VP26 gene, was constructed using pE1 as the backbone vector [31] via a two-step Red (*en passant*) recombination strategy [63,64] (Figure 9). The PCR fragment P1 carrying homo-arm1 (homologous to the upstream sequence of the UL35 stop codon)-hom B-I-SceI-kan-hom B-Flag tag-homo-arm2 (homologous to the UL35 stop codon and downstream sequence) was synthesized by Tsingke Biotech. Co., Ltd. (Beijing, China) and used to generate pVP26-Flag. pVP26-Flag was confirmed via sequencing. rVP26-Flag was rescued by transfecting pVP26-Flag into CEF cells using the calcium phosphate precipitation procedure described by Chen et al. [61].

### 4.4. Virion Purification

rVP26-Flag-infected CEF cells and supernatants were collected at 48 h. rVP26-Flag was purified via sucrose density gradient ultracentrifugation, as described in Zhao et al. [65].

### 4.5. Western Blot Analysis

Protein samples were separated in 15% or 12% SDS-PAGE and transferred to a nitrocellulose membrane (NC; Merck Millipore, Billerica, MA, USA). Membranes were blocked overnight at 4 °C with 10% skim milk in PBST (PBS containing 0.5% Tween-20) to minimize non-specific binding, followed by incubation for 1 h at 37 °C with the target protein-specific antibodies and horseradish peroxidase (HRP)-conjugated goat anti-rabbit or anti-mouse IgG (Santa Cruz Biotechnology, Santa Cruz, CA, USA). Protein bands were visualized using SuperSignal West Pico Chemiluminescent Substrate (Pierce, Rockford, IL, USA) with a CCD camera-equipped ChemiDocTM XRS+ Imaging System (Bio-RAD, Hercules, CA, USA). The following primary antibodies were used in Western blot analysis: anti-VP26 rabbit polyclonal antibody (1:250; Genscript Co., Nanjing, China), anti-US3 mouse polyclonal antibody (1:500; Genscript Co., Nanjing, China), non-muscle myosin IIA antibody (1:1000; Novus Biologicals, Littleton, CO, USA), GFP monoclonal antibody (also cross-reactive with CFP; 1:1000; Beyotime, Shanghai, China), Flag monoclonal antibody (1:1000; Beyotime, Shanghai, China), and RFP polyclonal antibody (1:1000; Abcam, Cambridge, MA, USA).

### 4.6. Indirect Immunofluorescence (IF)

CEF cells were seeded on coverslips and infected with rE1 at an MOI of 0.05. After 36 h, F-actin was stained with Actin–Tracker Red–Rhodamine (Beyotime, Shanghai, China), and images were captured using a Leica TCS-SP5 laser scanning confocal microscope (Leica, Heidelberg, Germany) with a 100 × oil immersion objective.

### 4.7. Co-Immunoprecipitation (Co-IP)

Co-IP was performed using a Pierce Co-IP Kit (catalog no. #26149, ThermoFisher, Waltham, MA, USA) following the manufacturer’s instructions. To identify proteins interacting with DEV VP26, CEF cells were infected with rVP26-Flag and cultured until ~90% of cells expressed the Flag-tagged protein. Cells were lysed in IP lysis buffer (25 mM Tris-HCl pH 8.0, 200 mM NaCl, 5 mM MgCl2, 1 mM DTT), and protein concentrations were determined using a Pierce BCA Protein Assay Kit (ThermoFisher, Waltham, MA, USA). For immunoprecipitation, 1 mg of total protein was incubated overnight at 4 °C with rabbit IgG- or anti-Flag antibody-conjugated resins (Beyotime, Shanghai, China). Resins were washed three times with IP lysis buffer before being subjected to protein digestion and nano LC-MS/MS analysis.

To validate the interaction between VP26 and MYH9, pCMV-UL35-CFP or pCMV-C-CFP (as a control) and p3×Flag-MYH9-PRA were co-transfected into LMH cells on 6-well plates using the Exfect transfection reagent (Vazyme, Nanjing, China). Then, 48 h post-transfection, the cells were lysed and Co-IP was performed. VP26-CFP and MYH9-PRA-Flag proteins were immunoprecipitated with an anti-GFP monoclonal antibody (1:1000; Beyotime, Shanghai, China) and an anti-Flag monoclonal antibody (1:1000; Beyotime, Shanghai, China), respectively.

### 4.8. LC-MS/MS Analysis

Proteins co-immunoprecipitated with VP26-Flag were digested with trypsin to generate peptides for nano LC-MS/MS analysis. Briefly, proteins co-immunoprecipitated with the VP26-Flag binding resins were dissolved in 100 μL 50 mM NH_4_HCO_3_ (pH 8.0) and then incubated with 10 mM dithiothreitol for 1 h at 56 °C to initiate reduction. Alkylation was performed by incubating the reduced proteins with 50 mM iodoacetamide (IAA) for 40 min at room temperature in the dark. Trypsin-mediated digestion was carried out by adding trypsin to the reduced and alkylated proteins at a 1:100 (*w*/*w*) trypsin/protein ratio, followed by an overnight incubation at 37 °C. The trypsin digestion was stopped by adding 10 μL of 0.1% formic acid. The peptide solution from the trypsin digestion then underwent desalting in a self-priming desalting column, followed by vacuum drying. The dried peptides were resuspended with solution A (0.1% formic acid, 2% acetonitrile), followed by centrifugation at 16,200× *g* for 10 min at 4 °C. The resulting supernatant was used for MS analysis.

MS analysis was performed using an Ultimate 3000 ultra-high performance liquid chromatography system (ThermoFisher, Waltham, MA, USA) and a Q Exactive™ Hybrid Quadrupole-Orbitrap™ MS apparatus (ThermoFisher, Waltham, MA, USA) with an ESI nanospray source (2.2 kV spray voltage and 270 °C capillary temperature). Peptide separation was performed using a nanocolumn (100 μm × 10 cm in-house-made column) filled with a reverse-phase ReproSil-Pur C18-AQ resin (3 μm, 120 Å, Dr. Maisch GmbH, Ammerbuch, Germany). The following MS/MS parameters were used: production scan range, *m*/*z* 100; activation type, CID; min. signal required, 1500.0; isolation width, 3.00; normalized collision energy, 40.0; default charge state, 6; activation Q, 0.250; and activation time, 30.000.

### 4.9. Quantification and Bioinformatics Analysis

Raw MS data were assessed against the UniProt Gallus gallus database using MaxQuant (version 1.6.2.10). The MaxQuant screening parameters were as follows: cysteine (C) carbamidomethylation was defined as fixed, methionine (M) oxidation was defined as variable, N-terminal acetylation was defined as variable, the enzyme specificity was adjusted to trypsin, the maximum missed cleavage was adjusted to 2, the precursor ion mass tolerance was adjusted to 20 ppm, and the MS/MS tolerance was adjusted to 20 ppm. Only high-confidence peptides (1% FDR) were selected for downstream protein recognition analysis.

Functional enrichment analyses of associated proteins were performed using GO and KEGG network enrichment analyses using the OmicsBean Group Data Integration Analysis Cloud Platform (https://www.omicsbean.cn/). The PPI axis of VP26-associated key proteins was evaluated using the web-based program STRING (https://string-db.org). The relationship between protein interactions was obtained by choosing targets with a combined score > 0.4. Cytoscape 3.10.3 (https://cytoscape.org/) was used for visualization.

### 4.10. Immunofluorescence and Confocal Microscopy

To assess the co-localization of VP26 and MYH9-PRA, pCMV-UL35-CFP and pdsRed-MYH9-PRA, pCMV-UL35-CFP and pdsRed2-C1, or pCMV-C-CFP and pdsRed-MYH9-PRA were co-transfected into 90% confluent LMH cells using Exfect transfection Reagent (Vazyme, Nanjing, China). Protein expression from the transfected plasmids was confirmed using Western blotting. Thirty-six hours post-transfection, cells were imaged using a Leica TCS-SP5 laser scanning confocal microscope (Leica, Heidelberg, Germany) with a 100 × oil immersion objective. Co-localization was quantified using NIH Image J1.42 software.

### 4.11. Cell Viability Assay

To evaluate the cytotoxicity of Cyto D, Lat A, and BLEB, CEF cells (1 × 10^4^ cells/well) were seeded in 96-well plates 24 h prior to treatment. Inhibitors were serially diluted in dimethyl sulfoxide (DMSO) or ethanol and added to the cultures. After 24 h, cell viability was assessed using the Cell Counting Kit-8 (CCK-8; Vazyme, Nanjing, China) according to the manufacturer’s protocol. Optical density (OD) was measured at 450 nm using a SpectraMax M3 microplate reader (Molecular Devices, SanJose, CA USA) 1 and 2 h post-CCK-8 addition. Concentrations and durations of treatment that minimized cell death were selected for subsequent experiments.

### 4.12. Effect of Drugs on Microfilament Cytoskeleton

CEF cells were pretreated with 62.5 nM Cyto D, 59.37 nM Lat A, 5 μM BLEB, or control (DMSO/ethanol) for 17 h; they were then fixed with 4% formaldehyde and stained with Actin–Tracker Red–Rhodamine (Beyotime, Shanghai, China). The microfilament cytoskeleton in the treated cells was visualized using an eclipse TS2 inverted microscope (Nikon, Tokyo Prefecture, Japan) to assess drug-induced cytoskeletal disruption.

### 4.13. Inhibition Assay

Confluent CEF cells in 12-well plates were infected with rE1 at an MOI of 0.05. Six hours post-infection, cells were treated with either Cyto D (62.5 nM, 31.25 nM), Lat A (59.37 nM, 29.68 nM), BLEB (5 μM), or control (0.1% DMSO/ ethanol) for seventeen hours. Inhibitor-containing medium was then replaced with fresh medium, and samples (cells and culture supernatant) were collected at 48 h or 72 h post-infection (based on fluorescence visualization) and stored at −70 °C. In order to assess the effectiveness of BLEB, a separate group was established where pharmacological treatment was administered before virus infection. Confluent CEF cells placed in 12-well plates were pretreated with either 5 μM BLEB or 0.1% DMSO 6 h prior to inoculation with rE1 at an MOI of 0.05. Samples were harvested at 48 h post-infection and lysed using sonication with Tissuelyser-24 (Shanghai Jingxin Technology, Shanghai, China) at 65 Hz for 90 s. Cell lysates were centrifuged at 3500× *g* for 5 min. Next, 100 μL of the supernatant was employed for viral titration using TCID_50_ evaluation based on the classical virological technique. Each sample was evaluated three times in triplicate. Additional cell samples were obtained and treated with BLEB using the same process described above, and these samples were taken for detecting viral protein expression via Western blot analysis.

### 4.14. siRNA-Mediated Gene Silencing

Three small interfering RNAs (siRNAs) targeting distinct regions of the *MYH9* transcript (GenBank: NM_205477.2) were designed using the siDirect 2.0 software and synthesized by GenePharma (Shanghai, China) (Table 1). A nonsense scrambled siRNA served as negative control. LMH cells were transfected with 10 nM siRNA duplexes using Lipofectamine RNAiMAX (ThermoFisher, Waltham, MA, USA), with each transfection performed in triplicate. Cell samples were harvested at 24, 48, and 72 h post-transfection for analysis of MYH9 expression. The *MYH9* mRNA and protein levels were measured using quantitative real-time PCR (qRT-PCR) on the ABI 7500 Real-time PCR system (Applied Biosystems, Waltham, MA, USA) and Western blot analysis, respectively. In the Western blot analysis, MYH9 protein was detected using anti-non-muscle myosin IIA antibody (Novus Biologicals, Littleton, CO, USA), and GAPDH was detected as a control, using mouse monoclonal anti-GAPDH antibody (Beyotime, Shanghai, China). To determine the effect of *MYH9* knockdown on replication of rE1, cells transfected with *MYH9* siRNA were infected with rE1 at an MOI of 0.25. At 48 h post-infection, viral titers and protein levels (MYH9, DEV US3) were analyzed as described above.

### 4.15. Overexpression Analysis

LMH cells (80% confluence) were transfected with either p3×Flag-MYH9-PRA or empty vector p3×Flag-CMV-7.1 using Exfect Transfection Reagent (Vazyme, Nanjing, China) following the manufacturer’s protocol. Twenty-four hours post-transfection, MYH9-PRA expression was confirmed using Western blotting. Transfected cells were then infected with rE1 at an MOI of 0.25, incubated for 48 h, and harvested for viral titer analysis as previously described. Each experiment was performed in triplicate.

### 4.16. Animal Experiments

All animal procedures were performed in accordance with the guidelines approved by the Animal Welfare and Research Ethics Committee of the ZAAS (Approval No.: 2020ZAASLA63, approval date 2 July 2020).

Thirty-six DEV-free healthy female mallards (60 days old) were obtained from a DEV-free farm in Huzhou, Zhejiang, China. Duck group allocation was performed in accordance with the randomization, stratification, and baseline homogeneity principles to eliminate subjective bias and ensure inter-group comparability. Ducks were assigned to six groups (n = 6 per group) and housed in separate rooms in optimal conditions in terms of temperature (28 ± 3 °C), humidity (60 ± 10%), artificial ventilation, and lighting (with a 12/12 light/dark cycle). Ducks in Group 1 received the DEV CHv strain only. Ducks in Groups 2 and 3 were individually provided with 0.004 mg/kg and 0.010 mg/kg BLEB, respectively, 1 day prior to the DEV challenge. Ducks in Groups 4 and 5 received 0.004 mg/kg and 0.010 mg/kg BLEB, respectively, 1 day post the DEV challenge. Ducks in Group 6 were untreated and employed as normal control (NC). Ducks in Group 1 were administered PBS (0.5 mL); ducks in Groups 2 and 3 were intranasally (i.n.) administered 0.004 mg/kg and 0.010 mg/kg of BLEB diluted in 0.5 mL of PBS, respectively, on experiment day 0 (ED0) and experiment day 1 (ED1). Ducks in Groups 1, 2, 3, 4, and 5 were intramuscularly administered a 100-fold 50% duck lethal dose (DLD_50_) of the DEV CHv strain. On ED2, Groups 4 and 5 were provided (i.n.) with 0.004 mg/kg and 0.010 mg/kg of BLEB diluted in 0.5 mL of PBS per duck, respectively. Ducks in Group 6 were untreated. Disease symptoms and mortality were monitored daily for 14 days.

### 4.17. The Control Material and the Control Procedures

Rabbit IgG-conjugated resins (Beyotime, Shanghai, China) were used as the control in Co-IP assays; DMSO (Sigma-Aldrich, St. Louis, MO, USA) or ethanol (Sangon, Shanghai, China) was used as the control reagent in drug blocking analysis; a nonsense scrambled siRNA (GenePharma, Shanghai, China) served as the negative control in the siRNA-mediated gene silencing analysis.

### 4.18. Statistical Analysis

All experiments were independently repeated at least three times (*n* ≥ 3 per group), and data are presented as mean ± standard deviation (SD). Statistical analyses were performed using GraphPad Prism 9.0 (GraphPad Software, San Diego, CA, USA). Statistical significance between two groups was determined using an unpaired two-tailed Student’s t-test. Multiple group comparisons were performed using one-way analysis of variance (ANOVA) or a two-way ANOVA. A *p*-value < 0.05 indicates statistical significance, while *ns* indicates no significant difference. The level of statistical significance is marked with asterisks (* *p* < 0.05, ** *p* < 0.01, *** *p* < 0.001, **** *p* < 0.0001).

## 5. Conclusions

This study systematically characterized the interactome of DEV VP26 with avian host proteins, revealing significant enrichment in actin filament-based cytoskeleton components through STRING analysis. Pharmacological disruption of actin dynamics using cytochalasin D and latrunculin A potently inhibited the viral replication, underscoring the essential role of actin filaments in DEV propagation. Mechanistically, we validated a direct interaction between VP26 and MYH9, a non-muscle myosin II (NM-II) isoform. siRNA-mediated MYH9 knockdown and NM-II ATPase inhibition by (-)-blebbistatin significantly impaired viral titers in vitro and in vivo, with prophylactic BLEB administration completely preventing lethal DEV infection in ducks. Collectively, these findings establish MYH9 as a critical host dependency factor and a promising therapeutic target for DEV, offering novel insights into herpesvirus pathogenesis and potential antiviral strategies.

## Figures and Tables

**Table 1:** Potential VP26-associated host proteins in rVP26-Flag-inoculated CEF cells.

Protein ID	Name	Peptides	Sequence Coverage [%]	Average Intensity of Test	Average Intensity of Ctrl	FC (Test vs. Ctrl)	Amino Acid Sequence Homology Between Duck and Chicken
F1NA76	TMED10	7	40.6	189,220,000	0	Inf	94.06%
F1P4I3	VAMP3	4	53.8	47,205,000	0	Inf	89.42%
Q91957	Xirp1	9	6.3	53,513,333.33	0	Inf	89.00%
Q5ZLY3	TMOD3	7	31.8	141,886,666.7	0	Inf	97.72%
P28675	DCN	5	19.9	48,087,000	0	Inf	97.76%
E1C658	ATP5PD	6	37.3	81,129,333.33	0	Inf	74.19%
Q5ZMP7	AP3M1	5	17	15,092,000	0	Inf	98.80%
P02604	-	8	56.2	407,123,333.3	0	Inf	/
P02612	-	8	62.2	373,010,000	0	Inf	/
Q02440	MYO5A	7	5.3	54,430,333.33	0	Inf	/
Q789A6	MYH10	147	66.8	19,682,333,333	197,000,000	99.91032149	99.00%
A0A1D5PM19	MYH9	173	70	55,490,000,000	1,570,066,667	35.3424483	98.00%
A0A1L1RLN6	-	9	74	2,179,333,333	158,516,666.7	13.74829145	/
P24032	-	10	62.8	2,327,600,000	174,780,000	13.31731319	/
O93510	GSN	24	43.6	1,491,766,667	213,916,666.7	6.973587846	94.68%
Q5ZM66	RPS26	3	29.6	274,823,333.3	41,238,333.33	6.664268682	100%
A0A1D5PNG3	EIF6	2	13.1	35,480,333.33	5,989,000	5.924250014	98.37%

-: protein not fully verified; Inf: infinity.

**Table 2:** Statistics of dead ducks of each group infected with DEV.

	d.p.c	1	2	3	4	5	6	7	8	9	10	11	12	13	14	Survival Rate
Group																
PBS+DEV						6										0/6
0.010 mg/kg BLEB+DEV																6/6
0.004 mg/kg BLEB+DEV					1											5/6
DEV+0.004 mg/kg BLEB					1	3	2									0/6
DEV+0.010 mg/kg BLEB					2	4										0/6

Note: d.p.c: day post challenge with DEV.

**Table 3:** Primers and siRNAs used in this study.

Name	Sequence (5′ to 3′)	Restriction Site Introduced
dsRed-myh9-PRA(SalI+)	gGTCGACatgcgggaactggaggacac	Sal I
dsRed-myh9-PRA(BamHI-)	cgGGATCCttattcagtagctttggcatcacc	Bam HI
UL35-CFP(BamHI+)	cgGGATCCatgtctaattctggaggttc	Bam HI
UL35-CFP(EcoRI-)	cgGAATTCtcgctgatcgtctggcgcg	Eco RI
siRNA-MYH9-1870-s	CUGGCAAGGUGGAUUAUAATT	
siRNA-MYH9-1870-as	UUAUAAUCCACCUUGCCAGTT	
siRNA-MYH9-5503-s	GCCAGAACAAGGAGCUUAATT	
siRNA-MYH9-5503-as	UUAAGCUCCUUGUUCUGGCTT	
siRNA-MYH9-2693-s	GGCCAAGGAAGAAGAACUATT	
siRNA-MYH9-2693-as	UAGUUCUUCUUCCUUGGCCTT	
siRNA-negative control (S)	UUCUCCGAACGUGUCACGUTT	
siRNA-negative control (AS)	ACGUGACACGUUCGGAGAATT	

Note: the underline represents the introduced restriction endonuclease sites.

## Data Availability

The original contributions presented in this study are included in the article and Supplementary Data. Further inquiries can be directed to the corresponding author. The raw mass spectrometric data files that support the findings of this study are available at the iProX database (ID: IPX0004767000) (https://www.iprox.cn/page/subproject.html?id=IPX0004767001 (accessed on 21 July 2022)).

## Supplementary Materials

The following supporting information can be downloaded at https://www.mdpi.com/article/10.3390/ijms26189108/s1.

## Abbreviations

The following abbreviations are used in this manuscript:

DEV	duck enteritis virus
HRP	horseradish peroxidase
MOI	multiplicity of infection
STRING	Search Tool for the Retrieval of Interacting Genes
GO	Gene Ontology
KEGG	Kyoto Encyclopedia of Genes and Genomes
PPI	protein–protein interaction
Cyto D	cytochalasin D
Lat A	latrunculin A
BLEB	(-)-Blebbistatin
LMH	chicken liver hepatocellular carcinoma cell line
CEF	chicken embryo fibroblast
