# Supplementary material for: Proteomic Screening for Cellular Targets of the Duck Enteritis Virus Protein VP26 Reveals That the Host Actin–Myosin II Network Regulates the Proliferation of the Virus
Source: Int J Mol Sci. 2025 Sep 18;26(18):9108. doi: 10.3390/ijms26189108 (PMC12470233; doi:10.3390/ijms26189108)
Supplement: Supplementary file 1 [file ijms-26-09108-s001.zip › Supplement S1.pdf]

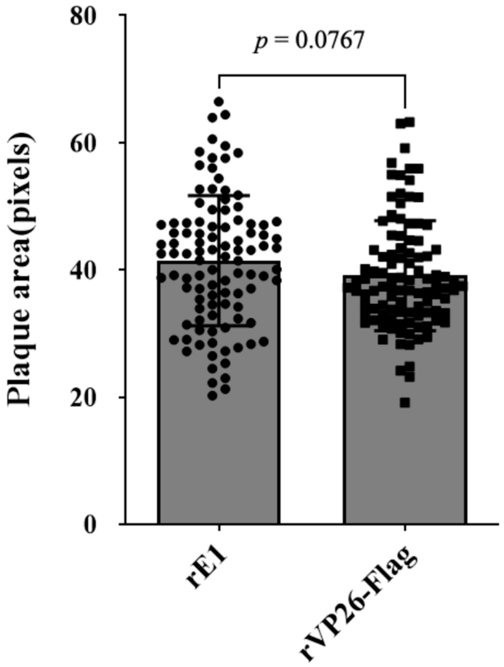

Sup Fig. S1 Plaque area measurement of rE1 and rVP26-Flag on CEF cells. The sizes of 103 plaques of each virus were measured with Image J software. Statistical significance was analyzed by two-tailed unpaired t test (*n*=103 per group).

A

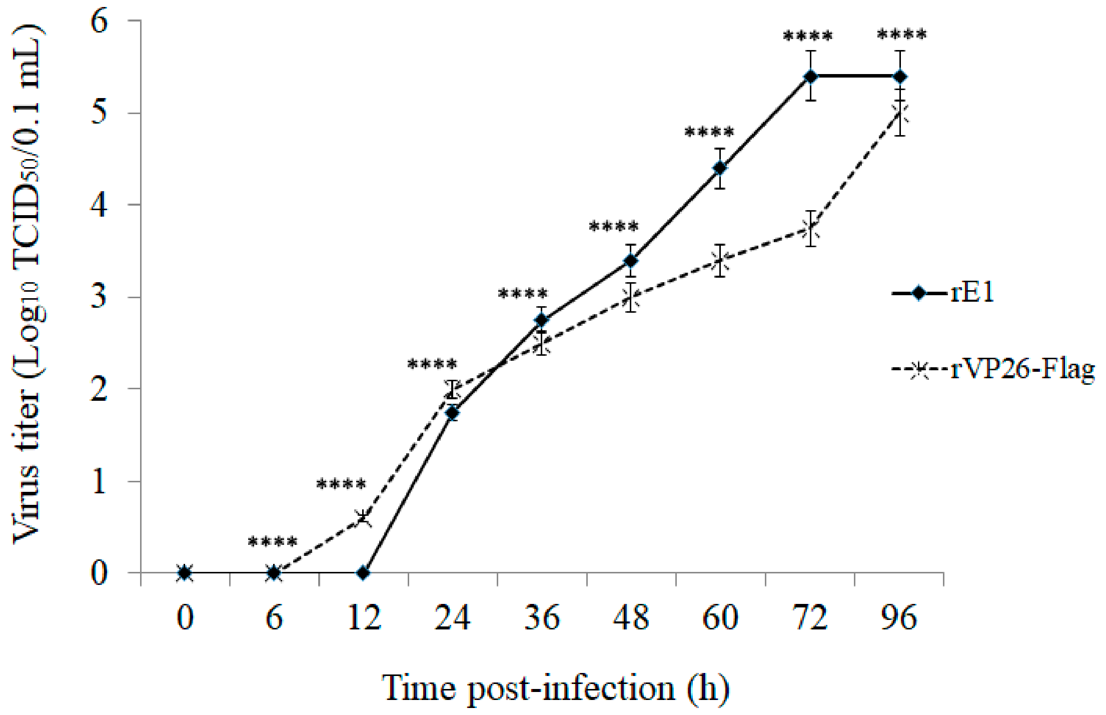

B

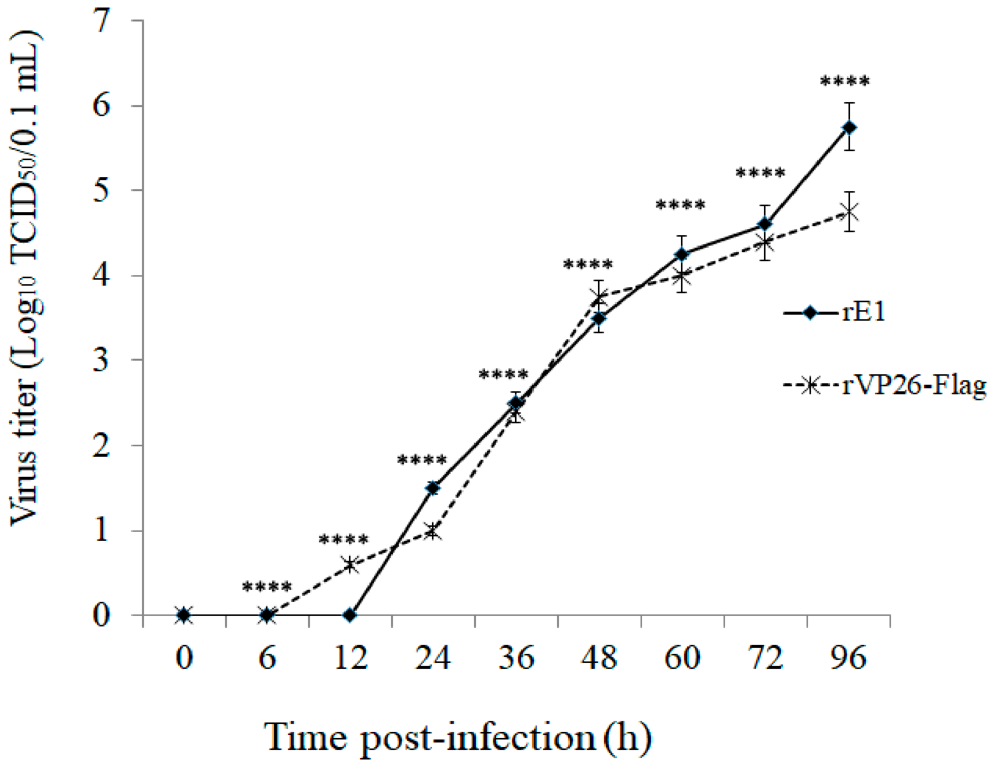

Sup Fig. S2 Multi-step growth curves of rE1 and rVP26-Flag. Comparison of the *in vitro* growth of viruses reconstructed with parental DEV. The virus titers of infected cells (A) and supernatants (B) were determined at different times (0, 6, 12, 24, 36, 48, 60, 72 and 96 h) after inoculation of approximately 0.02 MOI of cell-free viruses of

rE1, rVP26-Flag. The multi-step growth curves were computed from three independent experiments. Statistical significance was determined by two-way ANOVA followed by Tukey's multiple comparisons test. Asterisks indicates significant differences between rE1 and rVP26-Flag (****, $P < 0.0001$).
