# Supplementary material for: Proteomic Screening for Cellular Targets of the Duck Enteritis Virus Protein VP26 Reveals That the Host Actin–Myosin II Network Regulates the Proliferation of the Virus
Source: Int J Mol Sci. 2025 Sep 18;26(18):9108. doi: 10.3390/ijms26189108 (PMC12470233; doi:10.3390/ijms26189108)
Supplement: Supplementary file 1 [file ijms-26-09108-s001.zip › Supplement S2.pdf]

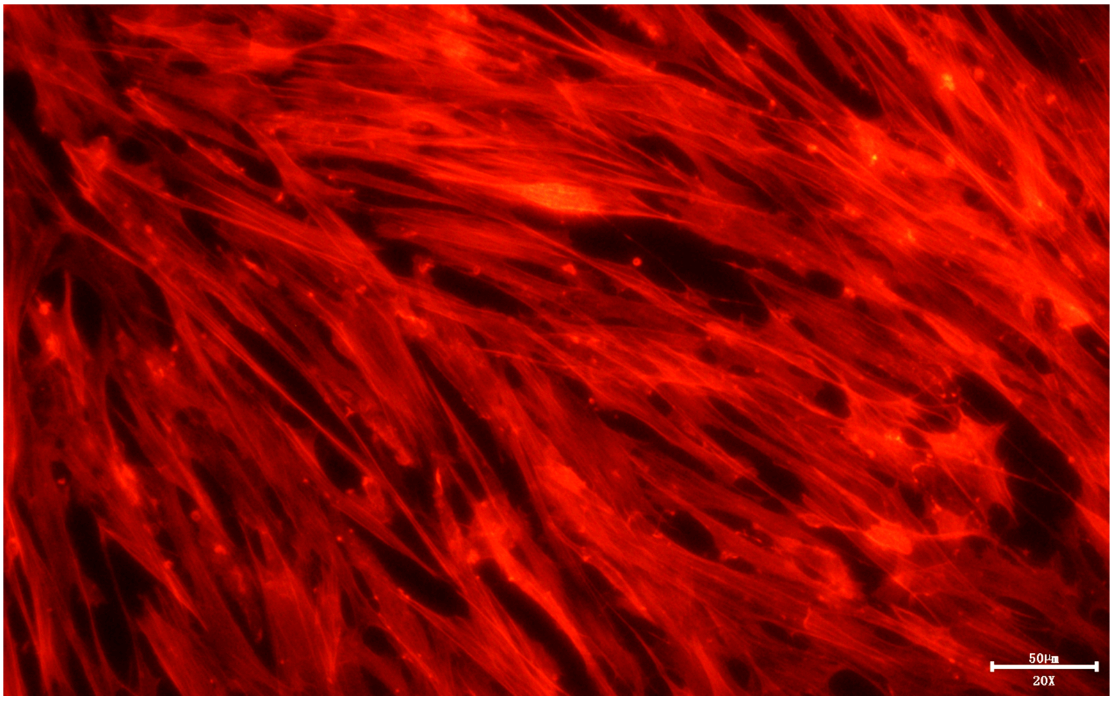

NC

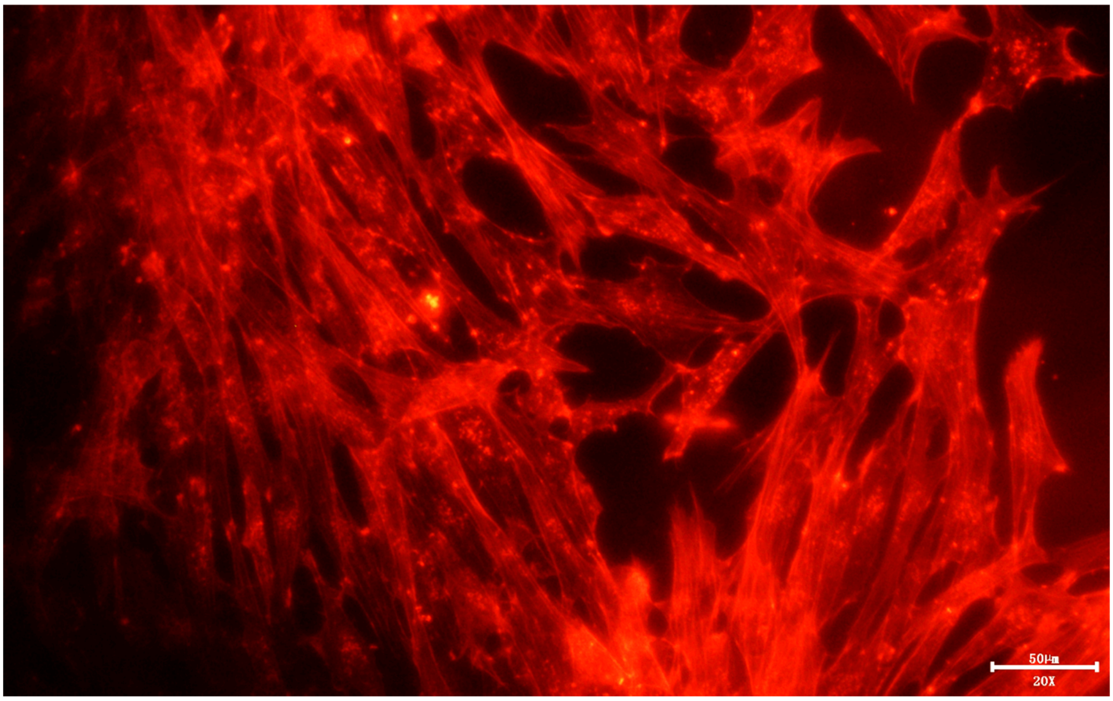

62.5 nM Cyto D

Compared with NC, more particles are formed, possibly produced by depolymerized myofilaments.

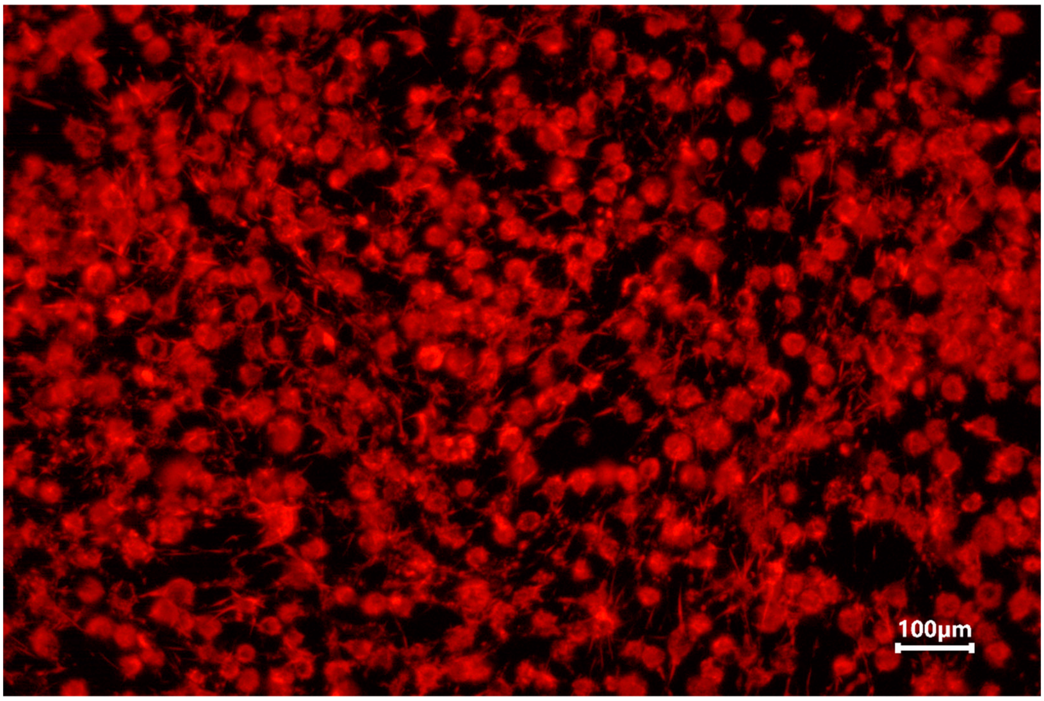

120 nM Cyto D

The morphology of CEFs treated with 120 nM Cyto D were completely disrupted.

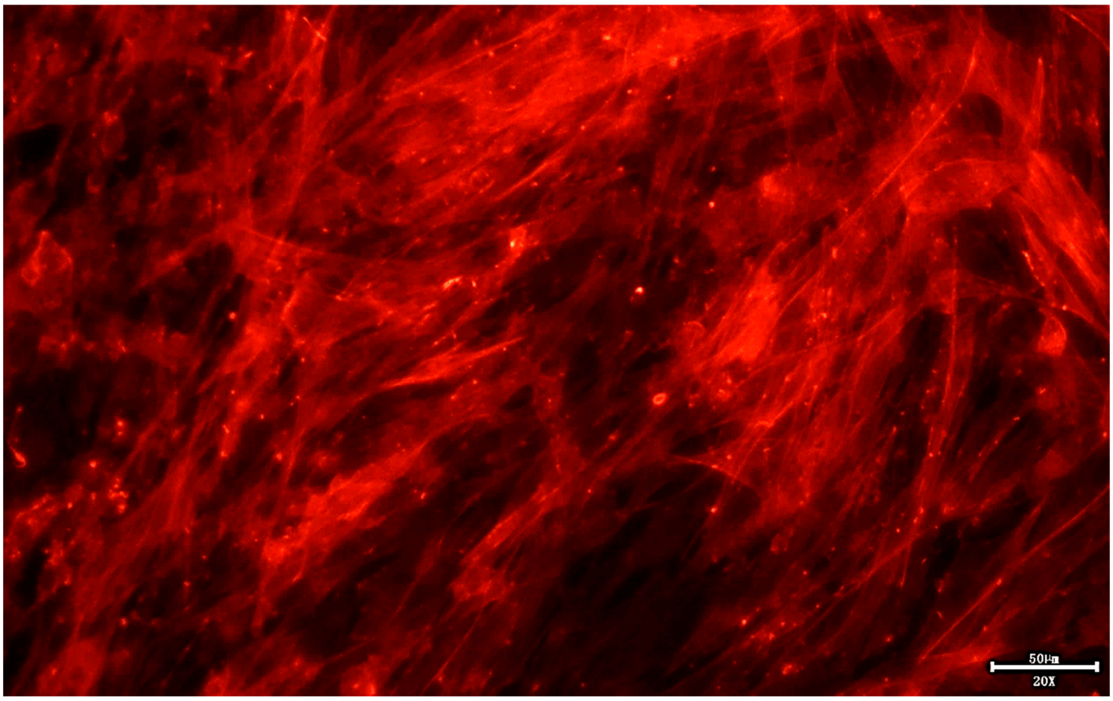

59.37 nM LatA

Compared with NC, more particles are formed, and the alignment of myofilaments becomes less orderly.

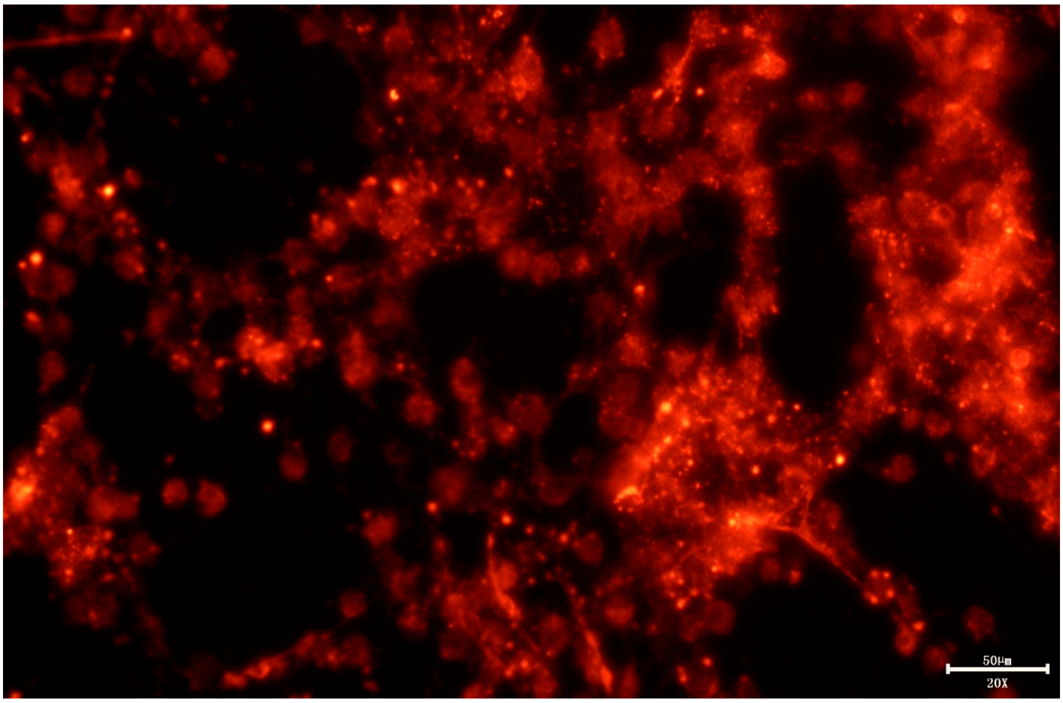

593.7 nM Lat A

The morphology of CEFs treated with 593.7 nM Lat A were completely disrupted.

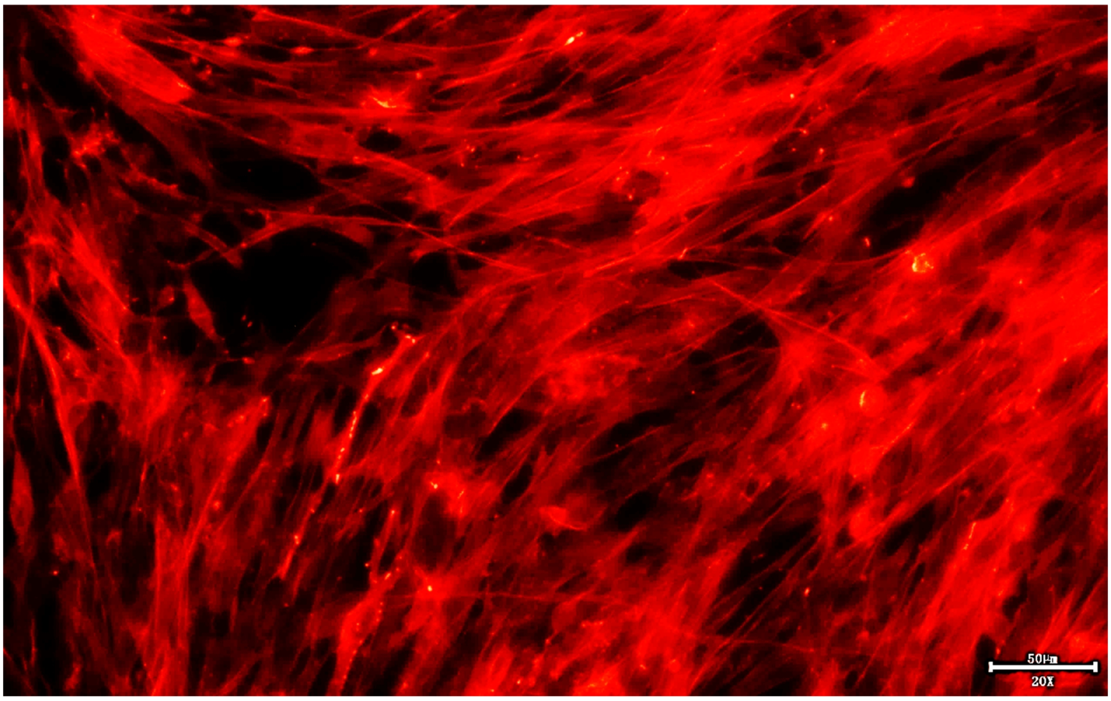

5 μM BLEB

Compared with NC, there was less effect on myofilament formation.

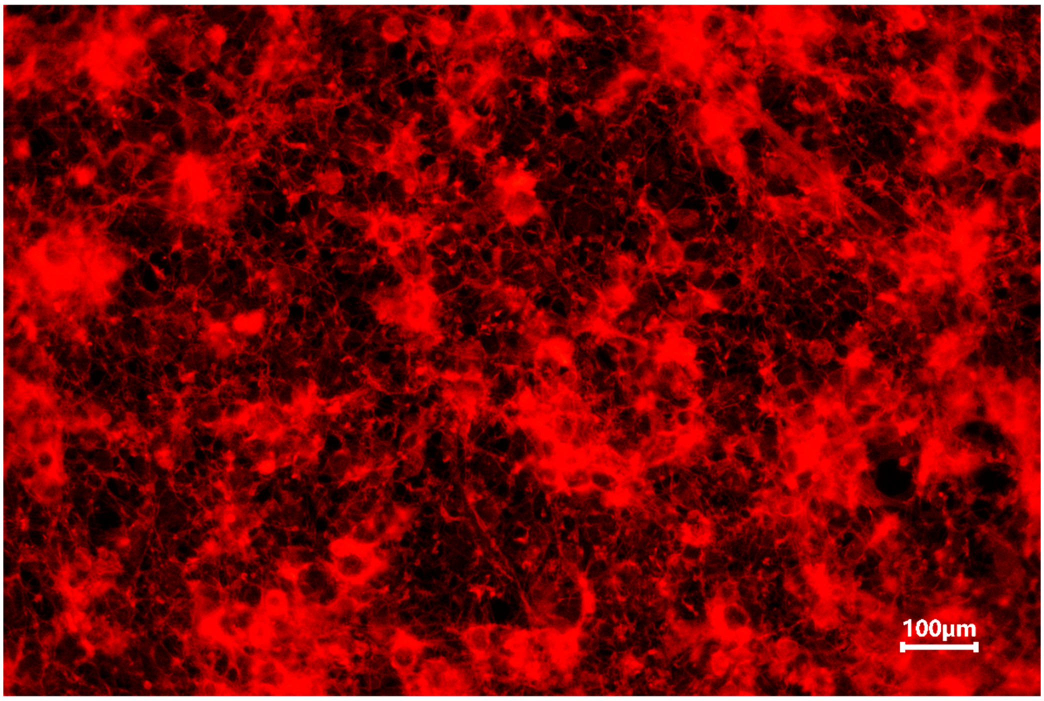

20 μM BLEB

The morphology of CEFs treated with 20 μM BLEB were completely disrupted.

Sup Fig. S3 Effect of drugs (Cyto D, LatA, BLEB) on microfilament cytoskeleton in CEFs.

CEF cells were pretreated with 125 nM or 62.5 nM Cyto D, 593.7 nM or 59.37 nM Lat A, 20 μM or 5 μM BLEB for 17 h, then fixed with 4% formaldehyde, and stained using Actin-Tracker Red-Rhodamine. And then the microfilament cytoskeleton in the treated cells were visualized using a fluorescence microscope to check the interference of drugs on the cytoskeleton of the cells.
