# Supplementary material for: Proteomic Screening for Cellular Targets of the Duck Enteritis Virus Protein VP26 Reveals That the Host Actin–Myosin II Network Regulates the Proliferation of the Virus
Source: Int J Mol Sci. 2025 Sep 18;26(18):9108. doi: 10.3390/ijms26189108 (PMC12470233; doi:10.3390/ijms26189108)
Supplement: Supplementary file 1 [file ijms-26-09108-s001.zip › Supplement S3-STRING analyses/STRING analysis.pdf]

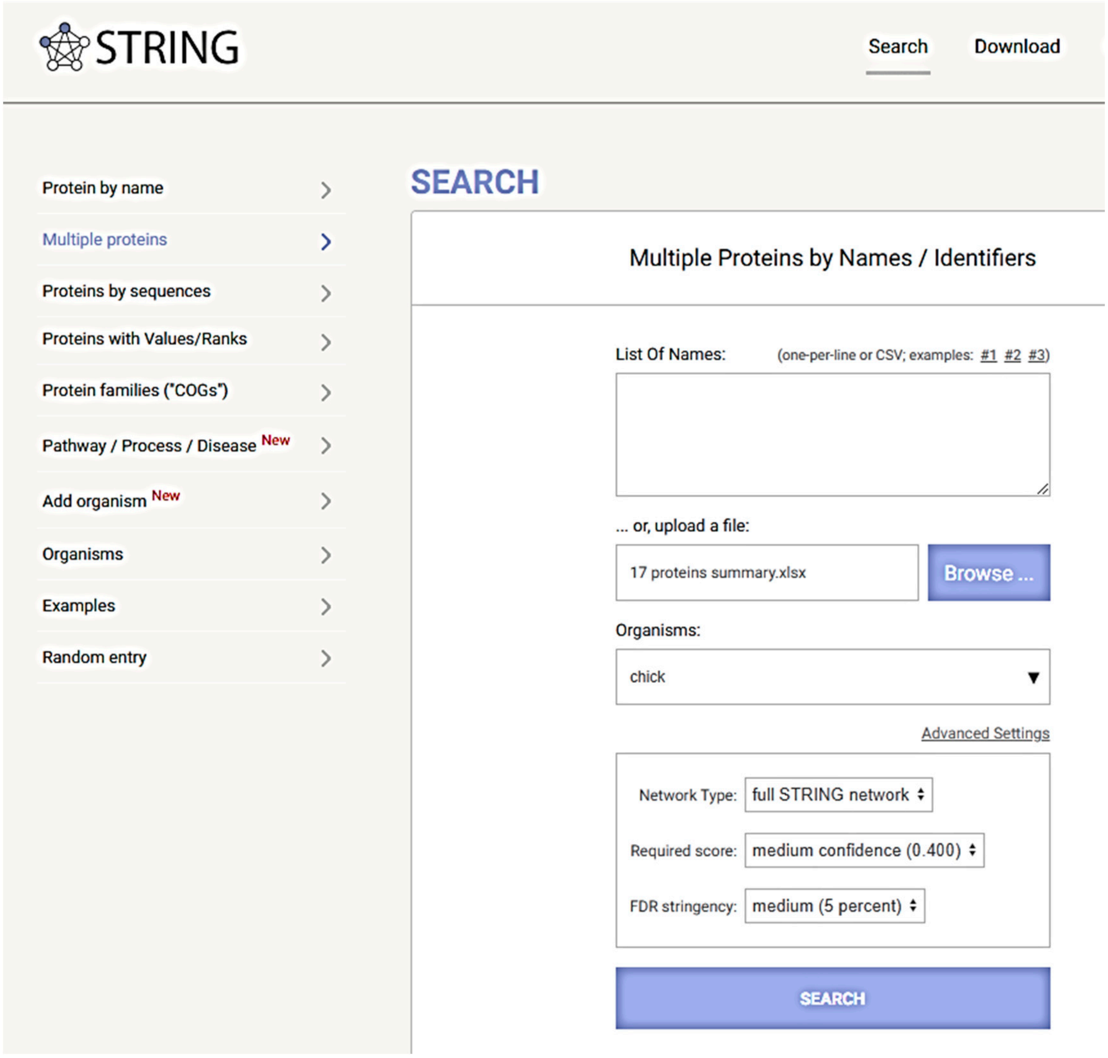
STRING
Search
Download
SEARCH
Protein by name
Multiple proteins
Proteins by sequences
Proteins with Values/Ranks
Protein families ("COGs")
Pathway / Process / Disease New
Add organism New
Organisms
Examples
Random entry
Multiple Proteins by Names / Identifiers
List Of Names:
(one-per-line or CSV; examples: #1 #2 #3)
... or, upload a file:
17 proteins summary.xlsx
Browse ...
Organisms:
chick
Advanced Settings
Network Type:
full STRING network
Required score:
medium confidence (0.400)
FDR stringency:
medium (5 percent)
SEARCH

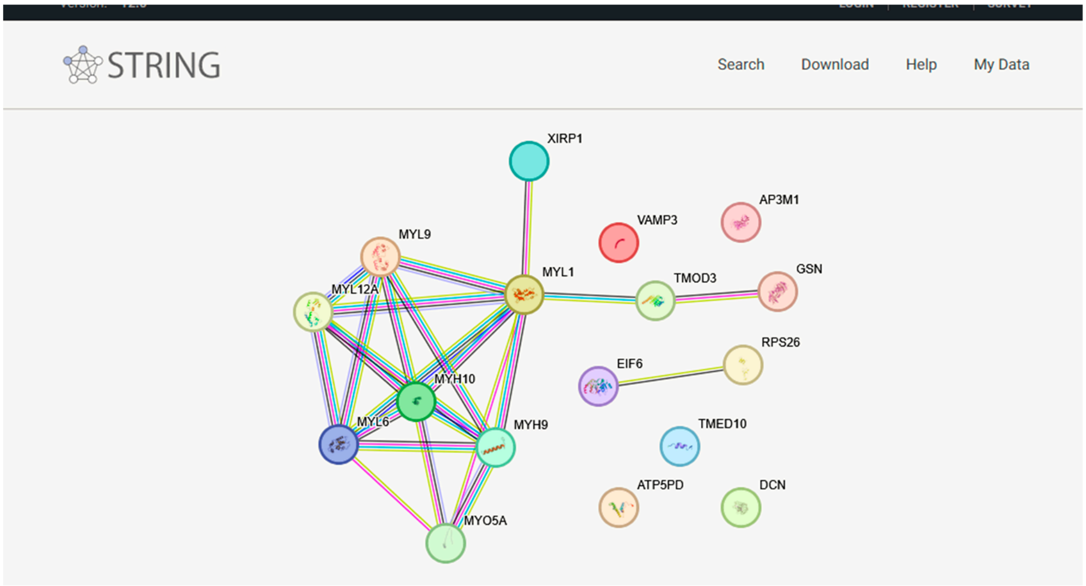
STRING
Search
Download
Help
My Data
XIRP1
AP3M1
VAMP3
MYL9
MYL1
TMOD3
GSN
MYL12A
RPS26
EIF6
MYH10
MYL6
MYH9
TMED10
ATP5PD
DCN
MYO5A

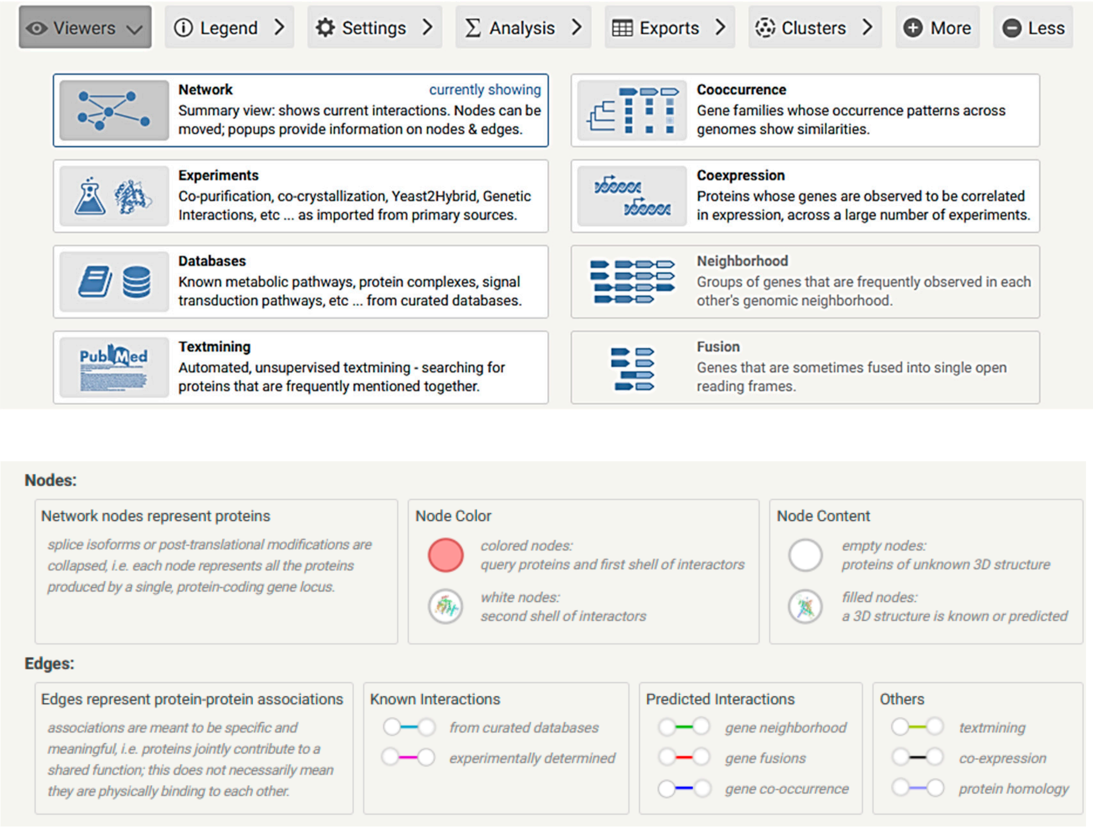

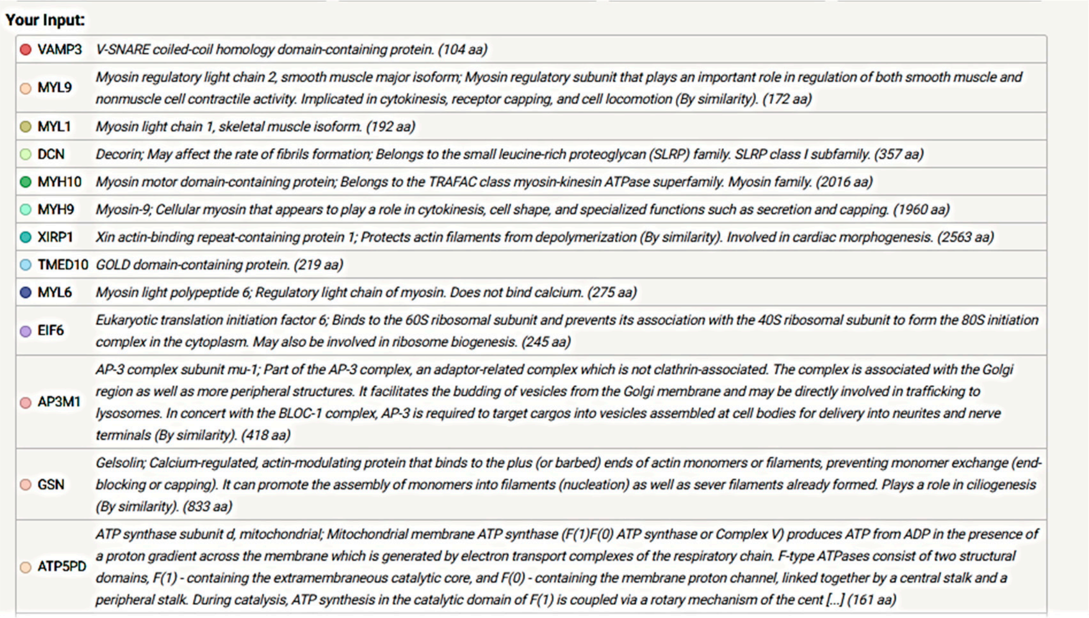

Your Input:

VAMP3	V-SNARE coiled-coil homology domain-containing protein. (104 aa)
MYL9	Myosin regulatory light chain 2, smooth muscle major isoform; Myosin regulatory subunit that plays an important role in regulation of both smooth muscle and nonmuscle cell contractile activity. Implicated in cytokinesis, receptor capping, and cell locomotion (By similarity). (172 aa)
MYL1	Myosin light chain 1, skeletal muscle isoform. (192 aa)
DCN	Decorin; May affect the rate of fibrils formation; Belongs to the small leucine-rich proteoglycan (SLRP) family. SLRP class I subfamily. (357 aa)
MYH10	Myosin motor domain-containing protein; Belongs to the TRAFAC class myosin-kinesin ATPase superfamily. Myosin family. (2016 aa)
MYH9	Myosin-9; Cellular myosin that appears to play a role in cytokinesis, cell shape, and specialized functions such as secretion and capping. (1960 aa)
XIRP1	Xin actin-binding repeat-containing protein 1; Protects actin filaments from depolymerization (By similarity). Involved in cardiac morphogenesis. (2563 aa)
TMED10	GOLD domain-containing protein. (219 aa)
MYL6	Myosin light polypeptide 6; Regulatory light chain of myosin. Does not bind calcium. (275 aa)
EIF6	Eukaryotic translation initiation factor 6; Binds to the 60S ribosomal subunit and prevents its association with the 40S ribosomal subunit to form the 80S initiation complex in the cytoplasm. May also be involved in ribosome biogenesis. (245 aa)
AP3M1	AP-3 complex subunit mu-1; Part of the AP-3 complex, an adaptor-related complex which is not clathrin-associated. The complex is associated with the Golgi region as well as more peripheral structures. It facilitates the budding of vesicles from the Golgi membrane and may be directly involved in trafficking to lysosomes. In concert with the BLOC-1 complex, AP-3 is required to target cargos into vesicles assembled at cell bodies for delivery into neurites and nerve terminals (By similarity). (418 aa)
GSN	Gelsolin; Calcium-regulated, actin-modulating protein that binds to the plus (or barbed) ends of actin monomers or filaments, preventing monomer exchange (end-blocking or capping). It can promote the assembly of monomers into filaments (nucleation) as well as sever filaments already formed. Plays a role in ciliogenesis (By similarity). (833 aa)
ATP5PD	ATP synthase subunit d, mitochondrial; Mitochondrial membrane ATP synthase (F(1)F(0) ATP synthase or Complex V) produces ATP from ADP in the presence of a proton gradient across the membrane which is generated by electron transport complexes of the respiratory chain. F-type ATPases consist of two structural domains, F(1) - containing the extramembraneous catalytic core, and F(0) - containing the membrane proton channel, linked together by a central stalk and a peripheral stalk. During catalysis, ATP synthesis in the catalytic domain of F(1) is coupled via a rotary mechanism of the cent [...] (161 aa)

DCN	*Decorin; May affect the rate of fibrils formation; Belongs to the small leucine-rich proteoglycan (SLRP) family. SLRP class I subfamily. (357 aa)*
MYH10	*Myosin motor domain-containing protein; Belongs to the TRAFAC class myosin-kinesin ATPase superfamily. Myosin family. (2016 aa)*
MYH9	*Myosin-9; Cellular myosin that appears to play a role in cytokinesis, cell shape, and specialized functions such as secretion and capping. (1960 aa)*
XIRP1	*Xin actin-binding repeat-containing protein 1; Protects actin filaments from depolymerization (By similarity). Involved in cardiac morphogenesis. (2563 aa)*
TMED10	*GOLD domain-containing protein. (219 aa)*
MYL6	*Myosin light polypeptide 6; Regulatory light chain of myosin. Does not bind calcium. (275 aa)*
EIF6	*Eukaryotic translation initiation factor 6; Binds to the 60S ribosomal subunit and prevents its association with the 40S ribosomal subunit to form the 80S initiation complex in the cytoplasm. May also be involved in ribosome biogenesis. (245 aa)*
AP3M1	*AP-3 complex subunit mu-1; Part of the AP-3 complex, an adaptor-related complex which is not clathrin-associated. The complex is associated with the Golgi region as well as more peripheral structures. It facilitates the budding of vesicles from the Golgi membrane and may be directly involved in trafficking to lysosomes. In concert with the BLOC-1 complex, AP-3 is required to target cargos into vesicles assembled at cell bodies for delivery into neurites and nerve terminals (By similarity). (418 aa)*
GSN	*Gelsolin; Calcium-regulated, actin-modulating protein that binds to the plus (or barbed) ends of actin monomers or filaments, preventing monomer exchange (end-blocking or capping). It can promote the assembly of monomers into filaments (nucleation) as well as sever filaments already formed. Plays a role in ciliogenesis (By similarity). (833 aa)*
ATP5PD	*ATP synthase subunit d, mitochondrial; Mitochondrial membrane ATP synthase (F(1)F(0) ATP synthase or Complex V) produces ATP from ADP in the presence of a proton gradient across the membrane which is generated by electron transport complexes of the respiratory chain. F-type ATPases consist of two structural domains, F(1) - containing the extramembraneous catalytic core, and F(0) - containing the membrane proton channel, linked together by a central stalk and a peripheral stalk. During catalysis, ATP synthesis in the catalytic domain of F(1) is coupled via a rotary mechanism of the cent [...] (161 aa)*
RPS26	*40S ribosomal protein S26; Belongs to the eukaryotic ribosomal protein eS26 family. (115 aa)*
MYL12A	*Myosin regulatory light chain 2, smooth muscle minor isoform; Myosin regulatory subunit that plays an important role in regulation of both smooth muscle and nonmuscle cell contractile activity. Implicated in cytokinesis, receptor capping, and cell locomotion (By similarity). (198 aa)*
TMOD3	*Uncharacterized protein. (352 aa)*
MYO5A	*Unconventional myosin-Va; Processive actin-based motor that can move in large steps approximating the 36-nm pseudo-repeat of the actin filament. Involved in melanosome transport. Also mediates the transport of vesicles to the plasma membrane. May also be required for some polarization process involved in dendrite formation (By similarity). (1830 aa)*

Your Current Organism:

Gallus gallus

NCBI taxonomy Id: 9031
Other names: G. gallus, Gallus domesticus, Gallus gallus domesticus, bantam, chicken, chickens, dwarf Leghorn chickens, red junglefowl

Basic Settings

Network type:

- [x] full STRING network (the edges indicate both functional and physical protein associations)
- [ ] physical subnetwork (the edges indicate that the proteins are part of a physical complex)

meaning of network edges:

- [x] evidence (line color indicates the type of interaction evidence)
- [ ] confidence (line thickness indicates the strength of data support)

active interaction sources:

- [x] Textmining
- [x] Experiments
- [x] Databases
- [x] Co-expression
- [x] Neighborhood
- [x] Gene Fusion
- [x] Co-occurrence

minimum required interaction score:

medium confidence (0.400)

max number of interactors to show:

1st shell: - none / query proteins only -

2nd shell: - none -

Advanced Settings

network display mode:

- [ ] static png (network is a simple bitmap image; not interactive)
- [x] interactive svg (network is a scalable vector graphic [SVG]; interactive)

network display options:

Enable node coloring mode

- [ ] enable 3D bubble design
- [ ] disable structure previews inside network bubbles
- [ ] center protein names on nodes
- [ ] show your query protein names
- [ ] hide disconnected nodes in the network
- [ ] hide protein names

12 protein name font size

Network Stats

number of nodes:	17	expected number of edges:	3
number of edges:	23	PPI enrichment p-value:	2.71e-14
average node degree:	2.71	*your network has significantly more interactions than expected (what does that mean?)*	
avg. local clustering coefficient:	0.592		

Functional enrichments in your network

explain columns

Biological Process (Gene Ontology)

GO-term	description	count in network	strength	signal	false discovery rate
GO:0031032	Actomyosin structure organization	6 of 80	1.87	2.47	7.08e-07
GO:0051146	Striated muscle cell differentiation	6 of 120	1.69	2.05	4.16e-06
GO:0030239	Myofibril assembly	4 of 39	2.01	1.75	0.00011
GO:0030036	Actin cytoskeleton organization	9 of 415	1.33	1.72	7.08e-07
GO:0097435	Supramolecular fiber organization	8 of 391	1.31	1.56	4.16e-06

(more ...)

Molecular Function (Gene Ontology)

GO-term	description	count in network	strength	signal	false discovery rate
GO:0032036	Myosin heavy chain binding	3 of 8	2.57	1.79	0.00017
GO:0000146	Microfilament motor activity	4 of 43	1.96	1.66	0.00017
GO:0051015	Actin filament binding	5 of 210	1.37	1.08	0.0012
GO:0008092	Cytoskeletal protein binding	9 of 825	1.03	1.01	7.98e-05
GO:0003779	Actin binding	6 of 405	1.16	0.94	0.0012

(more ...)

Cellular Component (Gene Ontology)

GO-term	description	count in network	strength	signal	false discovery rate
GO:0016460	Myosin II complex	6 of 26	2.36	4.23	2.37e-10
GO:0016459	Myosin complex	7 of 56	2.09	3.94	1.87e-10
GO:0015629	Actin cytoskeleton	9 of 379	1.37	2.05	1.57e-08
GO:0097513	Myosin II filament	2 of 2	2.99	1.48	0.00086
GO:0032982	Myosin filament	3 of 28	2.02	1.45	0.00063

(more ...)

Reference Publications (PubMed)

publication	(year) title	count in network	strength	signal	false discovery rate
PMID:37979277	(2023) A novel strategy for improving the stability of myofibrillar pr...	5 of 11	2.65	3.22	1.36e-07
PMID:36516724	(2023) Improvement of structural, physicochemical, and rheologica...	5 of 11	2.65	3.22	1.36e-07
PMID:22955375	(2013) Heavy and light roles: myosin in the morphogenesis of the h...	6 of 30	2.3	3.15	8.60e-08
PMID:24873380	(2014) Regulation of nonmuscle myosin II by tropomyosin.	5 of 16	2.49	3.0	3.17e-07
PMID:29395909	(2018) Localized Myosin II Activity Regulates Assembly and Plastic...	5 of 17	2.46	2.99	3.28e-07

(more ...)

Local Network Cluster (STRING)

cluster	description	count in network	strength	signal	false discovery rate
CL:21747	Myosin II complex, and Myosin light chain kinase activity	5 of 12	2.61	3.66	1.30e-08
CL:21691	Myosin II complex, and Troponin complex	6 of 43	2.14	3.35	1.30e-08
CL:21750	Mixed, incl. Muscle myosin complex, and Myosin II filament	4 of 7	2.75	3.28	1.12e-07
CL:21687	Mixed, incl. Contractile fiber, and Muscle protein	7 of 113	1.79	2.84	1.85e-08
CL:18046	Mixed, incl. Profilin, and Structural constituent of postsynaptic acti...	2 of 11	2.25	0.74	0.0284

KEGG Pathways

pathway	description	count in network	strength	signal	false discovery rate
gga04530	Tight junction	4 of 135	1.47	1.09	0.0016
gga04810	Regulation of actin cytoskeleton	4 of 171	1.36	1.01	0.0020
gga04270	Vascular smooth muscle contraction	3 of 102	1.46	0.85	0.0084

Subcellular Localization (COMPARTMENTS)

compartment	description	count in network	strength	signal	false discovery rate
GOCC:0016460	Myosin II complex	4 of 6	2.82	3.28	1.18e-07
GOCC:0015629	Actin cytoskeleton	8 of 184	1.63	2.65	8.83e-09
GOCC:0005859	Muscle myosin complex	3 of 5	2.77	2.37	1.08e-05
GOCC:0099512	Supramolecular fiber	8 of 333	1.37	1.86	2.89e-07
GOCC:0043292	Contractile fiber	5 of 134	1.57	1.65	3.61e-05

(more ...)

Annotated Keywords (UniProt)

keyword	description	count in network	strength	signal	false discovery rate
KW-0518	Myosin	7 of 46	2.18	4.43	1.66e-11
KW-0514	Muscle protein	4 of 30	2.12	2.35	4.62e-06
KW-0009	Actin-binding	5 of 112	1.64	1.9	9.24e-06
KW-0007	Acetylation	3 of 104	1.45	0.81	0.0113
KW-0112	Calmodulin-binding	2 of 27	1.86	0.75	0.0233

(more ...)

Protein Domains and Features (InterPro)

domain	description	count in network	strength	signal	false discovery rate
IPR002048	EF-hand domain	4 of 80	1.69	1.04	0.0039
IPR011992	EF-hand domain pair	4 of 92	1.63	1.03	0.0039
IPR004009	Myosin, N-terminal, SH3-like	2 of 7	2.45	0.7	0.0345
IPR001609	Myosin head, motor domain	2 of 9	2.34	0.67	0.0395

	Protein Domains (SMART)				
domain	description	count in network	strength	signal	false discovery rate
SM00242	Myosin. Large ATPases.	3 of 36	1.92	1.06	0.0045
SM00054	EF-hand, calcium binding motif	4 of 119	1.52	0.97	0.0045
SM00015	Short calmodulin-binding motif containing conserved Ile and Gln re...	3 of 75	1.6	0.79	0.0155

Functional enrichment visualization

Category: Biological Process (Gene Ontology)

Group terms by: similarity >= 0.8

Sort terms by: Signal

Nr. of terms shown: 10

Color Palette: mint - blue

Highlight selected terms:

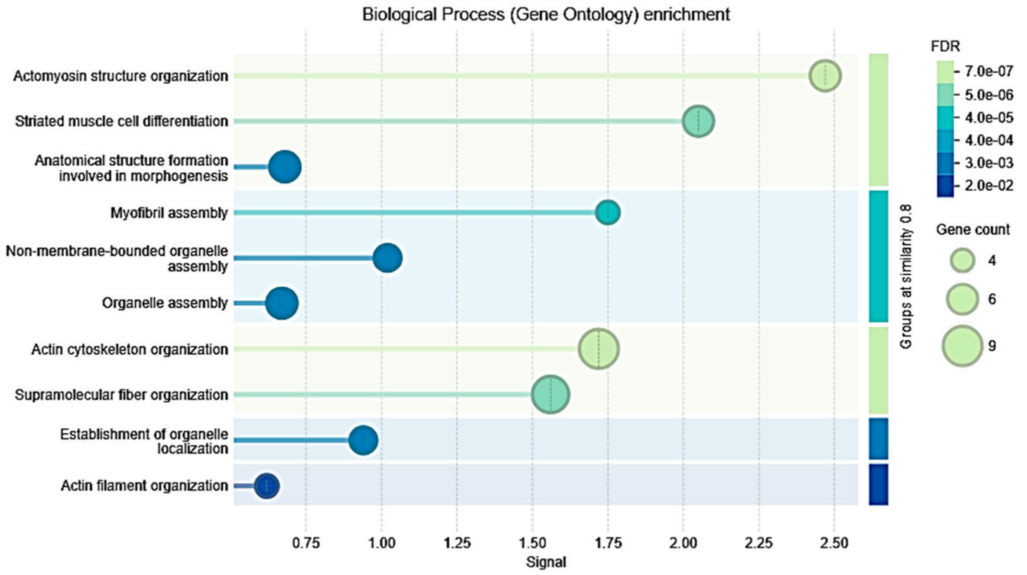

Download vector image (SVG) Download bitmap image (PNG)

Enrichment display settings

Merge rows by term similarity:	Don't merge	UPDATE
Maximum FDR shown:	FDR <= 0.05	
Minimum signal shown:	signal >= 0.01	
Minimum strength shown:	strength >= 0.01	
Minimum count in network:	2	
Row Visibility:	Display only selected rows	

Statistical background

For the above enrichment analysis, the following statistical background is assumed:	Whole Genome	ADD BACKGROUND	UPDATE

Save / Export

Biological Process (Gene Ontology)	download	*13 GO-terms significantly enriched; file-format: tab-delimited*
Molecular Function (Gene Ontology)	download	*9 GO-terms significantly enriched; file-format: tab-delimited*
Cellular Component (Gene Ontology)	download	*14 GO-terms significantly enriched; file-format: tab-delimited*
Reference Publications (PubMed)	download	*3359 publications significantly enriched; file-format: tab-delimited*
Local Network Cluster (STRING)	download	*5 clusters significantly enriched; file-format: tab-delimited*
KEGG Pathways	download	*3 pathways significantly enriched; file-format: tab-delimited*
Subcellular Localization (COMPARTMENTS)	download	*11 compartments significantly enriched; file-format: tab-delimited*
Annotated Keywords (UniProt)	download	*6 keywords significantly enriched; file-format: tab-delimited*
Protein Domains and Features (InterPro)	download	*4 domains significantly enriched; file-format: tab-delimited*
Protein Domains (SMART)	download	*3 domains significantly enriched; file-format: tab-delimited*
**All enriched terms** (without PubMed)	download	*68 enriched terms in 9 categories; file-format: tab-delimited*
Selected terms only	download	*no enriched terms selected (click on any term above to select)*

*There were **no** significant pathway enrichments observed in the following categories:*
Reactome Pathways, WikiPathways, Fission Yeast Phenotype Ontology (Monarch), Protein Domains (Pfam).
