# Supplementary material for: Proteomic Screening for Cellular Targets of the Duck Enteritis Virus Protein VP26 Reveals That the Host Actin–Myosin II Network Regulates the Proliferation of the Virus
Source: Int J Mol Sci. 2025 Sep 18;26(18):9108. doi: 10.3390/ijms26189108 (PMC12470233; doi:10.3390/ijms26189108)
Supplement: Supplementary file 1 [file ijms-26-09108-s001.zip › Supplement S4- Alignment of duck-original and chick-original protein sequences/AP3M1.pdf]

```
                      10         20         30         40         50         60
             ....|....| ....|....| ....|....| ....|....| ....|....| ....|....|
chick AP3M1  MIHSLFLINC SGDIFLEKHW KSVVSQSVCD YFFEAQEKAI DVENVPPVIS TPLHYLISIY
duck AP3M1   .......... .......... .......... .......... .......... ..H.......

                      70         80         90        100        110        120
             ....|....| ....|....| ....|....| ....|....| ....|....| ....|....|
chick AP3M1  RDKIFFVSVI QTEVPPLFVI EFLHRVADTF QDYFGECSET AIKDNVVIVY ELLEEMLDNG
duck AP3M1   .......... .......... .......... .......... .......... ..........

                     130        140        150        160        170        180
             ....|....| ....|....| ....|....| ....|....| ....|....| ....|....|
chick AP3M1  FPLATESNIL KELIKPPTIL RSVVNSITGS SNVGDTLPTG QLSNIPWRRA GVKYTNNEAY
duck AP3M1   .......... .......... .......... .......... .......... ..........

                     190        200        210        220        230        240
             ....|....| ....|....| ....|....| ....|....| ....|....| ....|....|
chick AP3M1  FDVIEEIDAI IDKSGSTVFA EIQGVIDSCI KLSGMPDLSL SFMNPRLLDD VSFHPCIRFK
duck AP3M1   ...V...... .......... .......... .......... .......... ..........

                     250        260        270        280        290        300
             ....|....| ....|....| ....|....| ....|....| ....|....| ....|....|
chick AP3M1  RWESERVLSF IPPDGNFRLI SYRVSSQNLV AIPVYVKHLI SFKENSSSGR FDVTIGPKQN
duck AP3M1   .......... .......... .......... ........M. .......... ..........

                     310        320        330        340        350        360
             ....|....| ....|....| ....|....| ....|....| ....|....| ....|....|
chick AP3M1  MGKTVEGVVM TVHMPKAVLN MNLTATQGSY TFDPVTKVLA WDVGKITPQK LPNLKGIVNL
duck AP3M1   .....S.... .......... .......... .........T .......... ..........

                     370        380        390        400        410
             ....|....| ....|....| ....|....| ....|....| ....|....| ....|...
chick AP3M1  QSGAPKPEEN PSLNIQFKIQ QLAISGLKVN RLDMYGEKYK PFKGVKYITK AGKFQVRT
duck AP3M1   .......... .......... .......... .......... .......... ........
```
