# Supplementary material for: Proteomic Screening for Cellular Targets of the Duck Enteritis Virus Protein VP26 Reveals That the Host Actin–Myosin II Network Regulates the Proliferation of the Virus
Source: Int J Mol Sci. 2025 Sep 18;26(18):9108. doi: 10.3390/ijms26189108 (PMC12470233; doi:10.3390/ijms26189108)
Supplement: Supplementary file 1 [file ijms-26-09108-s001.zip › Supplement S4- Alignment of duck-original and chick-original protein sequences/ATP5H.pdf]

```
                     10         20         30         40         50         60
             ....|....| ....|....| ....|....| ....|....| ....|....| ....|....|
chick ATP5H  ---------- ---------- ---------- ---------- ---------- --MAARRTAV
duck ATP5PD  MPGPAQRRPA AITARRFRFP RAEPEAEPEG GEGAGLPARC SAGDGGRGSR RS..G..A.L

                     70         80         90        100        110        120
             ....|....| ....|....| ....|....| ....|....| ....|....| ....|....|
chick ATP5H  KAIDWAAFAE RVPANQRAMF NALKTRSDAL SARLAALPEK PPAIDWTYYK TAVAKAGMVD
duck ATP5PD  .......... .......... .......... .....S.... ..T...AH.. A.I.......

                    130        140        150        160        170        180
             ....|....| ....|....| ....|....| ....|....| ....|....| ....|....|
chick ATP5H  EFQKKFSALK VPEPVDTQTA KIDAQEQEAA KGIVEYVKAS KARIAEYEQQ LQKLRSMIPF
duck ATP5PD  .......... .......... .......... .NTA...Q.. .....Q...H ....K.....

                    190        200        210
             ....|....| ....|....| ....|....| ...
chick ATP5H  EQMTFEDLHE AFPETRLDRE KYPFWPHKPI ADL
duck ATP5PD  ....N..MA. .....K..M. .N.....R.. ...
```
