# Supplementary material for: Proteomic Screening for Cellular Targets of the Duck Enteritis Virus Protein VP26 Reveals That the Host Actin–Myosin II Network Regulates the Proliferation of the Virus
Source: Int J Mol Sci. 2025 Sep 18;26(18):9108. doi: 10.3390/ijms26189108 (PMC12470233; doi:10.3390/ijms26189108)
Supplement: Supplementary file 1 [file ijms-26-09108-s001.zip › Supplement S4- Alignment of duck-original and chick-original protein sequences/DCN.pdf]

```
                   10         20         30         40         50         60
           ....|....| ....|....| ....|....| ....|....| ....|....| ....|....|
chick DCN  MRLVLLFVLL LPVCLATRFH QKGLFDFMIE DEGSADMAPT DDPVISGFGP VCPFRCQCHL
duck DCN   ...A...I.. ......KP.. .M.....L.. .......... .......... ..........

                   70         80         90        100        110        120
           ....|....| ....|....| ....|....| ....|....| ....|....| ....|....|
chick DCN  RVVQCSDLGL ERVPKDLPPD TTLLDLQNNK ITEIKEGDFK NLKNLHALIL VNNKISKISP
duck DCN   .......... .......... .......... .......... .......... ..........

                  130        140        150        160        170        180
           ....|....| ....|....| ....|....| ....|....| ....|....| ....|....|
chick DCN  AAFAPLKKLE RLYLSKNNLK ELPENMPKSL QEIRAHENEI SKLRKAVFNG LNQVIVLELG
duck DCN   .......... .......... .......... .......... .......... ..........

                  190        200        210        220        230        240
           ....|....| ....|....| ....|....| ....|....| ....|....| ....|....|
chick DCN  TNPLKSSGIE NGAFQGMKRL SYIRIADTNI TSIPKGLPPS LTELHLDGNK ISKIDAEGLS
duck DCN   .......... .......... .......... .......... .......... ..........

                  250        260        270        280        290        300
           ....|....| ....|....| ....|....| ....|....| ....|....| ....|....|
chick DCN  GLTNLAKLGL SFNSISSVEN GSLNNVPHLR ELHLNNNELV RVPSGLGEHK YIQVVYLHNN
duck DCN   .......... .......... .......... .......... .......... ..........

                  310        320        330        340        350
           ....|....| ....|....| ....|....| ....|....| ....|....| ....|..
chick DCN  KIASIGINDF CPLGYNTKKA TYSGVSLFSN PVQYWEIQPS AFRCIHERSA VQIGNYK
duck DCN   ....V..... .......... .......... .......... .......... I......
```
