# Supplementary material for: Proteomic Screening for Cellular Targets of the Duck Enteritis Virus Protein VP26 Reveals That the Host Actin–Myosin II Network Regulates the Proliferation of the Virus
Source: Int J Mol Sci. 2025 Sep 18;26(18):9108. doi: 10.3390/ijms26189108 (PMC12470233; doi:10.3390/ijms26189108)
Supplement: Supplementary file 1 [file ijms-26-09108-s001.zip › Supplement S4- Alignment of duck-original and chick-original protein sequences/EIF6.pdf]

```
                    10         20         30         40         50         60
            ....|....| ....|....| ....|....| ....|....| ....|....| ....|....|
chick EIF6  MAVRASFENN NELGCFAKLT NAYCLVAIGG SENFYSVFEG ELFGTIPVVH ASIAGCRIIG
duck EIF6   .......... .......... .......... .......... .......... ..........

                    70         80         90        100        110        120
            ....|....| ....|....| ....|....| ....|....| ....|....| ....|....|
chick EIF6  RMCVGNRHGL LVPSSTTDQE LQHIRNSLPD SVRIQRVEER LSALGNVTTC NDYVALVHPD
duck EIF6   .......... .......... .......... .......... .......... ..........

                   130        140        150        160        170        180
            ....|....| ....|....| ....|....| ....|....| ....|....| ....|....|
chick EIF6  LDRETEEILA DVLKVEVFRQ TVADQVLVGS YCVFSNQGGI VHPKTSIEDQ DELSSLLQVP
duck EIF6   .......... .......... .......... .......... .......D.. ..........

                   190        200        210        220        230        240
            ....|....| ....|....| ....|....| ....|....| ....|....| ....|....|
chick EIF6  LVAGTVNRGS EVIGAGMVVN DWCAFCGLDT TSTELSVIES IFKLNEAQPS TIATNMRDSL
duck EIF6   .......... ...A...... .......... .......... ..R....... ..........

            ....|
chick EIF6  IDSLA
duck EIF6   ....T
```
