# Supplementary material for: Proteomic Screening for Cellular Targets of the Duck Enteritis Virus Protein VP26 Reveals That the Host Actin–Myosin II Network Regulates the Proliferation of the Virus
Source: Int J Mol Sci. 2025 Sep 18;26(18):9108. doi: 10.3390/ijms26189108 (PMC12470233; doi:10.3390/ijms26189108)
Supplement: Supplementary file 1 [file ijms-26-09108-s001.zip › Supplement S4- Alignment of duck-original and chick-original protein sequences/GSN.file.pdf]

https://www.uniprot.org/uniprotkb/O93510/entry

> chick GSN

MGKQGFGYIFLTIFCTMALKLNCVSSVSVAGLGYVVTAAVVLSA

VPVSMVEHAEFSKAGKEPGLQIWRIEKFDLVPVPKNLYGDFFTGDSYLVLNTIRQRSG

NLQYDLHFWLGDESSQDERGAAAIFTVQMDDYLQGKAVQHREVQGHESSTFLGYFKSG

IKYKAGGVASGFRHVVPNEVTVQRLLQVKGRRTVRATEVPVSWESFNTGDCFILDLGS

NIYQWCGSNSNRQERLKATVLAKGIRDNERNGRAKVFVSEEGAEREEMLQVLGPKPSL

PQGASDDTKTDTANRKLAKLYKVSNGAGNMAVSLVADENPFSQAALNTEDCFILDHGT

DGKIFVWKGRSANSDERKAALKTATDFIEKMGYPKHTQVQVLPESGETPLFKQFFKNW

RDKDQTEGLGEAYISGHVAKIEKVPFDAATLHTSRAMAAQHGMEDDGSGKKQIWRIEG

SEKVPVDPATYGQFYGGDSYIILYDYRHAGKQGQIIYTWQGAHSTQDEIATSAFLTVQ

LDEELGGSPVQKRVVQGKEPPHLMSMFGGKPLIVYKGGTSREGGQTTPAQTRLFQVRS

STSGATRAVELDPAASQLNSNDAFVLKTPSAAYLWVGRGSNSAELSGAQELLKVLGAR

PVQVSEGREPDNFWVALGGKAPYRTSPRLKDKKMDAHPPRLFACSNKSGRFTIEEVPG

DLTQDDLATDDVMILDTWDQVFVWIGKDAQEEEKTEALKSAKRYIETDPASRDKRTPV
TLVKQGLEPPTFSGWFLGWDDDYWSVDPLQRAMADVDV

PREDICTED: Anas platyrhynchos gelsolin (GSN), transcript variant X1, mRNA

NCBI Reference Sequence: XM_005030036.6

>Duck GSN

MGRQDFGYVFLTVFCTMALKLNCVSSMSVAGLGYVVTAAVVLSA

VPVSMVEHAEFSKAGKEPGLQIWRIEKFDLVPVPKNLYGDFFTGDSYLVLNTIKQRNG

NLQYDLHFWLGDESSQDERGAAAIFTVQMDDYLQGKAVQHREVQGHESATFLGYFKSG

IKYKAGGVASGFRHVVPNEVTVQRLLQVKGRRAVRATEVPVTWESFNTGDCFILDLGS

NIYQWCGSNSNRQERLKATVLAKGIRDNERNGRAKVFVSDEGSEREEMLQVLGPKPTL

PVGTPDDTKTDTANRKLAKLYKVSNGAGNMAVSLVADENPFSQAALNTDDCFILDHGT

DGKIFVWKGRSANSEERKAALKTASEFIDKMSYPKHTQIQVLPESGETPLFKQFFKNW

RDKDQTEGLGQAYISGHVAKIEKVPFDAATLHTSKAMAAQHGMEDDGSGRKQIWRIEG

SEKVPVDPSTYGQFYGGDSYIILYNYQHAGKQGQIIYTWQGAHSTQDEIATSAFLTVQ

LDEELGGSPVQKRVVQGKEPPHLMSMFGGKPLIVYKGGTSRGGGQTTPAETRLFQVRS

STSGATRAVELDPAASQLNSNDAFVLKTPSAAYLWVGQGASNAEKSGAQELLNVLGAR

SVQVSEGREPENFWAVLGGKAPYRTSPRLKDKKMDAHPPRLFACSNKSGRFTIEEVPG

DLTQDDLATDDVMILDTWDQVFVWIGKDAQEEEKTEALKSAKRYIETDPASRDKRTPV
TLVKQGFEPPTFSGWFLGWDDDYWSVDPLQRAMADVDV
