# Supplementary material for: Proteomic Screening for Cellular Targets of the Duck Enteritis Virus Protein VP26 Reveals That the Host Actin–Myosin II Network Regulates the Proliferation of the Virus
Source: Int J Mol Sci. 2025 Sep 18;26(18):9108. doi: 10.3390/ijms26189108 (PMC12470233; doi:10.3390/ijms26189108)
Supplement: Supplementary file 1 [file ijms-26-09108-s001.zip › Supplement S4- Alignment of duck-original and chick-original protein sequences/GSN.pdf]

```
                    10         20         30         40         50         60
           ....|....| ....|....| ....|....| ....|....| ....|....| ....|....|
>chick GSN MGKQGFGYIF LTIFCTMALK LNCVSSVSVA GLGYVVTAAV VLSAVPVSMV EHAEFSKAGK
>duck GSN  ..R.D...V. ..V....... ......M... .......... .......... ..........

                    70         80         90        100        110        120
           ....|....| ....|....| ....|....| ....|....| ....|....| ....|....|
>chick GSN EPGLQIWRIE KFDLVPVPKN LYGDFFTGDS YLVLNTIRQR SGNLQYDLHF WLGDESSQDE
>duck GSN  .......... .......... .......... .......K.. N......... ..........

                   130        140        150        160        170        180
           ....|....| ....|....| ....|....| ....|....| ....|....| ....|....|
>chick GSN RGAAAIFTVQ MDDYLQGKAV QHREVQGHES STFLGYFKSG IKYKAGGVAS GFRHVVPNEV
>duck GSN  .......... .......... .......... A......... .......... ..........

                   190        200        210        220        230        240
           ....|....| ....|....| ....|....| ....|....| ....|....| ....|....|
>chick GSN TVQRLLQVKG RRTVRATEVP VSWESFNTGD CFILDLGSNI YQWCGSNSNR QERLKATVLA
>duck GSN  .......... ..A....... .T........ .......... .......... ..........

                   250        260        270        280        290        300
           ....|....| ....|....| ....|....| ....|....| ....|....| ....|....|
>chick GSN KGIRDNERNG RAKVFVSEEG AEREEMLQVL GPKPSLPQGA SDDTKTDTAN RKLAKLYKVS
>duck GSN  .......... .......D.. S......... ....T..V.T P......... ..........

                   310        320        330        340        350        360
           ....|....| ....|....| ....|....| ....|....| ....|....| ....|....|
>chick GSN NGAGNMAVSL VADENPFSQA ALNTEDCFIL DHGTDGKIFV WKGRSANSDE RKAALKTATD
>duck GSN  .......... .......... ....D..... .......... ........E. ........SE

                   370        380        390        400        410        420
           ....|....| ....|....| ....|....| ....|....| ....|....| ....|....|
>chick GSN FIEKMGYPKH TQVQVLPESG ETPLFKQFFK NWRDKDQTEG LGEAYISGHV AKIEKVPFDA
>duck GSN  ..D..S.... ..I....... .......... .......... ..Q....... ..........

                   430        440        450        460        470        480
           ....|....| ....|....| ....|....| ....|....| ....|....| ....|....|
>chick GSN ATLHTSRAMA AQHGMEDDGS GKKQIWRIEG SEKVPVDPAT YGQFYGGDSY IILYDYRHAG
>duck GSN  ......K... .......... .R........ ........S. .......... ....N.Q...

                   490        500        510        520        530        540
           ....|....| ....|....| ....|....| ....|....| ....|....| ....|....|
>chick GSN KQGQIIYTWQ GAHSTQDEIA TSAFLTVQLD EELGGSPVQK RVVQGKEPPH LMSMFGGKPL
>duck GSN  .......... .......... .......... .......... .......... ..........

                   550        560        570        580        590        600
           ....|....| ....|....| ....|....| ....|....| ....|....| ....|....|
>chick GSN IVYKGGTSRE GGQTTPAQTR LFQVRSSTSG ATRAVELDPA ASQLNSNDAF VLKTPSAAYL
>duck GSN  .........G .......E.. .......... .......... .......... ..........

                   610        620        630        640        650        660
           ....|....| ....|....| ....|....| ....|....| ....|....| ....|....|
>chick GSN WVGRGSNSAE LSGAQELLKV LGARPVQVSE GREPDNFWVA LGGKAPYRTS PRLKDKKMDA
>duck GSN  ...Q.ASN.. K.......N. ....S..... ....E...AV .......... ..........

                   670        680        690        700        710        720
           ....|....| ....|....| ....|....| ....|....| ....|....| ....|....|
>chick GSN HPPRLFACSN KSGRFTIEEV PGDLTQDDLA TDDVMILDTW DQVFVWIGKD AQEEEKTEAL
>duck GSN  .......... .......... .......... .......... .......... ..........

                   730        740        750        760        770
           ....|....| ....|....| ....|....| ....|....| ....|....| ....|...
>chick GSN KSAKRYIETD PASRDKRTPV TLVKQGLEPP TFSGWFLGWD DDYWSVDPLQ RAMADVDV
>duck GSN  .......... .......... ......F... .......... .......... ........
```
