# Supplementary material for: Proteomic Screening for Cellular Targets of the Duck Enteritis Virus Protein VP26 Reveals That the Host Actin–Myosin II Network Regulates the Proliferation of the Virus
Source: Int J Mol Sci. 2025 Sep 18;26(18):9108. doi: 10.3390/ijms26189108 (PMC12470233; doi:10.3390/ijms26189108)
Supplement: Supplementary file 1 [file ijms-26-09108-s001.zip › Supplement S4- Alignment of duck-original and chick-original protein sequences/MYH10.file.pdf]

https://www.uniprot.org/uniprotkb/Q789A6/entry

>chick MYH10

MAQRSGQEDPERYLFVDRAVIYNPATQADWTAKKLVWIPSERHGFEAASIKEERGDEVLVELAENGKKALV
NKDDIQKMNPPKFSKVEDMAELTCLNEASVLHNLKDRYYSGLIYTYSGLFCVVINPYKNLPIYSENIIEMY
RGKKRHEMPPHIYAISESAYRCMLQDREDQSILCTGESGAGKTENTKKVIQYLAHVASSHKGRKDHNIPGE
LERQLLQANPILESFGNAKTVKNDNSSRFGKFIRINFDVTGYIVGANIETYLLEKSRAVRQAKDERTFHIF
YQLLAGAGEHLKSDLLLEGFNNYRFLSNGYIPIPGQQDKDNFQETMEAMHIMGFSHDEILSMLKVVSSVLQ
FGNISFKKERNTDQASMPENTVAQKLCHLLGMNVMEFTRAILTPRIKVGRDYVQKAQTKEQADFAVEALAK
ATYERLFRWLVHRINKALDRTKRQGASFIGILDIAGFEIFELNSFEQLCINYTNEKLQQLFNHTMFILEQE
EYQREGIEWNFIDFGLDLQPCIDLIERPANPPGVLALLDEECWFPKATDKTFVEKLVQEQGTHSKFQKPRQ
LKDKADFCIIHYAGKVDYKADEWLMKNMDPLNDNVATLLHQSSDKFVAELWKDVDRIVGLDQVTGITETAF
GSAYKTKKGMFRTVGQLYKESLTKLMATLRNTNPNFVRCIIPNHEKRAGKLDPHLVLDQLRCNGVLEGIRI
CRQGFPNRIVFQEFRQRYEILTPNAIPKGFMDGKQACERMIRALELDPNLYRIGQSKIFFRAGVLAHLEEE
RDLKITDIIIFFQAVCRGYLARKAFAKKQQQLSALKILQRNCAAYLKLRHWQWWRVFTKVKPLLQVTRQEE
ELQAKDEELMKVKEKQTKVEAELEEMERKHQQLLEEKNILAEQLQAETELFAEAEEMRARLAAKKQELEEI
LHDLESRVEEEEEERNQILQNEKKKMQGHIQDLEEQLDEEEGARQKLQLEKVTAEAKIKKMEEEILLLEDQN
SKFLKEKKLMEDRIAECTSQLAEEEEKAKNLAKLKNKQEMMITDLEERLKKEEKTRQELEKAKRKLDGETT
DLQDQIAELQAQIEELKIQLAKKEEELQAALARGDEEAVQKNNALKVIRELQAQIAELQEDLESEKASRNK
AEKQKRDLSEELEALKTELEDTLDTTAAQQELRTKREQEVAELKKAIEEETKNHEAQIQEIRQRHATALEE
LSEQLEQAKRFKANLEKNKQGLESDNKELACEVKVLQQVKAESEHKRKKLDAQVQELTAKVTEGERLRVEL
AEKANKLQNELDNVSSLLEEAEKKGIKFAKDAASLESQLQDTQELLQEETRQKLNLSSRIRQLEEEKNNLQ
EQQEEEEEARKNLEKQMLALQAQLAEAKKKVDDDLGTIEGLEENKKKLLKDMESLSQRLEEKAMAYDKLEK
TKNRLQQELDDLMVDLDHQRQIVSNLEKKQKKFDQMLAEEKNISARYAEERDRAEAEAREKETKALSLARA
LEEALEAKEEFERQNKQLRADMEDLMSSKDDVGKNVHELEKSKRTLEQQVEEMRTQLEELEDELQATEDAK
LRLEVNMQAMKAQFERDLQARDEQNEEKKRMLVKQVRELEAELEDERKQRALAVAAKKKMEMDLKDLEGQI
EAANKARDEAIKQLRKLQAQMKDYQRELEEARASRDEIFAQSKESEKKLKGLEAEILQLQEEFAASERARR
HAEQERDELADEIANSASGKSALLDEKRRLEARIAQLEEELEEEQSNMELLNERFRKTTLQVDTLNSELAG
ERSAAQKSENARQQLERQNKELKAKLQELEGSVKSKFKATISTLEAKIAQLEEQLEQEAKERAAANKLVRR
TEKKLKEVFMQVEDERRHADQYKEQMEKANARMKQLKRQLEEAEEEATRANASRRKLQRELDDATEANEGL
SREVSTLKNRLRRGGPITFSSSRSGRRQLHIEGASLELSDDDAESKGSDVNEAQPTPAE

https://www.ncbi.nlm.nih.gov/gene/101789798

>duck MYH10

MAQRSGQEDPERYLFVDRAVIYNPATQADWTAKKLVWIPSERHG

FEAASIKEEKGDEVLVELAENGKKALVNKDDIQKMNPPKFSKVEDMAELTCLNEASVL

HNLKDRYYSGLIYTYSGLFCVVINPYKNLPIYSENIIEMYRGKKRHEMPPHIYAISES

AYRCMLQDREDQSILCTGESGAGKTENTKKVIQYLAHVASSHKGRKDHNIPPESPKPV

KHQGELERQLLQANPILESFGNAKTVKNDNSSRFGKFIRINFDVTGYIVGANIETYLL

EKSRAVRQAKDERTFHIFYQLLAGAGEHLKSDLLLEGFNNYRFLSNGYIPIPGQQDKD

NFQETMEAMHIMGFSHDEILSMLKVVSSVLQFGNISFKKERNTDQASMPENTVAQKLC

HLLGMNVMEFTRAILTPRIKVGRDYVQKAQTKEQADFAVEALAKATYERLFRWLVHRI

NKALDRTKRQGASFIGILDIAGFEIFELNSFEQLCINYTNEKLQQLFNHTMFILEQEE

YQREGIEWNFIDFGLDLQPCIDLIERPANPPGVLALLDEECWFPKATDKTFVEKLVQE

QGTHSKFQKPRQLKDKADFCIIHYAGKVDYKADEWLMKNMDPLNDNVATLLHQSSDKF

VAELWKDVDRIVGLDQVTGITETAFGSAYKTKKGMFRTVGQLYKESLTKLMATLRNTN

PNFVRCIIPNHEKRAGKLDPHLVLDQLRCNGVLEGIRICRQGFPNRIVFQEFRQRYEI

LTPNAIPKGFMDGKQACERMIRALELDPNLYRIGQSKIFFRAGVLAHLEEERDLKITD

IIIFFQAVCRGYLARKAFAKKQQQLSALKILQRNCAAYLKLRHWQWWRVFTKVKPLLQ

VTRQEEELQAKDEELMKVKEKQTKVEAELEEMERKHQQLLEEKNILAEQLQAETELFA

EAEEMRARLAAKKQELEEILHDLESRVEEEEERNQILQNEKKKMQGHIQDLEEQLDEE

EGARQKLQLEKVTAEAKIKKMEEEILLLEDQNSKFLKEKKLMEDRIAECTSQLAEEEE

KAKNLAKLKNKQEMMITDLEERLKKEEKTRQELEKAKRKLDGETTDLQDQIAELQAQI

EELKIQLAKKEEELQAALARGDEEAVQKNNALKVIRELQAQIAELQEDLESEKASRNK

AEKQKRDLSEELEALKTELEDTLDTTAAQQELRTKREQEVAELKKAIEEETKNHEAQI

QEIRQRHATALEELSEQLEQAKRFKANLEKNKQGLESDNKELACEVKVLQQVKAESEH

KRKKLDAQVQELTAKVTEGERLRVELAEKANKLQNELDNVSSLLEEAEKKGIKFAKDA

ASLESQLQDTQELLQEETRQKLNLSSRIRQLEEEKNNLQEQQEEEEEARKNLEKQMLA

LQSQLADAKKKVDDDLGTIEGLEENKKKLLKDMESLSQRLEEKAMAYDKLEKTKNRLQ

QELDDLMVDLDHQRQIVSNLEKKQKKFDQMLAEEKNISARYAEERDRAEAEAREKETK

ALSLARALEEALEAKEEFERQNKQLRADMEDLMSSKDDVGKNVHELEKSKRTLEQQVE

EMRTQLEELEDELQATEDAKLRLEVNMQAMKAQFERDLQARDEQNEEKKRMLVKQVRE

LEAELEDERKQRALAVAAKKKMEMDLKDLEGQIEAANKARDEAIKQLRKLQAQMKDYQ

RELEEARASRDEIFAQSKESEKKLKGLEAEILQLQEEFAASERARRHAEQERDELADE

IANSASGKSALLDEKRRLEARIAQLEEELEEEQSNMELLNERFRKTTLQVDTLNSELA

GERSAAQKSENARQQLERQNKELKAKLQELEGSVKSKFKATISTLEAKIVQLEEQLEQ

EAKERAAANKLVRRTEKKLKEVFMQVEDERRHADQYKEQMEKANARMKQLKRQLEEAE

EEATRANASRRKLQRELDDATEANEGLSREVSTLKNRLRRGGPITFSSSRSGRRQLHI

EGASLELSDDDAESKGSDVNEAQPAPAE
