# Supplementary material for: Proteomic Screening for Cellular Targets of the Duck Enteritis Virus Protein VP26 Reveals That the Host Actin–Myosin II Network Regulates the Proliferation of the Virus
Source: Int J Mol Sci. 2025 Sep 18;26(18):9108. doi: 10.3390/ijms26189108 (PMC12470233; doi:10.3390/ijms26189108)
Supplement: Supplementary file 1 [file ijms-26-09108-s001.zip › Supplement S4- Alignment of duck-original and chick-original protein sequences/MYH10.pdf]

```
                      10         20         30         40         50         60
              ....|....| ....|....| ....|....| ....|....| ....|....| ....|....|
chick MYH10   MAQRSGQEDP ERYLFVDRAV IYNPATQADW TAKKLVWIPS ERHGFEAASI KEERGDEVLV
duck MYH10    .......... .......... .......... .......... .......... ...K......

                      70         80         90        100        110        120
              ....|....| ....|....| ....|....| ....|....| ....|....| ....|....|
chick MYH10   ELAENGKKAL VNKDDIQKMN PPKFSKVEDM AELTCLNEAS VLHNLKDRYY SGLIYTYSGL
duck MYH10    .......... .......... .......... .......... .......... ..........

                     130        140        150        160        170        180
              ....|....| ....|....| ....|....| ....|....| ....|....| ....|....|
chick MYH10   FCVVINPYKN LPIYSENIIE MYRGKKRHEM PPHIYAISES AYRCMLQDRE DQSILCTGES
duck MYH10    .......... .......... .......... .......... .......... ..........

                     190        200        210        220        230        240
              ....|....| ....|....| ....|....| ....|....| ....|....| ....|....|
chick MYH10   GAGKTENTKK VIQYLAHVAS SHKGRKDHNI P--------- -GELERQLLQ ANPILESFGN
duck MYH10    .......... .......... .......... .PESPKPVKH Q......... ..........

                     250        260        270        280        290        300
              ....|....| ....|....| ....|....| ....|....| ....|....| ....|....|
chick MYH10   AKTVKNDNSS RFGKFIRINF DVTGYIVGAN IETYLLEKSR AVRQAKDERT FHIFYQLLAG
duck MYH10    .......... .......... .......... .......... .......... ..........

                     310        320        330        340        350        360
              ....|....| ....|....| ....|....| ....|....| ....|....| ....|....|
chick MYH10   AGEHLKSDLL LEGFNNYRFL SNGYIPIPGQ QDKDNFQETM EAMHIMGFSH DEILSMLKVV
duck MYH10    .......... .......... .......... .......... .......... ..........

                     370        380        390        400        410        420
              ....|....| ....|....| ....|....| ....|....| ....|....| ....|....|
chick MYH10   SSVLQFGNIS FKKERNTDQA SMPENTVAQK LCHLLGMNVM EFTRAILTPR IKVGRDYVQK
duck MYH10    .......... .......... .......... .......... .......... ..........

                     430        440        450        460        470        480
              ....|....| ....|....| ....|....| ....|....| ....|....| ....|....|
chick MYH10   AQTKEQADFA VEALAKATYE RLFRWLVHRI NKALDRTKRQ GASFIGILDI AGFEIFELNS
duck MYH10    .......... .......... .......... .......... .......... ..........

                     490        500        510        520        530        540
              ....|....| ....|....| ....|....| ....|....| ....|....| ....|....|
chick MYH10   FEQLCINYTN EKLQQLFNHT MFILEQEEYQ REGIEWNFID FGLDLQPCID LIERPANPPG
duck MYH10    .......... .......... .......... .......... .......... ..........

                     550        560        570        580        590        600
              ....|....| ....|....| ....|....| ....|....| ....|....| ....|....|
chick MYH10   VLALLDEECW FPKATDKTFV EKLVQEQGTH SKFQKPRQLK DKADFCIIHY AGKVDYKADE
duck MYH10    .......... .......... .......... .......... .......... ..........

                     610        620        630        640        650        660
              ....|....| ....|....| ....|....| ....|....| ....|....| ....|....|
chick MYH10   WLMKNMDPLN DNVATLLHQS SDKFVAELWK DVDRIVGLDQ VTGITETAFG SAYKTKKGMF
duck MYH10    .......... .......... .......... .......... .......... ..........

                     670        680        690        700        710        720
              ....|....| ....|....| ....|....| ....|....| ....|....| ....|....|
chick MYH10   RTVGQLYKES LTKLMATLRN TNPNFVRCII PNHEKRAGKL DPHLVLDQLR CNGVLEGIRI
duck MYH10    .......... .......... .......... .......... .......... ..........

                     730        740        750        760        770        780
              ....|....| ....|....| ....|....| ....|....| ....|....| ....|....|
chick MYH10   CRQGFPNRIV FQEFRQRYEI LTPNAIPKGF MDGKQACERM IRALELDPNL YRIGQSKIFF
duck MYH10    .......... .......... .......... .......... .......... ..........

                     790        800        810        820        830        840
              ....|....| ....|....| ....|....| ....|....| ....|....| ....|....|
chick MYH10   RAGVLAHLEE ERDLKITDII IFFQAVCRGY LARKAFAKKQ QQLSALKILQ RNCAAYLKLR
duck MYH10    .......... .......... .......... .......... .......... ..........

                     850        860        870        880        890        900
              ....|....| ....|....| ....|....| ....|....| ....|....| ....|....|
chick MYH10   HWQWWRVFTK VKPLLQVTRQ EEELQAKDEE LMKVKEKQTK VEAELEEMER KHQQLLEEKN
duck MYH10    .......... .......... .......... .......... .......... ..........

                     910        920        930        940        950        960
              ....|....| ....|....| ....|....| ....|....| ....|....| ....|....|
chick MYH10   ILAEQLQAET ELFAEAEEMR ARLAAKKQEL EEILHDLESR VEEEEERNQI LQNEKKKMQG
duck MYH10    .......... .......... .......... .......... .......... ..........

                     970        980        990       1000       1010       1020
              ....|....| ....|....| ....|....| ....|....| ....|....| ....|....|
chick MYH10   HIQDLEEQLD EEEGARQKLQ LEKVTAEAKI KKMEEEILLL EDQNSKFLKE KKLMEDRIAE
duck MYH10    .......... .......... .......... .......... .......... ..........

                    1030       1040       1050       1060       1070       1080
              ....|....| ....|....| ....|....| ....|....| ....|....| ....|....|
chick MYH10   CTSQLAEEEE KAKNLAKLKN KQEMMITDLE ERLKKEEKTR QELEKAKRKL DGETTDLQDQ
duck MYH10    .......... .......... .......... .......... .......... ..........

                    1090       1100       1110       1120       1130       1140
              ....|....| ....|....| ....|....| ....|....| ....|....| ....|....|
```

```
chick MYH10   IAELQAQIEE LKIQLAKKEE ELQAALARGD EEAVQKNNAL KVIRELQAQI AELQEDLESE
duck MYH10    .......... .......... .......... .......... .......... ..........

                    1150       1160       1170       1180       1190       1200
              ....|....| ....|....| ....|....| ....|....| ....|....| ....|....|
chick MYH10   KASRNKAEKQ KRDLSEELEA LKTELEDTLD TTAAQQELRT KREQEVAELK KAIEEETKNH
duck MYH10    .......... .......... .......... .......... .......... ..........

                    1210       1220       1230       1240       1250       1260
              ....|....| ....|....| ....|....| ....|....| ....|....| ....|....|
chick MYH10   EAQIQEIRQR HATALEELSE QLEQAKRFKA NLEKNKQGLE SDNKELACEV KVLQQVKAES
duck MYH10    .......... .......... .......... .......... .......... ..........

                    1270       1280       1290       1300       1310       1320
              ....|....| ....|....| ....|....| ....|....| ....|....| ....|....|
chick MYH10   EHKRKKLDAQ VQELTAKVTE GERLRVELAE KANKLQNELD NVSSLLEEAE KKGIKFAKDA
duck MYH10    .......... .......... .......... .......... .......... ..........

                    1330       1340       1350       1360       1370       1380
              ....|....| ....|....| ....|....| ....|....| ....|....| ....|....|
chick MYH10   ASLESQLQDT QELLQEETRQ KLNLSSRIRQ LEEEKNNLQE QQEEEEEARK NLEKQMLALQ
duck MYH10    .......... .......... .......... .......... .......... ..........

                    1390       1400       1410       1420       1430       1440
              ....|....| ....|....| ....|....| ....|....| ....|....| ....|....|
chick MYH10   AQLAEAKKKV DDDLGTIEGL EENKKKLLKD MESLSQRLEE KAMAYDKLEK TKNRLQQELD
duck MYH10    S...D..... .......... .......... .......... .......... ..........

                    1450       1460       1470       1480       1490       1500
              ....|....| ....|....| ....|....| ....|....| ....|....| ....|....|
chick MYH10   DLMVDLDHQR QIVSNLEKKQ KKFDQMLAEE KNISARYAEE RDRAEAEARE KETKALSLAR
duck MYH10    .......... .......... .......... .......... .......... ..........

                    1510       1520       1530       1540       1550       1560
              ....|....| ....|....| ....|....| ....|....| ....|....| ....|....|
chick MYH10   ALEEALEAKE EFERQNKQLR ADMEDLMSSK DDVGKNVHEL EKSKRTLEQQ VEEMRTQLEE
duck MYH10    .......... .......... .......... .......... .......... ..........

                    1570       1580       1590       1600       1610       1620
              ....|....| ....|....| ....|....| ....|....| ....|....| ....|....|
chick MYH10   LEDELQATED AKLRLEVNMQ AMKAQFERDL QARDEQNEEK KRMLVKQVRE LEAELEDERK
duck MYH10    .......... .......... .......... .......... .......... ..........

                    1630       1640       1650       1660       1670       1680
              ....|....| ....|....| ....|....| ....|....| ....|....| ....|....|
chick MYH10   QRALAVAAKK KMEMDLKDLE GQIEAANKAR DEAIKQLRKL QAQMKDYQRE LEEARASRDE
duck MYH10    .......... .......... .......... .......... .......... ..........

                    1690       1700       1710       1720       1730       1740
              ....|....| ....|....| ....|....| ....|....| ....|....| ....|....|
chick MYH10   IFAQSKESEK KLKGLEAEIL QLQEEFAASE RARRHAEQER DELADEIANS ASGKSALLDE
duck MYH10    .......... .......... .......... .......... .......... ..........

                    1750       1760       1770       1780       1790       1800
              ....|....| ....|....| ....|....| ....|....| ....|....| ....|....|
chick MYH10   KRRLEARIAQ LEEELEEEQS NMELLNERFR KTTLQVDTLN SELAGERSAA QKSENARQQL
duck MYH10    .......... .......... .......... .......... .......... ..........

                    1810       1820       1830       1840       1850       1860
              ....|....| ....|....| ....|....| ....|....| ....|....| ....|....|
chick MYH10   ERQNKELKAK LQELEGSVKS KFKATISTLE AKIAQLEEQL EQEAKERAAA NKLVRRTEKK
duck MYH10    .......... .......... .......... ...V...... .......... ..........

                    1870       1880       1890       1900       1910       1920
              ....|....| ....|....| ....|....| ....|....| ....|....| ....|....|
chick MYH10   LKEVFMQVED ERRHADQYKE QMEKANARMK QLKRQLEEAE EEATRANASR RKLQRELDDA
duck MYH10    .......... .......... .......... .......... .......... ..........

                    1930       1940       1950       1960       1970       1980
              ....|....| ....|....| ....|....| ....|....| ....|....| ....|....|
chick MYH10   TEANEGLSRE VSTLKNRLRR GGPITFSSSR SGRRQLHIEG ASLELSDDDA ESKGSDVNEA
duck MYH10    .......... .......... .......... .......... .......... ..........

              ....|.
chick MYH10   QPTPAE
duck MYH10    ..A...
```
