# Supplementary material for: Proteomic Screening for Cellular Targets of the Duck Enteritis Virus Protein VP26 Reveals That the Host Actin–Myosin II Network Regulates the Proliferation of the Virus
Source: Int J Mol Sci. 2025 Sep 18;26(18):9108. doi: 10.3390/ijms26189108 (PMC12470233; doi:10.3390/ijms26189108)
Supplement: Supplementary file 1 [file ijms-26-09108-s001.zip › Supplement S4- Alignment of duck-original and chick-original protein sequences/MYH9.file.pdf]

https://www.ncbi.nlm.nih.gov/nuccore/2925828650

PREDICTED: Anas platyrhynchos myosin heavy chain 9 (MYH9), transcript variant X1, mRNA

>duck MYH9

MAQRDADKYLYVDKNIINNPLTQADWAAKKLVWVPSEKNGFEAA

SLKEEVGDEAIVELAENGKKVKVNKDDIQKMNPPKFSKVEDMAELTCLNEASVLHNLK

ERYYSGLIYTYSGLFCVVINPYKNLPIYSEEIVEMYKGKKRHEMPPHIYAITDTAYRS

MMQDREDQSILCTGESGAGKTENTKKVIQYLAHVASSHKSKKDQGELERQLLQANPIL

EAFGNAKTVKNDNSSRFGKFIRINFDVNGYIVGANIETYLLEKSRAIRQAKEERTFHI

FYYLLSGAGEHLKTDLLLEPYNKYRFLSNGHVTIPGQQDKDMFQETMEAMRIMGIPDE

EQIGLLKVISGVLQLGNIVFKKERNTDQASMPDNTAAQKVSHLLGINVTDFTRGILTP

RIKVGRDYVQKAQTKEQADFAIEALAKATYERMFRWLVMRINKALDKTKRQGASFIGI

LDIAGFEIFELNSFEQLCINYTNEKLQQLFNHTMFILEQEEYQREGIEWNFIDFGLDL

QPCIDLIEKPAGPPGILALLDEECWFPKATDKSFVEKVVQEQGTHPKFQKPKQLKDKA

DFCIIHYAGKVDYKADEWLMKNMDPLNDNIATLLHQSSDKFVSELWKDVDRIVGLDQV

AGMSETALPGAFKTRKGMFRTVGQLYKEQLAKLMATLRNTNPNFVRCIIPNHEKKAGK

LDPHLVLDQLRCNGVLEGIRICRQGFPNRVVFQEFRQRYEILTPNAIPKGFMDGKQAC

VLMIKALELDSNLYRIGQSKVFFRAGVLAHLEEERDLKITDVIIGFQACCRGYLARKA

FAKRQQQLTAMKVLQRNCAAYLKLRNWQWWRLFTKVKPLLQVSRQEEEMMAKEEELIK

VKEKQLAAENRLSEMETFQAQLMAEKMQLQEQLQAETELCAEAEEIRARLTAKKQELE

EICHDLEARVEEEEEERCQHLQAEKKKMQQNIQELEEQLEEEESTRQKLQLEKVTTEAK

LKKLEEHMIVLEDQNLKLAKEKKLLEDRMSEFTTNLTEEEEKSKSLAKLKNKHEAMIT

DLEERLRREEKQRQELEKTRRKLEGDSTDLHDQIAELQAQIAELKMQLAKKEEELQAA

LARVEEEAAQKNMALKKIRELESQITELQEDLESERAFRNKAEKQKRDLGEELEALKT

ELEDTLDSTAAQQELRSKREQEVTVLKKTLEDEAKTHEAQIQEMRQKHSQAIEELAEQ

LEQTKRVKANLEKAKQALESERAELSNEVKVLLQGKGDAEHKRKKVDAQLQELQVKFT

EGERVKAELADKVNKLQVELDNVTGLLNQSDSKSIKLAKDFSALESQLQDTQELLQEE

TRLKLSFSTRLKQTEDEKNALREQLEEEEEAKKNLEKQISILQQQAIEAKKKMDDGLG

CLESAEEARKKLQKDLEGLNQRYEEKIAAYDKLEKTKTRLQQELDDITVDLDHQRQTV

SNLEKKQKKFDQLLAEEKNISAKYAEERDRAEAEAREKETKALSLARALEEAIEQKAE

LERVNKQFRTEMEDLMSSKDDVGKSVHELEKAKRALEQQVEEMKTQLEELEDELQATE

DAKLRLEVNQQAMKAQFDRDLQGRDEQNEEKRKQLIRQVREMEVELEDERKQRSIAMA

ARKKLELDLKDLESHIDTANKNREEAIKQLRKLQAQMKDYMRELEDTRTSREEILAQA

KENEKKLKSMEAEMIQLQEELAAAERAKRQAQQERDELADEIANSSGKGALAMEEKRR

LEARIAQLEEELEEEQGNTEIINDRLKKANLQIDQMNADLNAERSNAQKNENARQQME

RQNKELKLKLQEMESAVKSKYKATITALEAKIVQLEEQLDMETKERQAASKQVRRAEK

KLKDILLQVDDERRNAEQFKDQADKANMRLKQLKRQLEEAEEEAQRANASRRKLQREL

EDATETADAMNREVSSLKSKLRRGDLPFVVTRRIVRKGTTGECSDEEVDGKADAGDAK

ATE

https://www.uniprot.org/uniprotkb/A0A1D5PM19/entry

> Chick MYH9

MAQRDADKYLYVDKNIINNPLTQADWAAKKLVWVPSEKSGFEAASLKEEVGDEAIVELAENG
KKVKVNKDDIQKMNPPKFSKVEDMAELTCLNEASVLHNLKERYYSGLIYTYSGLFCVVINPY
KNLPIYSEEIVEMYKGKKRHEMPPHIYAITDTAYRSMMQDREDQSILCTGESGAGKTENTKK
VIQYLAHVASSHKSKKDQGELERQLLQANPILEAFGNAKTVKNDNSSRFGKFIRINFDVNGY
IVGANIETYLLEKSRAIRQAKEERTFHIFYYLLSGAGEHLKTDLLLEPYNKYRFLSNGHVTI
PGQQDKDMFQETMEAMRIMGIPDEEQIGLLKVISGVLQLGNIVFKKERNTDQASMPDNTAAQ
KVSHLLGINVTDFTRGILTPRIKVGRDYVQKAQTKEQADFAIEALAKATYERMFRWLVMRIN
KALDKTKRQGASFIGILDIAGFEIFELNSFEQLCINYTNEKLQQLFNHTMFILEQEEYQREG
IEWNFIDFGLDLQPCIDLIEKPAGPPGILALLDEECWFPKATDKSFVEKVVQEQGTHPKFQK
PKQLKDKADFCIIHYAGKVDYKADEWLMKNMDPLNDNIATLLHQSSDKFVSELWKDVDRIVG
LDQVAGMSETALPGAFKTRKGMFRTVGQLYKEQLAKLMATLRNTNPNFVRCIIPNHEKKAGK
LDPHLVLDQLRCNGVLEGIRICRQGFPNRVVFQEFRQRYEILTPNAIPKGFMDGKQACVLMI
KALELDSNLYRIGQSKVFFRAGVLAHLEEERDLKITDVIIGFQACCRGYLARKAFAKRQQQL
TAMKVLQRNCAAYLKLRNWQWWRLFTKVKPLLQVSRQEEEMMAKEEELIKVKEKQLAAENRL
SEMETFQAQLMAEKMQLQEQLQAEAELCAEAEEIRARLTAKKQELEEICHDLEARVEEEEER
CQHLQAEKKKMQQNIQELEEQLEEEESARQKLQLEKVTTEAKLKKLEEDVIVLEDQNLKLAK
EKKLLEDRMSEFTTNLTEEEEKSKSLAKLKNKHEAMITDLEERLRREEKQRQELEKTRRKLE
GDSSDLHDQIAELQAQIAELKIQLSKKEEELQAALARVEEEAAQKNMALKKIRELESQITEL
QEDLESERASRNKAEKQKRDLGEELEALKTELEDTLDSTAAQQELRSKREQEVTVLKKTLED
EAKTHEAQIQEMRQKHSQAIEELAEQLEQTKRVKANLEKAKQALESERAELSNEVKVLLQGK
GDAEHKRKKVDAQLQELQVKFTEGERVKTELAERVNKLQVELDNVTGLLNQSDSKSIKLAKD
FSALESQLQDTQELLQEETRLKLSFSTKLKQTEDEKNALKEQLEEEEEAKRNLEKQISVLQQ
QAVEARKKMDDGLGCLEIAEEAKKKLQKDLESLTQRYEEKIAAYDKLEKTKTRLQQELDDIA
VDLDHQRQTVSNLEKKQKKFDQLLAEEKNISAKYAEERDRAEAEAREKETKALSLARALEEA
IEQKAELERVNKQFRTEMEDLMSSKDDVGKSVHELEKAKRALEQQVEEMKTQLEELEDELQA
TEDAKLRLEVNQQAMKAQFDRDLLGRDEQNEEKRKQLIRQVREMEVELEDERKQRSIAVAAR
KKLELDLKDLESHIDTANKNRDEAIKQLRKLQAQMKDYMRELEDTRTSREEILAQAKENEKK
LKSMEAEMIQLQEELAAAERAKRQAQQERDELADEIANSSGKGALAMEEKRRLEARIAQLEE
ELEEEQGNTEIINDRLKKANLQIDQMNADLNAERSNAQKNENARQQMERQNKELKLKLQEME
SAVKSKYKATITALEAKIVQLEEQLDMETKERQAASKQVRRAEKKLKDILLQVDDERRNAEQ
FKDQADKANMRLKQLKRQLEEAEEEAQRANASRRKLQRELDDATETADAMNREVSSLKSKLR
RGDLPFVVTRRLVRKGTGECSDEEVDGKAEAGDAKATE
