# Supplementary material for: Proteomic Screening for Cellular Targets of the Duck Enteritis Virus Protein VP26 Reveals That the Host Actin–Myosin II Network Regulates the Proliferation of the Virus
Source: Int J Mol Sci. 2025 Sep 18;26(18):9108. doi: 10.3390/ijms26189108 (PMC12470233; doi:10.3390/ijms26189108)
Supplement: Supplementary file 1 [file ijms-26-09108-s001.zip › Supplement S4- Alignment of duck-original and chick-original protein sequences/MYH9.pdf]

```
                    10         20         30         40         50         60
            ....|....| ....|....| ....|....| ....|....| ....|....| ....|....|
duck MYH9   MAQRDADKYL YVDKNIINNP LTQADWAAKK LVWVPSEKNG FEAASLKEEV GDEAIVELAE
chick MYH9  .......... .......... .......... ........S. .......... ..........

                    70         80         90        100        110        120
            ....|....| ....|....| ....|....| ....|....| ....|....| ....|....|
duck MYH9   NGKKVKVNKD DIQKMNPPKF SKVEDMAELT CLNEASVLHN LKERYYSGLI YTYSGLFCVV
chick MYH9  .......... .......... .......... .......... .......... ..........

                   130        140        150        160        170        180
            ....|....| ....|....| ....|....| ....|....| ....|....| ....|....|
duck MYH9   INPYKNLPIY SEEIVEMYKG KKRHEMPPHI YAITDTAYRS MMQDREDQSI LCTGESGAGK
chick MYH9  .......... .......... .......... .......... .......... ..........

                   190        200        210        220        230        240
            ....|....| ....|....| ....|....| ....|....| ....|....| ....|....|
duck MYH9   TENTKKVIQY LAHVASSHKS KKDQGELERQ LLQANPILEA FGNAKTVKND NSSRFGKFIR
chick MYH9  .......... .......... .......... .......... .......... ..........

                   250        260        270        280        290        300
            ....|....| ....|....| ....|....| ....|....| ....|....| ....|....|
duck MYH9   INFDVNGYIV GANIETYLLE KSRAIRQAKE ERTFHIFYYL LSGAGEHLKT DLLLEPYNKY
chick MYH9  .......... .......... .......... .......... .......... ..........

                   310        320        330        340        350        360
            ....|....| ....|....| ....|....| ....|....| ....|....| ....|....|
duck MYH9   RFLSNGHVTI PGQQDKDMFQ ETMEAMRIMG IPDEEQIGLL KVISGVLQLG NIVFKKERNT
chick MYH9  .......... .......... .......... .......... .......... ..........

                   370        380        390        400        410        420
            ....|....| ....|....| ....|....| ....|....| ....|....| ....|....|
duck MYH9   DQASMPDNTA AQKVSHLLGI NVTDFTRGIL TPRIKVGRDY VQKAQTKEQA DFAIEALAKA
chick MYH9  .......... .......... .......... .......... .......... ..........

                   430        440        450        460        470        480
            ....|....| ....|....| ....|....| ....|....| ....|....| ....|....|
duck MYH9   TYERMFRWLV MRINKALDKT KRQGASFIGI LDIAGFEIFE LNSFEQLCIN YTNEKLQQLF
chick MYH9  .......... .......... .......... .......... .......... ..........

                   490        500        510        520        530        540
            ....|....| ....|....| ....|....| ....|....| ....|....| ....|....|
duck MYH9   NHTMFILEQE EYQREGIEWN FIDFGLDLQP CIDLIEKPAG PPGILALLDE ECWFPKATDK
chick MYH9  .......... .......... .......... .......... .......... ..........

                   550        560        570        580        590        600
            ....|....| ....|....| ....|....| ....|....| ....|....| ....|....|
duck MYH9   SFVEKVVQEQ GTHPKFQKPK QLKDKADFCI IHYAGKVDYK ADEWLMKNMD PLNDNIATLL
chick MYH9  .......... .......... .......... .......... .......... ..........

                   610        620        630        640        650        660
            ....|....| ....|....| ....|....| ....|....| ....|....| ....|....|
duck MYH9   HQSSDKFVSE LWKDVDRIVG LDQVAGMSET ALPGAFKTRK GMFRTVGQLY KEQLAKLMAT
chick MYH9  .......... .......... .......... .......... .......... ..........

                   670        680        690        700        710        720
            ....|....| ....|....| ....|....| ....|....| ....|....| ....|....|
duck MYH9   LRNTNPNFVR CIIPNHEKKA GKLDPHLVLD QLRCNGVLEG IRICRQGFPN RVVFQEFRQR
chick MYH9  .......... .......... .......... .......... .......... ..........

                   730        740        750        760        770        780
            ....|....| ....|....| ....|....| ....|....| ....|....| ....|....|
duck MYH9   YEILTPNAIP KGFMDGKQAC VLMIKALELD SNLYRIGQSK VFFRAGVLAH LEEERDLKIT
chick MYH9  .......... .......... .......... .......... .......... ..........

                   790        800        810        820        830        840
            ....|....| ....|....| ....|....| ....|....| ....|....| ....|....|
duck MYH9   DVIIGFQACC RGYLARKAFA KRQQQLTAMK VLQRNCAAYL KLRNWQWWRL FTKVKPLLQV
chick MYH9  .......... .......... .......... .......... .......... ..........

                   850        860        870        880        890        900
            ....|....| ....|....| ....|....| ....|....| ....|....| ....|....|
duck MYH9   SRQEEEMMAK EEELIKVKEK QLAAENRLSE METFQAQLMA EKMQLQEQLQ AETELCAEAE
chick MYH9  .......... .......... .......... .......... .......... ..A.......

                   910        920        930        940        950        960
            ....|....| ....|....| ....|....| ....|....| ....|....| ....|....|
duck MYH9   EIRARLTAKK QELEEICHDL EARVEEEEER CQHLQAEKKK MQQNIQELEE QLEEEESTRQ
chick MYH9  .......... .......... .......... .......... .......... .......A..

                   970        980        990       1000       1010       1020
            ....|....| ....|....| ....|....| ....|....| ....|....| ....|....|
duck MYH9   KLQLEKVTTE AKLKKLEEHM IVLEDQNLKL AKEKKLLEDR MSEFTTNLTE EEEKSKSLAK
chick MYH9  .......... ........DV .......... .......... .......... ..........

                  1030       1040       1050       1060       1070       1080
            ....|....| ....|....| ....|....| ....|....| ....|....| ....|....|
duck MYH9   LKNKHEAMIT DLEERLRREE KQRQELEKTR RKLEGDSTDL HDQIAELQAQ IAELKMQLAK
chick MYH9  .......... .......... .......... .......S.. .......... .....I..S.

                  1090       1100       1110       1120       1130       1140
            ....|....| ....|....| ....|....| ....|....| ....|....| ....|....|
```

```
duck MYH9     KEEELQAALA RVEEEAAQKN MALKKIRELE SQITELQEDL ESERAFRNKA EKQKRDLGEE
chick MYH9    .......... .......... .......... .......... .....S.... ..........

                       1150       1160       1170       1180       1190       1200
              ....|....| ....|....| ....|....| ....|....| ....|....| ....|....|
duck MYH9     LEALKTELED TLDSTAAQQE LRSKREQEVT VLKKTLEDEA KTHEAQIQEM RQKHSQAIEE
chick MYH9    .......... .......... .......... .......... .......... ..........

                       1210       1220       1230       1240       1250       1260
              ....|....| ....|....| ....|....| ....|....| ....|....| ....|....|
duck MYH9     LAEQLEQTKR VKANLEKAKQ ALESERAELS NEVKVLLQGK GDAEHKRKKV DAQLQELQVK
chick MYH9    .......... .......... .......... .......... .......... ..........

                       1270       1280       1290       1300       1310       1320
              ....|....| ....|....| ....|....| ....|....| ....|....| ....|....|
duck MYH9     FTEGERVKAE LADKVNKLQV ELDNVTGLLN QSDSKSIKLA KDFSALESQL QDTQELLQEE
chick MYH9    ........T. ..ER...... .......... .......... .......... ..........

                       1330       1340       1350       1360       1370       1380
              ....|....| ....|....| ....|....| ....|....| ....|....| ....|....|
duck MYH9     TRLKLSFSTR LKQTEDEKNA LREQLEEEEE AKKNLEKQIS ILQQQAIEAK KKMDDGLGCL
chick MYH9    .........K .......... .K........ ..R....... V.....V..R ..........

                       1390       1400       1410       1420       1430       1440
              ....|....| ....|....| ....|....| ....|....| ....|....| ....|....|
duck MYH9     ESAEEARKKL QKDLEGLNQR YEEKIAAYDK LEKTKTRLQQ ELDDITVDLD HQRQTVSNLE
chick MYH9    .I....K... .....S.T.. .......... .......... .....A.... ..........

                       1450       1460       1470       1480       1490       1500
              ....|....| ....|....| ....|....| ....|....| ....|....| ....|....|
duck MYH9     KKQKKFDQLL AEEKNISAKY AEERDRAEAE AREKETKALS LARALEEAIE QKAELERVNK
chick MYH9    .......... .......... .......... .......... .......... ..........

                       1510       1520       1530       1540       1550       1560
              ....|....| ....|....| ....|....| ....|....| ....|....| ....|....|
duck MYH9     QFRTEMEDLM SSKDDVGKSV HELEKAKRAL EQQVEEMKTQ LEELEDELQA TEDAKLRLEV
chick MYH9    .......... .......... .......... .......... .......... ..........

                       1570       1580       1590       1600       1610       1620
              ....|....| ....|....| ....|....| ....|....| ....|....| ....|....|
duck MYH9     NQQAMKAQFD RDLQGRDEQN EEKRKQLIRQ VREMEVELED ERKQRSIAMA ARKKLELDLK
chick MYH9    .......... ...L...... .......... .......... ........V. ..........

                       1630       1640       1650       1660       1670       1680
              ....|....| ....|....| ....|....| ....|....| ....|....| ....|....|
duck MYH9     DLESHIDTAN KNREEAIKQL RKLQAQMKDY MRELEDTRTS REEILAQAKE NEKKLKSMEA
chick MYH9    .......... ...D...... .......... .......... .......... ..........

                       1690       1700       1710       1720       1730       1740
              ....|....| ....|....| ....|....| ....|....| ....|....| ....|....|
duck MYH9     EMIQLQEELA AAERAKRQAQ QERDELADEI ANSSGKGALA MEEKRRLEAR IAQLEEELEE
chick MYH9    .......... .......... .......... .......... .......... ..........

                       1750       1760       1770       1780       1790       1800
              ....|....| ....|....| ....|....| ....|....| ....|....| ....|....|
duck MYH9     EQGNTEIIND RLKKANLQID QMNADLNAER SNAQKNENAR QQMERQNKEL KLKLQEMESA
chick MYH9    .......... .......... .......... .......... .......... ..........

                       1810       1820       1830       1840       1850       1860
              ....|....| ....|....| ....|....| ....|....| ....|....| ....|....|
duck MYH9     VKSKYKATIT ALEAKIVQLE EQLDMETKER QAASKQVRRA EKKLKDILLQ VDDERRNAEQ
chick MYH9    .......... .......... .......... .......... .......... ..........

                       1870       1880       1890       1900       1910       1920
              ....|....| ....|....| ....|....| ....|....| ....|....| ....|....|
duck MYH9     FKDQADKANM RLKQLKRQLE EAEEEAQRAN ASRRKLQREL EDATETADAM NREVSSLKSK
chick MYH9    .......... .......... .......... .......... D......... ..........

                       1930       1940       1950       1960
              ....|....| ....|....| ....|....| ....|....| .
duck MYH9     LRRGDLPFVV TRRIVRKGTT GECSDEEVDG KADAGDAKAT E
chick MYH9    .......... ...L....-. .......... ..E....... .
```
