# Supplementary material for: Proteomic Screening for Cellular Targets of the Duck Enteritis Virus Protein VP26 Reveals That the Host Actin–Myosin II Network Regulates the Proliferation of the Virus
Source: Int J Mol Sci. 2025 Sep 18;26(18):9108. doi: 10.3390/ijms26189108 (PMC12470233; doi:10.3390/ijms26189108)
Supplement: Supplementary file 1 [file ijms-26-09108-s001.zip › Supplement S4- Alignment of duck-original and chick-original protein sequences/MYO5A.file.pdf]

https://www.uniprot.org/uniprotkb/Q02440/entry

>chick **MYO5A**

MAASELYTKYARVWIPDPEEVWKSAELLKDYKPGDKVLQLRLEEGKDLEYCLDPKTKELPPLRNPDILVGE
NDLTALSYLHEPAVLHNLKVRFIDSKLIYTYCGIVLVAINPYEQLPIYGEDIINAYSGQNMGDMDPHIFAV
AEEAYKQMARDERNQSIIVSGESGAGKTVSAKYAMRYFATVSGSASEANVEEKVLASNPIMESIGNAKTTR
NDNSSRFGKYIEIGFDKRYRIIGANMRTYLLEKSRVVFQAEEERNYHIFYQLCASAALPEFKTLRLGNANY
FHYTKQGGSPVIDGIDDAKEMVNTRQACTLLGISDSYQMGIFRILAGILHLGNVEFASRDSDSCAIPPKHD
PLTIFCDLMGVDYEEMAHWLCHRKLATATETYIKPISKLHAINARDALAKHIYANLFNWIVDHVNKALHST
VKQHSFIGVLDIYGFETFEINSFEQFCINYANEKLQQQFNMHVFKLEQEEYMKEQIPWTLIDFYDNQPCIN
LIEAKMGVLDLLDEECKMPKGSDDTWAQKLYNTHLNKCALFEKPRLSNKAFIIKHFADKVEYQCEGFLEKN
KDTVYEEQIKVLKSSKKFKLLPELFQDEEKAISPTSATPSGRVPLSRTPVKPAKARPGQTSKEHKKTVGHQ
FRNSLHLLMETLNATTPHYVRCIKPNDFKFPFTFDEKRAVQQLRACGVLETIRISAAGFPSRWTYQEFFSR
YRVLMKQKDVLSDRKQTCKNVLEKLILDKDKYQFGKTKIFFRAGQVAYLEKIRADKLRAACIRIQKTIRGW
LMRKKYMRMRRAAITIQRYVRGHQARCYATFLRRTRAAIIIQKFQRMYVVRKRYQCMRDATIALQALLRGY
LVRNKYQMMLREHKSIIIQKHVRGWLARVHYHRTLKAIVYLQCCYRRMMAKRELKKLKIEARSVERYKKLH
IGLENKIMQLQRKIDEQNKEYKSLLEKMNNLEITYSTETEKLRSDVERLRMSEEEAKNATNRVLSLQEEIA
KLRKELHQTQTEKKTIEEWADKYKHETEQLVSELKEQNTLLKTEKEELNRRIHDQAKEITETMEKKLVEET
KQLELDLNDERLRYQNLLNEFSRLEERYDDLKDEMNLMVSIPKPGHKRTDSTHSSNESEYTFSSEITEAED
LPLRMEEPSEKKAPLDMSLFLKLQKRVTELEQEKQSLQDELDRKEEQALRAKAKEEERPPIRGAELEYESL
KRQELESENKKLKNELNELQKALTETRAPEVTAPGAPAYRVLLDQLTSVSEELEVRKEEVLILRSQLVSQK
EAIQPKEDKNTMTDSTILLEDVQKMKDKGEIAQAYIGLKETNRLLESQLQSQKKSHENELESLRGEIQSLK
EENNRQQQLLAQNLQLPPEARIEASLQHEITRLTNENLDLMEQLEKQDKTVRKLKKQLKVFAKKIGELEVG
QMENISPGQIIDEPIRPVNIPRKEKDFQGMLEYKKEDEQKLVKNLILELKPRGVAVNLIPGLPAYILFMCV
RHADYLNDDQKVRSLLTSTINGIKKVLKKRGDDFETVSFWLSNTCRFLHCLKQYSGEEGFMKHNTPRQNEH
CLTNFDLAEYRQVLSDLAIQIYQQLVRVLENILQPMIVSGMLEHETIQGVSGVKPTGLRKRTSSIADEGTY
TLDSIIRQLNSFHSVMCQHGMDPELIKQVVKQMFYIIGAVTLNNLLLRKDMCSWSKGMQIRYNVSQLEEWL
RDKNLMNSGAKETLEPLIQAAQLLQVKKKTDEDAEAICSMCNALTTAQIVKVLNLYTPVNEFEERVLVSFI
RTIQLRLRDRKDSPQLLMDAKHIFPVTFPFNPSSLALETIQIPASLGLGFISRV

https://www.ncbi.nlm.nih.gov/gene/101798412

https://www.ncbi.nlm.nih.gov/nuccore/XM_072043547.1

>duck MYO5A

MAASELYTKYARVWIPDPEEVWKSAELLKDYKPGDKVLQLRLEE

GKDLEYCLDPKTKELPPLRNPDILVGENDLTALSYLHEPAVLHNLKVRFIDSKLIYTY

CGIVLVAINPYEQLPIYGEDIINAYSGQNMGDMDPHIFAVAEEAYKQMARDERNQSII

VSGESGAGKTVSAKYAMRYFATVSGSASEANVEEKVLASNPIMESIGNAKTTRNDNSS

RFGKYIEIGFDKRYRIIGANMRTYLLEKSRVVFQAEEERNYHIFYQLCASAALPEFKT

LRLGNANYFHYTKQGGSPVIDGVDDAKEMVNTRQACTLLGISDSYQMGIFRILAGILH

LGNVEFASRDSDSCAIPPKHDPLTIFCDLMGVEYEEMAHWLCHRKLATATETYIKPIS

KLHAINARDALAKHIYANLFNWIVDHVNKALHSTVKQHSFIGVLDIYGFETFEINSFE

QFCINYANEKLQQQFNMHVFKLEQEEYMKEQIPWTLIDFYDNQPCINLIEAKMGILDL

LDEECKMPKGSDDTWAQKLYNTHLNKCALFEKPRLSNKAFIIKHFADKVEYQCEGFLE

KNKDTVYEEQIKVLKSSKFKLLPELFQDEEKVLSPTSATPSGRVPLSRTAVKPAKARP

GQTSKEHKKTVGHQFRNSLHLLMETLNATTPHYVRCIKPNDFKFPFTFDEKRAVQQLR

ACGVLETIRISAAGFPSRWTYQEFFSRYRVLMKQRDVLGDRKQTCKNVLEKLILDKDK

YQFGKTKIFFRAGQVAYLEKIRADKLRAACIRIQKTIRGWLMRKKYVRMRKAAITIQR

YVRGYQARCYATFLRRTRAAITIQKFQRMYVVRKRYQCMRDATIALQALLRGYMARNK

YQMMLREHKSIIIQKHVRGWLARVHYRRTLKAIVYLQCCYRRMMAKRELKKLKIEARS

VERYKKLHIGLENKIMQLQRKIDEQNKEYKSLLEKLNSLEITYSTETERLRSDVERLR

MSEEEAKNATNRVLSLQEEIAKLRKELHQTQSEKKTIEEWADKYKHETEQLVSELKDQ

NTLLKTEKEELNRRIHDQAKEITEAMEKKLVEETKQLELDLNDERLRYQNLLNEFSRL

EERYDDLKDEMNLMVSIPKPGHKRTDSTHSSNESEYTFSSEITEAEDLPLRMEQEPSE

KKAPLDMSLFLKLQKRVTELEQEKQSLQDELDRKEEQALRAKAKEEERPPIRGAELEY

ESLKRQELESENKKLKNELNELQKALTETRSPEVTAPGAPAYRVLLDQLTSVSEELEV

RKEEVLILRSQLVSQKEAIQPKNTMTDSTILLEDVQKMKDKGEIAQAYIGLKETNRQS

PQDYHMLNEDGELWLVYEGLKQANRLLESQLQSQKKSHENELESLRGEIQSLKEENNR

QQQLLAQNLQLPPEARIEASLQHEITRLTNENLFYEELYADDPKKYQSYRISLYKRMI

DLMEQLEKQDKTVRKLKKQLKVFAKKIGELEVGQMENISPGQIIDEPIRPVNIPRKEK

DFQGMLEYKKEDEQKLVKNLILELKPRGVAVNLIPGLPAYILFMCVRHADYLNDDQKV

RSLLTSTINGIKKVLKKRGDDFETVSFWLSNTCRFLHCLKQYSGEEGFMKHNTPRQNE

HCLTNFDLAEYRQVLSDLAIQIYQQLVRVLENILQPMIVSGMLEHETIQGVSGVKPTG

LRKRTSSIADEGTYTLDSIIRQLNSFHSVMCQHGMDPELIKQVVKQMFYIIGAVTLNN

LLLRKDMCSWSKGMQIRYNVSQLEEWLRDKNLMNSGAKETLEPLIQAAQLLQVKKKTD

EDAEAICSMCNALTTAQIVKVLNLYTPVNEFEERVLVSFIRTIQLRLRDRKDSPQLLM
DAKHIFPVTFPFNPSSLALETIQIPASLGLGFISRV
