# Supplementary material for: Proteomic Screening for Cellular Targets of the Duck Enteritis Virus Protein VP26 Reveals That the Host Actin–Myosin II Network Regulates the Proliferation of the Virus
Source: Int J Mol Sci. 2025 Sep 18;26(18):9108. doi: 10.3390/ijms26189108 (PMC12470233; doi:10.3390/ijms26189108)
Supplement: Supplementary file 1 [file ijms-26-09108-s001.zip › Supplement S4- Alignment of duck-original and chick-original protein sequences/MYO5A.pdf]

```
                        10         20         30         40         50         60
                ....|....| ....|....| ....|....| ....|....| ....|....| ....|....|
chick MYO5A     MAASELYTKY ARVWIPDPEE VWKSAELLKD YKPGDKVLQL RLEEGKDLEY CLDPKTKELP
duck MYO5A      .......... .......... .......... .......... .......... ..........

                        70         80         90        100        110        120
                ....|....| ....|....| ....|....| ....|....| ....|....| ....|....|
chick MYO5A     PLRNPDILVG ENDLTALSYL HEPAVLHNLK VRFIDSKLIY TYCGIVLVAI NPYEQLPIYG
duck MYO5A      .......... .......... .......... .......... .......... ..........

                       130        140        150        160        170        180
                ....|....| ....|....| ....|....| ....|....| ....|....| ....|....|
chick MYO5A     EDIINAYSGQ NMGDMDPHIF AVAEEAYKQM ARDERNQSII VSGESGAGKT VSAKYAMRYF
duck MYO5A      .......... .......... .......... .......... .......... ..........

                       190        200        210        220        230        240
                ....|....| ....|....| ....|....| ....|....| ....|....| ....|....|
chick MYO5A     ATVSGSASEA NVEEKVLASN PIMESIGNAK TTRNDNSSRF GKYIEIGFDK RYRIIGANMR
duck MYO5A      .......... .......... .......... .......... .......... ..........

                       250        260        270        280        290        300
                ....|....| ....|....| ....|....| ....|....| ....|....| ....|....|
chick MYO5A     TYLLEKSRVV FQAEEERNYH IFYQLCASAA LPEFKTLRLG NANYFHYTKQ GGSPVIDGID
duck MYO5A      .......... .......... .......... .......... .......... ........V.

                       310        320        330        340        350        360
                ....|....| ....|....| ....|....| ....|....| ....|....| ....|....|
chick MYO5A     DAKEMVNTRQ ACTLLGISDS YQMGIFRILA GILHLGNVEF ASRDSDSCAI PPKHDPLTIF
duck MYO5A      .......... .......... .......... .......... .......... ..........

                       370        380        390        400        410        420
                ....|....| ....|....| ....|....| ....|....| ....|....| ....|....|
chick MYO5A     CDLMGVDYEE MAHWLCHRKL ATATETYIKP ISKLHAINAR DALAKHIYAN LFNWIVDHVN
duck MYO5A      ......E... .......... .......... .......... .......... ..........

                       430        440        450        460        470        480
                ....|....| ....|....| ....|....| ....|....| ....|....| ....|....|
chick MYO5A     KALHSTVKQH SFIGVLDIYG FETFEINSFE QFCINYANEK LQQQFNMHVF KLEQEEYMKE
duck MYO5A      .......... .......... .......... .......... .......... ..........

                       490        500        510        520        530        540
                ....|....| ....|....| ....|....| ....|....| ....|....| ....|....|
chick MYO5A     QIPWTLIDFY DNQPCINLIE AKMGVLDLLD EECKMPKGSD DTWAQKLYNT HLNKCALFEK
duck MYO5A      .......... .......... ....I..... .......... .......... ..........

                       550        560        570        580        590        600
                ....|....| ....|....| ....|....| ....|....| ....|....| ....|....|
chick MYO5A     PRLSNKAFII KHFADKVEYQ CEGFLEKNKD TVYEEQIKVL KSSKKFKLLP ELFQDEEKAI
duck MYO5A      .......... .......... .......... .......... ....-..... ........VL

                       610        620        630        640        650        660
                ....|....| ....|....| ....|....| ....|....| ....|....| ....|....|
chick MYO5A     SPTSATPSGR VPLSRTPVKP AKARPGQTSK EHKKTVGHQF RNSLHLLMET LNATTPHYVR
duck MYO5A      .......... ......A... .......... .......... .......... ..........

                       670        680        690        700        710        720
                ....|....| ....|....| ....|....| ....|....| ....|....| ....|....|
chick MYO5A     CIKPNDFKFP FTFDEKRAVQ QLRACGVLET IRISAAGFPS RWTYQEFFSR YRVLMKQKDV
duck MYO5A      .......... .......... .......... .......... .......... .......R..

                       730        740        750        760        770        780
                ....|....| ....|....| ....|....| ....|....| ....|....| ....|....|
chick MYO5A     LSDRKQTCKN VLEKLILDKD KYQFGKTKIF FRAGQVAYLE KIRADKLRAA CIRIQKTIRG
duck MYO5A      .G........ .......... .......... .......... .......... ..........

                       790        800        810        820        830        840
                ....|....| ....|....| ....|....| ....|....| ....|....| ....|....|
chick MYO5A     WLMRKKYMRM RRAAITIQRY VRGHQARCYA TFLRRTRAAI IIQKFQRMYV VRKRYQCMRD
duck MYO5A      .......V.. .K........ ...Y...... .......... T......... ..........

                       850        860        870        880        890        900
                ....|....| ....|....| ....|....| ....|....| ....|....| ....|....|
chick MYO5A     ATIALQALLR GYLVRNKYQM MLREHKSIII QKHVRGWLAR VHYHRTLKAI VYLQCCYRRM
duck MYO5A      .......... ..MA...... .......... .......... ...R...... ..........

                       910        920        930        940        950        960
                ....|....| ....|....| ....|....| ....|....| ....|....| ....|....|
chick MYO5A     MAKRELKKLK IEARSVERYK KLHIGLENKI MQLQRKIDEQ NKEYKSLLEK MNNLEITYST
duck MYO5A      .......... .......... .......... .......... .......... L.S.......

                       970        980        990       1000       1010       1020
                ....|....| ....|....| ....|....| ....|....| ....|....| ....|....|
chick MYO5A     ETEKLRSDVE RLRMSEEEAK NATNRVLSLQ EEIAKLRKEL HQTQTEKKTI EEWADKYKHE
duck MYO5A      ...R...... .......... .......... .......... ....S..... ..........

                      1030       1040       1050       1060       1070       1080
                ....|....| ....|....| ....|....| ....|....| ....|....| ....|....|
chick MYO5A     TEQLVSELKE QNTLLKTEKE ELNRRIHDQA KEITETMEKK LVEETKQLEL DLNDERLRYQ
duck MYO5A      .........D .......... .......... .....A.... .......... ..........

                      1090       1100       1110       1120       1130       1140
                ....|....| ....|....| ....|....| ....|....| ....|....| ....|....|
```

```
chick MYO5A  NLLNEFSRLE ERYDDLKDEM NLMVSIPKPG HKRTDSTHSS NESEYTFSSE ITEAEDLPLR
duck MYO5A   .......... .......... .......... .......... .......... ..........

                   1150       1160       1170       1180       1190       1200
             ....|....| ....|....| ....|....| ....|....| ....|....| ....|....|
chick MYO5A  ME-EPSEKKA PLDMSLFLKL QKRVTELEQE KQSLQDELDR KEEQALRAKA KEEERPPIRG
duck MYO5A   ..Q....... .......... .......... .......... .......... ..........

                   1210       1220       1230       1240       1250       1260
             ....|....| ....|....| ....|....| ....|....| ....|....| ....|....|
chick MYO5A  AELEYESLKR QELESENKKL KNELNELQKA LTETRAPEVT APGAPAYRVL LDQLTSVSEE
duck MYO5A   .......... .......... .......... .....S.... .......... ..........

                   1270       1280       1290       1300       1310       1320
             ....|....| ....|....| ....|....| ....|....| ....|....| ....|....|
chick MYO5A  LEVRKEEVLI LRSQLVSQKE AIQPKEDKNT MTDSTILLED VQKMKDKGEI AQAYIGLKET
duck MYO5A   .......... .......... .....---.. .......... .......... ..........

                   1330       1340       1350       1360       1370       1380
             ....|....| ....|....| ....|....| ....|....| ....|....| ....|....|
chick MYO5A  N--------- ---------- --------RL LESQLQSQKK SHENELESLR GEIQSLKEEN
duck MYO5A   .RQSPQDYHM LNEDGELWLV YEGLKQAN.. .......... .......... ..........

                   1390       1400       1410       1420       1430       1440
             ....|....| ....|....| ....|....| ....|....| ....|....| ....|....|
chick MYO5A  NRQQQLLAQN LQLPPEARIE ASLQHEITRL TNEN------ ---------- ---------L
duck MYO5A   .......... .......... .......... ....LFYEEL YADDPKKYQS YRISLYKRMI

                   1450       1460       1470       1480       1490       1500
             ....|....| ....|....| ....|....| ....|....| ....|....| ....|....|
chick MYO5A  DLMEQLEKQD KTVRKLKKQL KVFAKKIGEL EVGQMENISP GQIIDEPIRP VNIPRKEKDF
duck MYO5A   .......... .......... .......... .......... .......... ..........

                   1510       1520       1530       1540       1550       1560
             ....|....| ....|....| ....|....| ....|....| ....|....| ....|....|
chick MYO5A  QGMLEYKKED EQKLVKNLIL ELKPRGVAVN LIPGLPAYIL FMCVRHADYL NDDQKVRSLL
duck MYO5A   .......... .......... .......... .......... .......... ..........

                   1570       1580       1590       1600       1610       1620
             ....|....| ....|....| ....|....| ....|....| ....|....| ....|....|
chick MYO5A  TSTINGIKKV LKKRGDDFET VSFWLSNTCR FLHCLKQYSG EEGFMKHNTP RQNEHCLTNF
duck MYO5A   .......... .......... .......... .......... .......... ..........

                   1630       1640       1650       1660       1670       1680
             ....|....| ....|....| ....|....| ....|....| ....|....| ....|....|
chick MYO5A  DLAEYRQVLS DLAIQIYQQL VRVLENILQP MIVSGMLEHE TIQGVSGVKP TGLRKRTSSI
duck MYO5A   .......... .......... .......... .......... .......... ..........

                   1690       1700       1710       1720       1730       1740
             ....|....| ....|....| ....|....| ....|....| ....|....| ....|....|
chick MYO5A  ADEGTYTLDS IIRQLNSFHS VMCQHGMDPE LIKQVVKQMF YIIGAVTLNN LLLRKDMCSW
duck MYO5A   .......... .......... .......... .......... .......... ..........

                   1750       1760       1770       1780       1790       1800
             ....|....| ....|....| ....|....| ....|....| ....|....| ....|....|
chick MYO5A  SKGMQIRYNV SQLEEWLRDK NLMNSGAKET LEPLIQAAQL LQVKKKTDED AEAICSMCNA
duck MYO5A   .......... .......... .......... .......... .......... ..........

                   1810       1820       1830       1840       1850       1860
             ....|....| ....|....| ....|....| ....|....| ....|....| ....|....|
chick MYO5A  LTTAQIVKVL NLYTPVNEFE ERVLVSFIRT IQLRLRDRKD SPQLLMDAKH IFPVTFPFNP
duck MYO5A   .......... .......... .......... .......... .......... ..........

                   1870       1880
             ....|....| ....|....| ..
chick MYO5A  SSLALETIQI PASLGLGFIS RV
duck MYO5A   .......... .......... ..
```
