# Supplementary material for: Proteomic Screening for Cellular Targets of the Duck Enteritis Virus Protein VP26 Reveals That the Host Actin–Myosin II Network Regulates the Proliferation of the Virus
Source: Int J Mol Sci. 2025 Sep 18;26(18):9108. doi: 10.3390/ijms26189108 (PMC12470233; doi:10.3390/ijms26189108)
Supplement: Supplementary file 1 [file ijms-26-09108-s001.zip › Supplement S4- Alignment of duck-original and chick-original protein sequences/TMED10.file.pdf]

https://www.uniprot.org/uniprotkb/F1NA76/entry

> Chick TMED10

MPLPPPGRPRLRLAPLLALLLLAGPARPISFQLPGKARKCLREEIHRDTLVTGEYEIGAPPGSSSGPSANL
KITDSAGHILYAKEDATKGKFAFTTEDYDMFEACFESKLPVGTGRMPDQLVILDMKHGVEAKNYEEIAKVE
KLKPLEVELRRLEDLSESIVNDFAYMKKREEEMRDTNESTNTRVLYFSIFSMCCLIGLATWQVFYLRRFFK
AKKLIE

https://www.ncbi.nlm.nih.gov/nuccore/XM_027458009.3

>duck TMED10

MPLLPLPPGRPRHSAGPGPVPRLAPLLLLLLLLLLLAGPARPIS

FQLPGKARKCLREEIHRDTLVTGEYEIGAPPGSSSGPSANLKITDSAGHILYAKEDAT

KGKFAFTTEDYDMFEACFESKLPVGTGRMPDQLVILDMKHGVEAKNYEEIAKVEKLKP

LEVELRRLEDLSESIVNDFAYMKKREEEMRDTNESTNTRVLYFSIFSMCCLIGLATWQ
VFYLRRFFKAKKLIE
