# Supplementary material for: Proteomic Screening for Cellular Targets of the Duck Enteritis Virus Protein VP26 Reveals That the Host Actin–Myosin II Network Regulates the Proliferation of the Virus
Source: Int J Mol Sci. 2025 Sep 18;26(18):9108. doi: 10.3390/ijms26189108 (PMC12470233; doi:10.3390/ijms26189108)
Supplement: Supplementary file 1 [file ijms-26-09108-s001.zip › Supplement S4- Alignment of duck-original and chick-original protein sequences/TMED10.pdf]

```
                      10         20         30         40         50         60
              ....|....| ....|....| ....|....| ....|....| ....|....| ....|....|
chick TMED10  MPLP--PPGR PR-------- LRLAP----L LALLLLAGPA RPISFQLPGK ARKCLREEIH
duck TMED10   ...LPL.... ..HSAGPGPV P....LLLL. .L........ .......... ..........

                      70         80         90        100        110        120
              ....|....| ....|....| ....|....| ....|....| ....|....| ....|....|
chick TMED10  RDTLVTGEYE IGAPPGSSSG PSANLKITDS AGHILYAKED ATKGKFAFTT EDYDMFEACF
duck TMED10   .......... .......... .......... .......... .......... ..........

                     130        140        150        160        170        180
              ....|....| ....|....| ....|....| ....|....| ....|....| ....|....|
chick TMED10  ESKLPVGTGR MPDQLVILDM KHGVEAKNYE EIAKVEKLKP LEVELRRLED LSESIVNDFA
duck TMED10   .......... .......... .......... .......... .......... ..........

                     190        200        210        220        230
              ....|....| ....|....| ....|....| ....|....| ....|....| ...
chick TMED10  YMKKREEEMR DTNESTNTRV LYFSIFSMCC LIGLATWQVF YLRRFFKAKK LIE
duck TMED10   .......... .......... .......... .......... .......... ...
```
