# Supplementary material for: Proteomic Screening for Cellular Targets of the Duck Enteritis Virus Protein VP26 Reveals That the Host Actin–Myosin II Network Regulates the Proliferation of the Virus
Source: Int J Mol Sci. 2025 Sep 18;26(18):9108. doi: 10.3390/ijms26189108 (PMC12470233; doi:10.3390/ijms26189108)
Supplement: Supplementary file 1 [file ijms-26-09108-s001.zip › Supplement S4- Alignment of duck-original and chick-original protein sequences/TMOD3.file.pdf]

https://www.ncbi.nlm.nih.gov/nuccore/2925845625

PREDICTED: Anas platyrhynchos tropomodulin-3 (LOC101792155), transcript variant X5, mRNA

NCBI Reference Sequence: XM_027466857.3

>duck TMOD3

MTLPFRKDLDKYKDLDEDEILGKLSEEELKQLETVLDDLDPENA

LLPAGFRQKDQTAKKASGPFDRERLLAYLEKQALEHKDREDYVPFTKEKKGKIFIPKQ

KPVQSFTEEKIALDPELEEALTSATDTELCDLAAILGMSNLITNNQFCDVVGSSNGVD

KDSFSNIVKGEKMLPVFDEPPNPTNVEETLQRIKDNDSRLVEVNLNNIKNIPIPTLKE

FAKALETNTHVKNFSLAATRSNDPVAVALADMLRVNTKLKSLNIESNFITGVGILALV

DALKDNETLTEIKIDNQRQQLGTLAEVEIAKMLEENTKILKFGYHFTQQGPRARAAAA
ITKNNDLVRKRRVEGDSQ

https://www.uniprot.org/uniprotkb/Q5ZLY3/entry

>chick TMOD3

MTLPFRKDLDKYKDLDEDDILGKLSEEELKQLETVLDDLDPENALLPAGFRQKDQTAKKASGPFDRERL
LAYLEKQALEHKDREDYVPFTKEKKGKVFIPKQKPAQSYAEEKIALDPELEEALTSATDTELCDLAAIL
GMSNLITNNQFCDIVGSSNGVGKDSFSNIVKGEKMLPVFDEPPNPTNVEETLQRIKDNDSRLVEVNLNN
IKNIPIPTLKEFAKALETNTHVKNFSLAATRSNDPVAVALADMLRVNTKLKSLNIESNFITGVGILALV
DALKDNETLTEIKIDNQRQQLGTLAEVEIAKMLEENTKILKFGYHFTQQGPRARAAAAITKNNDLVRKR
RVEGDGQ
