# Supplementary material for: Proteomic Screening for Cellular Targets of the Duck Enteritis Virus Protein VP26 Reveals That the Host Actin–Myosin II Network Regulates the Proliferation of the Virus
Source: Int J Mol Sci. 2025 Sep 18;26(18):9108. doi: 10.3390/ijms26189108 (PMC12470233; doi:10.3390/ijms26189108)
Supplement: Supplementary file 1 [file ijms-26-09108-s001.zip › Supplement S4- Alignment of duck-original and chick-original protein sequences/TMOD3.pdf]

```
                      10         20         30         40         50         60
              ....|....| ....|....| ....|....| ....|....| ....|....| ....|....|
duck TMOD3    MTLPFRKDLD KYKDLDEDEI LGKLSEEELK QLETVLDDLD PENALLPAGF RQKDQTAKKA
chick TMOD3   .......... ........D. .......... .......... .......... ..........

                      70         80         90        100        110        120
              ....|....| ....|....| ....|....| ....|....| ....|....| ....|....|
duck TMOD3    SGPFDRERLL AYLEKQALEH KDREDYVPFT KEKKGKIFIP KQKPVQSFTE EKIALDPELE
chick TMOD3   .......... .......... .......... ......V... ....A..YA. ..........

                     130        140        150        160        170        180
              ....|....| ....|....| ....|....| ....|....| ....|....| ....|....|
duck TMOD3    EALTSATDTE LCDLAAILGM SNLITNNQFC DVVGSSNGVD KDSFSNIVKG EKMLPVFDEP
chick TMOD3   .......... .......... .......... .I.......G .......... ..........

                     190        200        210        220        230        240
              ....|....| ....|....| ....|....| ....|....| ....|....| ....|....|
duck TMOD3    PNPTNVEETL QRIKDNDSRL VEVNLNNIKN IPIPTLKEFA KALETNTHVK NFSLAATRSN
chick TMOD3   .......... .......... .......... .......... .......... ..........

                     250        260        270        280        290        300
              ....|....| ....|....| ....|....| ....|....| ....|....| ....|....|
duck TMOD3    DPVAVALADM LRVNTKLKSL NIESNFITGV GILALVDALK DNETLTEIKI DNQRQQLGTL
chick TMOD3   .......... .......... .......... .......... .......... ..........

                     310        320        330        340        350
              ....|....| ....|....| ....|....| ....|....| ....|....| ..
duck TMOD3    AEVEIAKMLE ENTKILKFGY HFTQQGPRAR AAAAITKNND LVRKRRVEGD SQ
chick TMOD3   .......... .......... .......... .......... .......... G.
```
