# Supplementary material for: Proteomic Screening for Cellular Targets of the Duck Enteritis Virus Protein VP26 Reveals That the Host Actin–Myosin II Network Regulates the Proliferation of the Virus
Source: Int J Mol Sci. 2025 Sep 18;26(18):9108. doi: 10.3390/ijms26189108 (PMC12470233; doi:10.3390/ijms26189108)
Supplement: Supplementary file 1 [file ijms-26-09108-s001.zip › Supplement S4- Alignment of duck-original and chick-original protein sequences/VAMP3.pdf]

```
                      10         20         30         40         50         60
              ....|....| ....|....| ....|....| ....|....| ....|....| ....|....|
chick VAMP3   MSANVPGNTN VPAGSNRRLQ QTQHQVDEVV DIMRVNVDKV LERDQKLSEL DDRADALQAG
duck VAMP3    ...S...SS. AA........ .......... .......... .......... ..........

                      70         80         90        100
              ....|....| ....|....| ....|....| ....|....| ....
chick VAMP3   ASQFETSAAK LKRKYWWKNC KMWAILIAVV VIIIIIIIVV SVSK
duck VAMP3    .......... .......... .......... .F.......W TAFS
```
