# Supplementary material for: Proteomic Screening for Cellular Targets of the Duck Enteritis Virus Protein VP26 Reveals That the Host Actin–Myosin II Network Regulates the Proliferation of the Virus
Source: Int J Mol Sci. 2025 Sep 18;26(18):9108. doi: 10.3390/ijms26189108 (PMC12470233; doi:10.3390/ijms26189108)
Supplement: Supplementary file 1 [file ijms-26-09108-s001.zip › Supplement S4- Alignment of duck-original and chick-original protein sequences/Xirp 1.pdf]

```
                     10         20         30         40         50         60
             ....|....| ....|....| ....|....| ....|....| ....|....| ....|....|
chick Xirp1  ---------- ---------- ---------- ---------- ---------- ----------
duck Xirp1   MEKAEKLRPA QSLPFLCHSP RGIPEHEAVP LRKSVSVSEL VARYQSILDC ESKMSKKEYP

                     70         80         90        100        110        120
             ....|....| ....|....| ....|....| ....|....| ....|....| ....|....|
chick Xirp1  ---------- ---------- ---------- ---------- ---------- ----------
duck Xirp1   KLMERRYLSQ TNGNPMGKKY GLSQSHRGDV SWSKSTEDLP IHKISATTRN LQGLPTPFDT

                    130        140        150        160        170        180
             ....|....| ....|....| ....|....| ....|....| ....|....| ....|....|
chick Xirp1  ---------- ---------- ---------- ---------- ---------- ----------
duck Xirp1   HRANPQSNTL PFAPLKTPPH NRILKKREAD TRILTTIQPL SRETKPHREP SFPSSLQSMQ

                    190        200        210        220        230        240
             ....|....| ....|....| ....|....| ....|....| ....|....| ....|....|
chick Xirp1  ---------- ---------- ---------- ---------- ---------- ----------
duck Xirp1   RMQWSPTTIT WKKSASKEGK ISRDRQVIIS SVPDAASDSA TGRSYERTRS FLDVSDSTRR

                    250        260        270        280        290        300
             ....|....| ....|....| ....|....| ....|....| ....|....| ....|....|
chick Xirp1  ---------- ---------- ---------- ---------- ---------- -----MAEPQ
duck Xirp1   ILQQGRGRCS SLSVKELSAR YLSQAAAAAA HGGPAQPTTV KDSSTPSSDR QKTSK...A.

                    310        320        330        340        350        360
             ....|....| ....|....| ....|....| ....|....| ....|....| ....|....|
chick Xirp1  KSSKVAIKKM EDDLPPPPIP DSIQVIAPAS QDPNPLPVPP PKQAFSKFYQ QRQVNELKRL
duck Xirp1   ...Q...... .E......AL G.V...P.G. .......... .......... ..........

                    370        380        390        400        410        420
             ....|....| ....|....| ....|....| ....|....| ....|....| ....|....|
chick Xirp1  YRHMHPELRK NLEEAVTEDL AEMLNTEDPN AQGSVNLDKV LPGEVQSMRW IFENWALDSI
duck Xirp1   .......... .......... .....A.... .......... .......... ..........

                    430        440        450        460        470        480
             ....|....| ....|....| ....|....| ....|....| ....|....| ....|....|
chick Xirp1  GDHQATKKMM EDEIIPGGDV KSTSLRFENQ SVNGDYLSTT AKVSETDLAR GDVHTARWLF
duck Xirp1   ........LT .E........ ........S. .I...S...P ..D....... ..........

                    490        500        510        520        530        540
             ....|....| ....|....| ....|....| ....|....| ....|....| ....|....|
chick Xirp1  ETQPLDSLNK LYSDETEMQE AVLKEPVQGG DVKGAKELFE AQSLDAIGRC CSVEEKSILQ
duck Xirp1   .......... .......V.. .......... .....R.... .......... ..........

                    550        560        570        580        590        600
             ....|....| ....|....| ....|....| ....|....| ....|....| ....|....|
chick Xirp1  LKSEIQELKG DVKKTIRLFQ TEPLCAIRDK TGNIHEIKSV CREEIQSNAV RTARWLFETQ
duck Xirp1   .......... .......... .......... .......... .......... ..........

                    610        620        630        640        650        660
             ....|....| ....|....| ....|....| ....|....| ....|....| ....|....|
chick Xirp1  PLDTINKDTS KVQIIRGISL EEIGRPDVSG ARWIFETQPL DAIREITVEE QDFKASTDFV
duck Xirp1   .......... .......... .......... .......... .......... ..........

                    670        680        690        700        710        720
             ....|....| ....|....| ....|....| ....|....| ....|....| ....|....|
chick Xirp1  TGADVTKQRL LFGTQALDSL KGEASESVAA KEQVIGGDVK STLWLFETQP METLKDNVEV
duck Xirp1   .....S.... ..E..T.... ...D..T... .......... .......... .......F..

                    730        740        750        760        770        780
             ....|....| ....|....| ....|....| ....|....| ....|....| ....|....|
chick Xirp1  GHLKKVELSA EEKGDVKQRK HVFETCPLGS ISKAFEEEIS AASTEEVVKG DVKSFKTLFE
duck Xirp1   .R........ .......... .......... ...S....T. ET.R...... ..........

                    790        800        810        820        830        840
             ....|....| ....|....| ....|....| ....|....| ....|....| ....|....|
chick Xirp1  TLPLDSIKEV DAEPITKEEE KIPPGNVKAN QILFETTPLY AIKDSFGNFH EVTSVSREQV
duck Xirp1   .........A .......... ...A...... .......... .......... ..........

                    850        860        870        880        890        900
             ....|....| ....|....| ....|....| ....|....| ....|....| ....|....|
chick Xirp1  ISGDVKKYKW MFETRPLDQF DESTKKVDII RGITKQEVVA GDVRTAKWLF ETQPMDVIHH
duck Xirp1   ......N... .......... .......... .......... .......... ....I.....

                    910        920        930        940        950        960
             ....|....| ....|....| ....|....| ....|....| ....|....| ....|....|
chick Xirp1  QATQGEEHPS MKREISQRGD VKTCRWLFET QPMHTLYEKA EKKQEEDVSV PQADVKSYTW
duck Xirp1   ..M.....S. V......... .......... ..I......S ........C. ..........

                    970        980        990       1000       1010       1020
             ....|....| ....|....| ....|....| ....|....| ....|....| ....|....|
chick Xirp1  MFETQPLDSL KGQEEQYLRV SKAYSQDELQ GVDVKTVRHL FETEPLGSSV VSEADQKKTL
duck Xirp1   .......... ........K. ......E... .......... .........T I........M

                   1030       1040       1050       1060       1070       1080
             ....|....| ....|....| ....|....| ....|....| ....|....| ....|....|
chick Xirp1  RYSSRVEIQS GEVSRVKEFF EAKPLDTTTK PTAVIKDDGT IEAGSVHKFT WLFENYPMDT
duck Xirp1   .......... .......... .......... ....V..... .......... .........S

                   1090       1100       1110       1120       1130       1140
             ....|....| ....|....| ....|....| ....|....| ....|....| ....|....|
```

```
chick Xirp1   LKDSSEGIQE IPPEKDIKGG DVGGKRFIFE TYSLDQIHDK VDETELHKIQ KDTMSKANVK
duck Xirp1    .......... .......... .......... .......... ......Q... ..........

                    1150       1160       1170       1180       1190       1200
              ....|....| ....|....| ....|....| ....|....| ....|....| ....|....|
chick Xirp1   SCTMLFESQP LYAIQDKEGG YHEVTSVQKE EIMKGDVKGA RWLFETKPLD QIKKEEEVFV
duck Xirp1    .......... .......... .......... .......... .......... ..........

                    1210       1220       1230       1240       1250       1260
              ....|....| ....|....| ....|....| ....|....| ....|....| ....|....|
chick Xirp1   IRAVTQEDIK KGDVQAARWR FETEPLDSFP GGKISVPRTV DDVQKGDVQS NKQLFESQQV
duck Xirp1    .......... .......... .........S ...T...... .......... ..........

                    1270       1280       1290       1300       1310       1320
              ....|....| ....|....| ....|....| ....|....| ....|....| ....|....|
chick Xirp1   GQKKYVRMVS VSDVQRGDVR TSTWLFENQP VDSLYGDADR SSSISTVQRE DSQKGDVKRC
duck Xirp1    .......... .......... .......... ....H..... .......... ..........

                    1330       1340       1350       1360       1370       1380
              ....|....| ....|....| ....|....| ....|....| ....|....| ....|....|
chick Xirp1   TWLFETQPMD TLKDPEVTVS TGTQEPIPRA DVKSTTWLFE STPLDKFSAS ECSRETELKE
duck Xirp1    .......... ....T...AT A.A..A.... .......... .......... .G.G......

                    1390       1400       1410       1420       1430       1440
              ....|....| ....|....| ....|....| ....|....| ....|....| ....|....|
chick Xirp1   RTMRETLETL CTCQAIQHDG ILIEANDTES VKMVKYQLSS PGAPEILKEE IVRGHLQGIM
duck Xirp1    ...K...... .......... .......M.. ........IT .......... ..G....R..

                    1450       1460       1470       1480       1490       1500
              ....|....| ....|....| ....|....| ....|....| ....|....| ....|....|
chick Xirp1   LQLLHRTNVE PQSVLVEEDR EGKIKVSSLQ LLDQSEAIKG KEDLSGNVAK ALQSLLSQDA
duck Xirp1    .......... A......... .......... .......V.S .......... ...G......

                    1510       1520       1530       1540       1550       1560
              ....|....| ....|....| ....|....| ....|....| ....|....| ....|....|
chick Xirp1   SIKKGMVIQE TKSESVKMTL YSLLFHSVQQ KVVKGDVKST IGNLMASSQE QRATVTVKRE
duck Xirp1    .......L.. ...G.M.... .......... .......... ....L..... .K..A.....

                    1570       1580       1590       1600       1610       1620
              ....|....| ....|....| ....|....| ....|....| ....|....| ....|....|
chick Xirp1   DNEKGNVQLF ASCIEKGDLD YLKNLQQESE IQSLISAQAE QGAAESAPRA LQSTNTHVLA
duck Xirp1    .......... .......... .......... ......S... ..VD..V.-L V.GAKI....

                    1630       1640       1650       1660       1670       1680
              ....|....| ....|....| ....|....| ....|....| ....|....| ....|....|
chick Xirp1   NKEQVEKVMA EAKSGALEGA KMVFACESTG KEGALEREVV HAVGVTGTTV QCLGKPQNLP
duck Xirp1    ..D.A...I. .GE...M... .K..V...V. .....QK.AM ..A....A.. .......S..

                    1690       1700       1710       1720       1730       1740
              ....|....| ....|....| ....|....| ....|....| ....|....| ....|....|
chick Xirp1   TGMEKEEIMS GGLKVTTKSI QRVADVSKNT EKEESISACL KEPKATMQGI AQAKVTAERN
duck Xirp1    .A.G....L. .......... ........KA .....T..S. .....MT..T T.TQ..V..G

                    1750       1760       1770       1780       1790       1800
              ....|....| ....|....| ....|....| ....|....| ....|....| ....|....|
chick Xirp1   EVVGEQQSLV TEQASQKQSE EKVLGNDLQA AMQSLRLATA EARNIQHHVQ SKLQRNREEV
duck Xirp1    ..A..H...M .GP...M.PG .....S.... .......... ..K....... ....K.....

                    1810       1820       1830       1840       1850       1860
              ....|....| ....|....| ....|....| ....|....| ....|....| ....|....|
chick Xirp1   HMACRQQVAS KQETKTLQST IHQQESASTM RENTSTAIRT STTRVQEASR THTSVSQKSI
duck Xirp1    .---...A.. M.G....... ....D....T ..S....... T........Q S...M.....

                    1870       1880       1890       1900       1910       1920
              ....|....| ....|....| ....|....| ....|....| ....|....| ....|....|
chick Xirp1   ASHKKVSASE EVQGGQLLSQ ENQVVPSRDV SIKDGLYTAT PVKTYINPFV ESDYKEQSVQ
duck Xirp1    .......... .......... ..R....... .......... .......... ..........

                    1930       1940       1950       1960       1970       1980
              ....|....| ....|....| ....|....| ....|....| ....|....| ....|....|
chick Xirp1   EERDVIIRGD VQTAIRALQS AATEQRLVEK EDIVRGNLKA TLQSLEKSNV NVSKGDFKAA
duck Xirp1    .......... .......... .......... ..V....... .......... ...R......

                    1990       2000       2010       2020       2030       2040
              ....|....| ....|....| ....|....| ....|....| ....|....| ....|....|
chick Xirp1   MIYRNAGQSY SVCKKKSETQ VNNNQTAVVA SGSQADNDFP PPPPVAVMKA EHCPPSTKAT
duck Xirp1    .......... ......N... .IS....... .......... .......... ......A...

                    2050       2060       2070       2080       2090       2100
              ....|....| ....|....| ....|....| ....|....| ....|....| ....|....|
chick Xirp1   REGAPPLLTS KDEAPGCFSP LQTPLPPPPS LSCKPSDQNS TEKPKIPPKP EITAPLRKKP
duck Xirp1    G...L.PPAC .......SA. I.....TL.. ......E..A A.....S..S .....P....

                    2110       2120       2130       2140       2150       2160
              ....|....| ....|....| ....|....| ....|....| ....|....| ....|....|
chick Xirp1   VPPPKPEHLL HEAYSASTNN STNRSTKSVP PPVPPKPPGL REISMPKPPP AELQLSCTEV
duck Xirp1    .......... ....C...I. ..S....P.. ..L....Q.. ..VGK....A ...R.G.V..

                    2170       2180       2190       2200       2210       2220
              ....|....| ....|....| ....|....| ....|....| ....|....| ....|....|
chick Xirp1   CEQSDHRESQ DKCCTLESSM DKSITVHG-- PERKLPKYTA KTPLQMAEER YKARKGGQGK
duck Xirp1    ....G.V.G. A......... E..V..Q.MS .......D.. .....L.... ..TS..R.C.
```

```
                      2230       2240       2250       2260       2270       2280
               ....|....| ....|....| ....|....| ....|....| ....|....| ....|....|
chick Xirp1    FELDRAKPSK PVKNGEVG-- ---------- ---------- ---------- ----------
duck Xirp1     S.V.S..T.. ..Q..V..FQ VEQGTMSGKA AAPGSCLGKV VQRHSELYHQ EDRFSSVSHP

                      2290       2300       2310       2320       2330       2340
               ....|....| ....|....| ....|....| ....|....| ....|....| ....|....|
chick Xirp1    ---------- ---------- ---------- ---------- ---------- ----------
duck Xirp1     ACPGRAQTLN VPGQTEPSTS SVGRSTPPKR GDDTSKNALP KVERESVYNA YMSWDSQRVT

                      2350       2360       2370       2380       2390       2400
               ....|....| ....|....| ....|....| ....|....| ....|....| ....|....|
chick Xirp1    ---------- ---------- ---------- ---------- ---------- ----------
duck Xirp1     QQVSERRQTS HSMSFHQQPV NPSKEEHQGN SGQQKCPDGE AEAPAQEKPA VIMREKPKKE

                      2410       2420       2430       2440       2450       2460
               ....|....| ....|....| ....|....| ....|....| ....|....| ....|....|
chick Xirp1    ---------- ---------- ---------- ---------- ---------- ----------
duck Xirp1     TEDERRKRLS VHKEEIMKGN VKEAMEIFEN LRRQEELQEI LTRVKEFEEE TSKVDVKALR

                      2470       2480       2490       2500       2510       2520
               ....|....| ....|....| ....|....| ....|....| ....|....| ....|....|
chick Xirp1    ---------- ---------- ---------- ---------- ---------- ----------
duck Xirp1     SFFEKVPDWV VRQKTTQAKQ QDRAETQAKD DADSVSSVEL VFGDLERASA EIIHLKEQTL

                      2530       2540       2550       2560       2570       2580
               ....|....| ....|....| ....|....| ....|....| ....|....| ....|....|
chick Xirp1    ---------- ---------- ---------- ---------- ---------- ----------
duck Xirp1     ARLLDIEEAI KKALYSVSSL KSESDIAGLS GLFKESLGNT QSSVSSSNIR KISIVSSKAR

                      2590       2600       2610       2620       2630       2640
               ....|....| ....|....| ....|....| ....|....| ....|....| ....|....|
chick Xirp1    ---------- ---------- ---------- ---------- ---------- ----------
duck Xirp1     QDGATVETGE AASGGGAKVA EKTEVTKSEL EVPRLVHPRV SSPSSPSYIS IESAARKPAE

                      2650       2660       2670       2680       2690       2700
               ....|....| ....|....| ....|....| ....|....| ....|....| ....|....|
chick Xirp1    ---------- ---------- ---------- ---------- ---------- ----------
duck Xirp1     SPRTAHSPRD MASPDCPDAP GKRDAFAQDG FSSFNHPSAG SAGRDKAPFE KKSEPTQTNT

                      2710       2720       2730       2740       2750       2760
               ....|....| ....|....| ....|....| ....|....| ....|....| ....|....|
chick Xirp1    ---------- ---------- ---------- ---------- ---------- ----------
duck Xirp1     GLNSVKQHNL GNTNHQVSEK EKCPPDTSKN SCHCGMKGGF PEYCSLNTPS PQNPRRQKSI

                      2770       2780       2790       2800       2810       2820
               ....|....| ....|....| ....|....| ....|....| ....|....| ....|....|
chick Xirp1    ---------- ---------- ---------- ---------- ---------- ----------
duck Xirp1     LELQTGPDGS KLYGATRTVT EQYEEMDQFG NKIITSSTTV TKQSETQTSS TCDVVSHPRY

                      2830       2840       2850       2860
               ....|....| ....|....| ....|....| ....|....| ..
chick Xirp1    ---------- ---------- ---------- ---------- --
duck Xirp1     EVSASPLFRR YVKSPGEDFH TNGSFQEPGV VFVTFGNSKP KK
```
