# Supplementary material for: Proteomic Screening for Cellular Targets of the Duck Enteritis Virus Protein VP26 Reveals That the Host Actin–Myosin II Network Regulates the Proliferation of the Virus
Source: Int J Mol Sci. 2025 Sep 18;26(18):9108. doi: 10.3390/ijms26189108 (PMC12470233; doi:10.3390/ijms26189108)
Supplement: Supplementary file 1 [file ijms-26-09108-s001.zip › Supplement S4- Alignment of duck-original and chick-original protein sequences/Xirp1.file.pdf]

https://www.uniprot.org/uniprotkb/Q91957/entry

>chick Xirp1

MAEPQKSSKVAIKKMEDDLPPPPIPDSIQVIAPASQDPNPLPVPPPKQAFSKFYQQRQVNELKRLYRHMHP
ELRKNLEEAVTEDLAEMLNTEDPNAQGSVNLDKVLPGEVQSMRWIFENWALDSIGDHQATKKMMEDEIIPG
GDVKSTSLRFENQSVNGDYLSTTAKVSETDLARGDVHTARWLFETQPLDSLNKLYSDETEMQEAVLKEPVQ
GGDVKGAKELFEAQSLDAIGRCCSVEEKSILQLKSEIQELKGDVKKTIRLFQTEPLCAIRDKTGNIHEIKS
VCREEIQSNAVRTARWLFETQPLDTINKDTSKVQIIRGISLEEIGRPDVSGARWIFETQPLDAIREITVEE
QDFKASTDFVTGADVTKQRLLFGTQALDSLKGEASESVAAKEQVIGGDVKSTLWLFETQPMETLKDNVEVG
HLKKVELSAEEKGDVKQRKHVFETCPLGSISKAFEEEISAASTEEVVKGDVKSFKTLFETLPLDSIKEVDA
EPITKEEEKIPPGNVKANQILFETTPLYAIKDSFGNFHEVTSVSREQVISGDVKKYKWMFETRPLDQFDES
TKKVDIIRGITKQEVVAGDVRTAKWLFETQPMDVIHHQATQGEEHPSMKREISQRGDVKTCRWLFETQPMH
TLYEKAEKKQEEDVSVPQADVKSYTWMFETQPLDSLKGQEEQYLRVSKAYSQDELQGVDVKTVRHLFETEP
LGSSVVSEADQKKTLRYSSRVEIQSGEVSRVKEFFEAKPLDTTTKPTAVIKDDGTIEAGSVHKFTWLFENY
PMDTLKDSSEGIQEIPPEKDIKGGDVGGKRFIFETYSLDQIHDKVDETELHKIQKDTMSKANVKSCTMLFE
SQPLYAIQDKEGGYHEVTSVQKEEIMKGDVKGARWLFETKPLDQIKKEEEVFVIRAVTQEDIKKGDVQAAR
WRFETEPLDSFPGGKISVPRTVDDVQKGDVQSNKQLFESQQVGQKKYVRMVSVSDVQRGDVRTSTWLFENQ
PVDSLYGDADRSSSISTVQREDSQKGDVKRCTWLFETQPMDTLKDPEVTVSTGTQEPIPRADVKSTTWLFE
STPLDKFSASECSRETELKERTMRETLETLCTCQAIQHDGILIEANDTESVKMVKYQLSSPGAPEILKEEI
VRGHLQGIMLQLLHRTNVEPQSVLVEEDREGKIKVSSLQLLDQSEAIKGKEDLSGNVAKALQSLLSQDASI
KKGMVIQETKSESVKMTLYSLLFHSVQQKVVKGDVKSTIGNLMASSQEQRATVTVKREDNEKGNVQLFASC
IEKGDLDYLKNLQQESEIQSLISAQAEQGAAESAPRALQSTNTHVLANKEQVEKVMAEAKSGALEGAKMVF
ACESTGKEGALEREVVHAVGVTGTTVQCLGKPQNLPTGMEKEEIMSGGLKVTTKSIQRVADVSKNTEKEES
ISACLKEPKATMQGIAQAKVTAERNEVVGEQQSLVTEQASQKQSEEKVLGNDLQAAMQSLRLATAEARNIQ
HHVQSKLQRNREEVHMACRQQVASKQETKTLQSTIHQQESASTMRENTSTAIRTSTTRVQEASRTHTSVSQ
KSIASHKKVSASEEVQGGQLLSQENQVVPSRDVSIKDGLYTATPVKTYINPFVESDYKEQSVQEERDVIIR
GDVQTAIRALQSAATEQRLVEKEDIVRGNLKATLQSLEKSNVNVSKGDFKAAMIYRNAGQSYSVCKKKSET
QVNNNQTAVVASGSQADNDFPPPPPVAVMKAEHCPPSTKATREGAPPLLTSKDEAPGCFSPLQTPLPPPPS
LSCKPSDQNSTEKPKIPPKPEITAPLRKKPVPPPKPEHLLHEAYSASTNNSTNRSTKSVPPPVPPKPPGLR
EISMPKPPPAELQLSCTEVCEQSDHRESQDKCCTLESSMDKSITVHGPERKLPKYTAKTPLQMAEERYKAR
KGGQGKFELDRAKPSKPVKNGEVG

https://www.ncbi.nlm.nih.gov/nuccore/XM_038173952.2

>duck Xirp1

MEKAEKLRPAQSLPFLCHSPRGIPEHEAVPLRKSVSVSELVARY

QSILDCESKMSKKEYPKLMERRYLSQTNGNPMGKKYGLSQSHRGDVSWSKSTEDLPIH

KISATTRNLQGLPTPFDTHRANPQSNTLPFAPLKTPPHNRILKKREADTRILTTIQPL

SRETKPHREPSFPSSLQSMQRMQWSPTTITWKKSASKEGKISRDRQVIISSVPDAASD

SATGRSYERTRSFLDVSDSTRRILQQGRGRCSSLSVKELSARYLSQAAAAAAHGGPAQ

PTTVKDSSTPSSDRQKTSKMAEAQKSSQVAIKKMEEDLPPPPALGSVQVIPPGSQDPN

PLPVPPPKQAFSKFYQQRQVNELKRLYRHMHPELRKNLEEAVTEDLAEMLNAEDPNAQ

GSVNLDKVLPGEVQSMRWIFENWALDSIGDHQATKKLTEEEIIPGGDVKSTSLRFESQ

SINGDSLSTPAKDSETDLARGDVHTARWLFETQPLDSLNKLYSDETEVQEAVLKEPVQ

GGDVKGARELFEAQSLDAIGRCCSVEEKSILQLKSEIQELKGDVKKTIRLFQTEPLCA

IRDKTGNIHEIKSVCREEIQSNAVRTARWLFETQPLDTINKDTSKVQIIRGISLEEIG

RPDVSGARWIFETQPLDAIREITVEEQDFKASTDFVTGADVSKQRLLFETQTLDSLKG

EDSETVAAKEQVIGGDVKSTLWLFETQPMETLKDNFEVGRLKKVELSAEEKGDVKQRK

HVFETCPLGSISKSFEEETSETSREEVVKGDVKSFKTLFETLPLDSIKEADAEPITKE

EEKIPAGNVKANQILFETTPLYAIKDSFGNFHEVTSVSREQVISGDVKNYKWMFETRP

LDQFDESTKKVDIIRGITKQEVVAGDVRTAKWLFETQPIDVIHHQAMQGEEHSSVKRE

ISQRGDVKTCRWLFETQPIHTLYEKSEKKQEEDVCVPQADVKSYTWMFETQPLDSLKG

QEEQYLKVSKAYSQEELQGVDVKTVRHLFETEPLGSSTISEADQKKTMRYSSRVEIQS

GEVSRVKEFFEAKPLDTTTKPTAVVKDDGTIEAGSVHKFTWLFENYPMDSLKDSSEGI

QEIPPEKDIKGGDVGGKRFIFETYSLDQIHDKVDETELQKIQKDTMSKANVKSCTMLF

ESQPLYAIQDKEGGYHEVTSVQKEEIMKGDVKGARWLFETKPLDQIKKEEEVFVIRAV

TQEDIKKGDVQAARWRFETEPLDSFSGGKTSVPRTVDDVQKGDVQSNKQLFESQQVGQ

KKYVRMVSVSDVQRGDVRTSTWLFENQPVDSLHGDADRSSSISTVQREDSQKGDVKRC

TWLFETQPMDTLKDTEVTATAGAQEAIPRADVKSTTWLFESTPLDKFSASEGSGETEL

KERTMKETLETLCTCQAIQHDGILIEANDMESVKMVKYQLITPGAPEILKEEIVGGHL

QRIMLQLLHRTNVEAQSVLVEEDREGKIKVSSLQLLDQSEAVKSKEDLSGNVAKALQG

LLSQDASIKKGMVLQETKSGSMKMTLYSLLFHSVQQKVVKGDVKSTIGNLLASSQEQK

ATATVKREDNEKGNVQLFASCIEKGDLDYLKNLQQESEIQSLISSQAEQGVDESVPLV

QGAKIHVLANKDQAEKVIAEGESGAMEGAKKVFVCESVGKEGALQKEAMHAAGVTGAT

VQCLGKPQSLPTAMGKEEILSGGLKVTTKSIQRVADVSKKAEKEESTSASLKEPKAMT

QGTTQTQVTVERGEVAGEHQSLMTGPASQMQPGEKVLGSDLQAAMQSLRLATAEAKNI

QHHVQSKLQKNREEVHRQQAASMQGTKTLQSTIHQQDSASTTRESTSTAIRTTTTRVQ

EASQSHTSMSQKSIASHKKVSASEEVQGGQLLSQENRVVPSRDVSIKDGLYTATPVKT

YINPFVESDYKEQSVQEERDVIIRGDVQTAIRALQSAATEQRLVEKEDVVRGNLKATL

QSLEKSNVNVSRGDFKAAMIYRNAGQSYSVCKKKNETQVISNQTAVVASGSQADNDFP

PPPPVAVMKAEHCPPSAKATGEGALPPPACKDEAPGCSAPIQTPLPTLPSLSCKPSEQ

NAAEKPKISPKSEITAPPRKKPVPPPKPEHLLHEAYCASTINSTSRSTKPVPPPLPPK

PQGLREVGKPKPPAAELRLGCVEVCEQSGHVEGQAKCCTLESSMEKSVTVQGMSPERK

LPKDTAKTPLQLAEERYKTSKGRQCKSEVDSAKTSKPVQNGVVGFQVEQGTMSGKAAA

PGSCLGKVVQRHSELYHQEDRFSSVSHPACPGRAQTLNVPGQTEPSTSSVGRSTPPKR

GDDTSKNALPKVERESVYNAYMSWDSQRVTQQVSERRQTSHSMSFHQQPVNPSKEEHQ

GNSGQQKCPDGEAEAPAQEKPAVIMREKPKKETEDERRKRLSVHKEEIMKGNVKEAME

IFENLRRQEELQEILTRVKEFEEETSKVDVKALRSFFEKVPDWVVRQKTTQAKQQDRA

ETQAKDDADSVSSVELVFGDLERASAEIIHLKEQTLARLLDIEEAIKKALYSVSSLKS

ESDIAGLSGLFKESLGNTQSSVSSSNIRKISIVSSKARQDGATVETGEAASGGGAKVA

EKTEVTKSELEVPRLVHPRVSSPSSPSYISIESAARKPAESPRTAHSPRDMASPDCPD

APGKRDAFAQDGFSSFNHPSAGSAGRDKAPFEKKSEPTQTNTGLNSVKQHNLGNTNHQ

VSEKEKCPPDTSKNSCHCGMKGGFPEYCSLNTPSPQNPRRQKSILELQTGPDGSKLYG

ATRTVTEQYEEMDQFGNKIITSSTTVTKQSETQTSSTCDVVSHPRYEVSASPLFRRYV

KSPGEDFHTNGSFQEPGVVFVTFGNSKPKK
