# Supplementary material for: Proteomic Screening for Cellular Targets of the Duck Enteritis Virus Protein VP26 Reveals That the Host Actin–Myosin II Network Regulates the Proliferation of the Virus
Source: Int J Mol Sci. 2025 Sep 18;26(18):9108. doi: 10.3390/ijms26189108 (PMC12470233; doi:10.3390/ijms26189108)
Supplement: Supplementary file 1 [file RPS26.pdf › Supplement S4- Alignment of duck-original and chick-original protein sequences/RPS26.pdf]

```
                       10         20         30         40         50         60
               ....|....| ....|....| ....|....| ....|....| ....|....| ....|....|
chick RPS26    MTKKRRNNGR AKKGRGHVQP IRCTNCARCV PKDKAIKKFV IRNIVEAAAV RDISEASVFD
duck RPS26     .......... .......... .......... .......... .......... ..........

                       70         80         90        100        110
               ....|....| ....|....| ....|....| ....|....| ....|....| ....|
chick RPS26    SYVLPKLYVK LHYCVSCAIH SKVVRNRSRE ARKDRTPPPR FRPAGAAPRP PPKPM
duck RPS26     .......... .......... .......... .......... .......... .....
```
